# Adhesive Virulence Factors of *Staphylococcus aureus* Resist Digestion by Coagulation Proteases Thrombin and Plasmin

**DOI:** 10.1021/acsbiomedchemau.2c00042

**Published:** 2022-09-02

**Authors:** Fanny Risser, Joanan López-Morales, Michael A. Nash

**Affiliations:** † Institute of Physical Chemistry, Department of Chemistry, University of Basel, 4058 Basel, Switzerland; ‡ Department of Biosystems Sciences and Engineering, ETH Zurich, 4058 Basel, Switzerland

**Keywords:** *S. aureus*, adhesin, MSCRAMM, staphylokinase, plasmin, thrombin, DEv-IgG fold

## Abstract

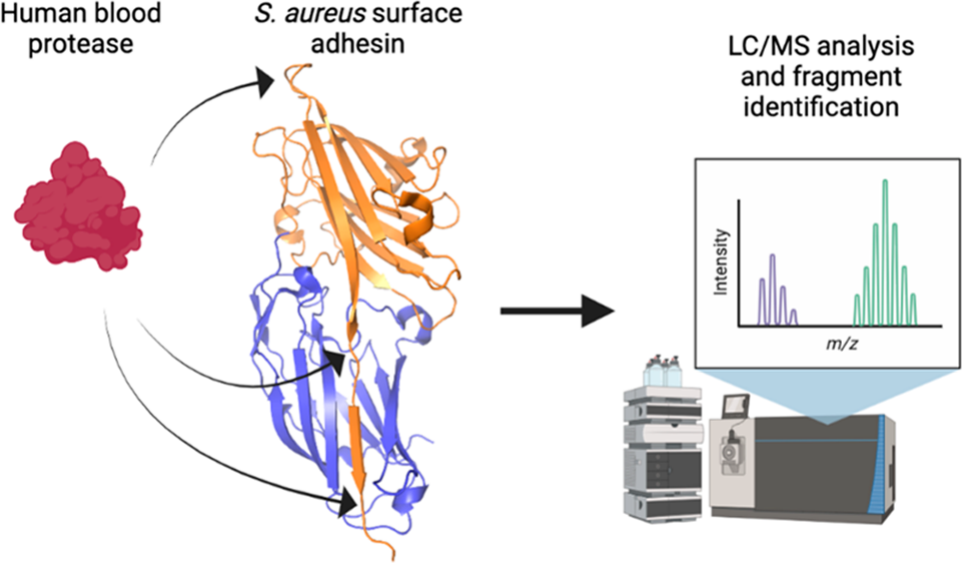

*Staphylococcus aureus* (*S. aureus*) is an invasive and life-threatening pathogen that has undergone extensive coevolution with its mammalian hosts. Its molecular adaptations include elaborate mechanisms for immune escape and hijacking of the coagulation and fibrinolytic pathways. These capabilities are enacted by virulence factors including microbial surface components recognizing adhesive matrix molecules (MSCRAMMs) and the plasminogen-activating enzyme staphylokinase (SAK). Despite the ability of *S. aureus* to modulate coagulation, until now the sensitivity of *S. aureus* virulence factors to digestion by proteases of the coagulation system was unknown. Here, we used protein engineering, biophysical assays, and mass spectrometry to study the susceptibility of *S. aureus* MSCRAMMs to proteolytic digestion by human thrombin, plasmin, and plasmin/SAK complexes. We found that MSCRAMMs were highly resistant to proteolysis, and that SAK binding to plasmin enhanced this resistance. We mapped thrombin, plasmin, and plasmin/SAK cleavage sites of nine MSCRAMMs and performed biophysical, bioinformatic, and stability analysis to understand structural and sequence features common to protease-susceptible sites. Overall, our study offers comprehensive digestion patterns of *S. aureus* MSCRAMMs by thrombin, plasmin, and plasmin/SAK complexes and paves the way for new studies into this resistance and virulence mechanism.

## Introduction

**Figure 1:**
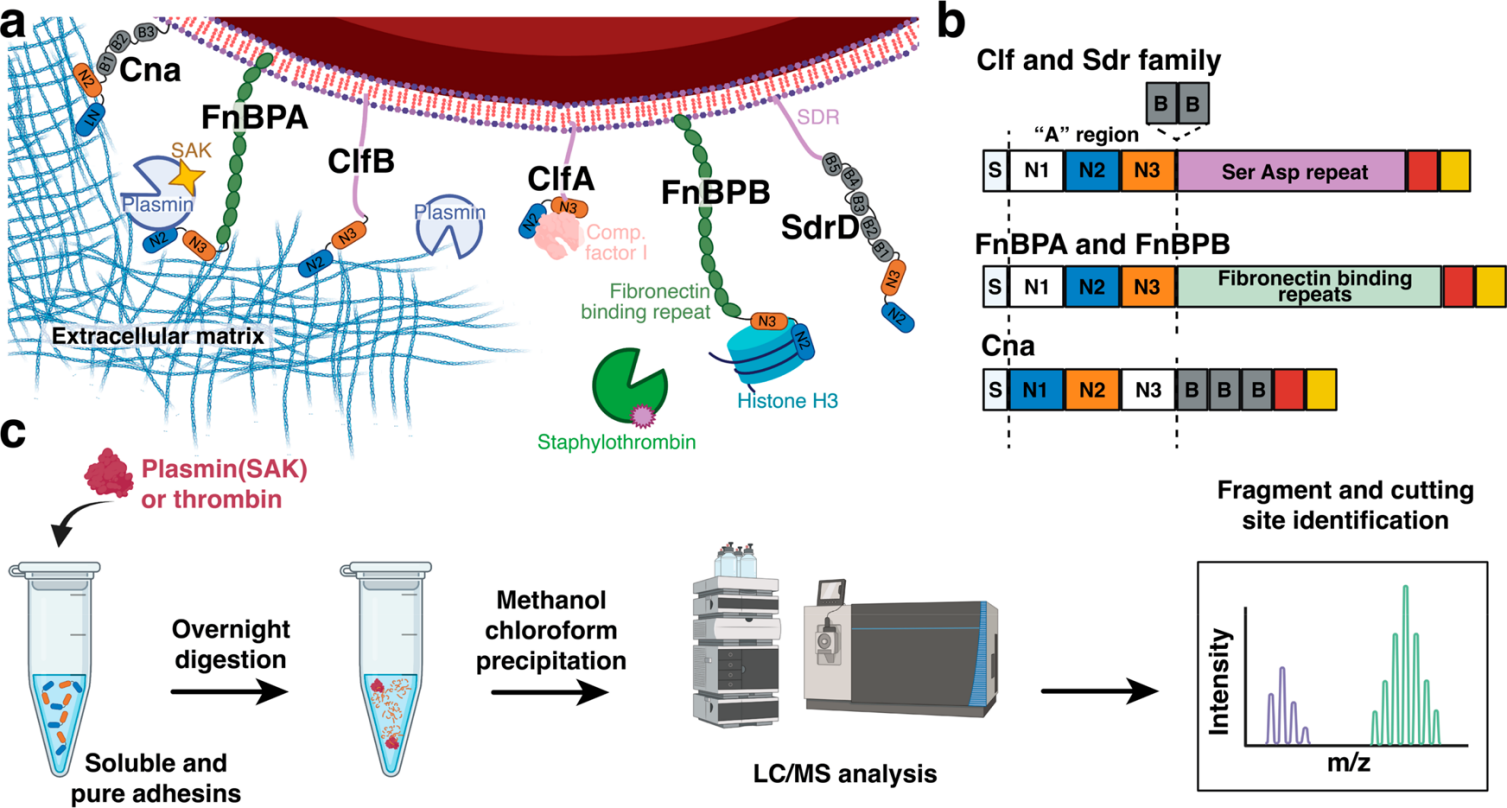
Overview of adhesive *S. aureus* virulence factors and design of blood protease susceptibility study. (a) Examples of MSCRAMMs harbored at the surface of *S. aureus* and analyzed in this work. These adhesins bind diverse extracellular matrix proteins, promote host colonization and immune escape, and modulate the coagulation cascade. (b) Domain organization of MSCRAMMs. They share a common architecture with a secretion signal peptide (S) at the N-terminus, followed by the A region comprising functional binding domains (blue and orange) N2 and N3, or in the case of Cna the N1 and N2 domains. Downstream of the A region are a variable number of B domains that serve as sacrificial domains under mechanical tension and flow. At the C-terminus, a cell wall-spanning region (red) and anchoring motif (yellow) facilitate covalent attachment to the bacterial surface. The Clf and Sdr subgroups both have characteristic Ser-Asp repeats (SDR) spanning from N3 (or variably B domains) to the C-terminus. FnBPA/B contain 10 to 11 fibronectin binding domains in place of the Ser-Asp repeats. (c) Scheme depicting the experimental procedure used to determine the proteolytic susceptibility of *S. aureus* adhesins. Recombinantly produced binding domains from the A region of various adhesins were exposed to thrombin, plasmin, or plasmin/SAK complexes in an overnight digest. The resulting mixture was analyzed by liquid chromatography coupled to mass spectrometry. After data processing, the mass of the resulting peaks was assigned to digestion fragments, and the cutting sites were localized within the adhesins’ sequence and structure.

*Staphylococcus aureus* is a Gram-positive opportunistic pathogen of the human upper respiratory tract carried by ∼30% of the population^1^ and responsible for a variety of pathologies ranging from minor skin infections to life threatening infective endocarditis and pneumonia.^2^ Its pathogenicity is partially explained by the presence of up to 24 different cell wall anchored proteins^3^ that are responsible for bacterial attachment to the extracellular matrix (ECM), biofilm formation, cell invasion, and immune evasion (Figure 1a).^4^ The most predominant family of *S. aureus* cell wall proteins is the microbial surface components recognizing adhesive matrix molecules (MSCRAMMs), also referred to as adhesins, which share a common architecture and binding mechanism.

There are at least three distinct subgroups of MSCRAMMs found at the surface of *S. aureus* (Figure 1b). All subgroups contain a signal peptide (S) at the N-terminus, followed by the A region responsible for ligand binding and a variable region. The A regions of these MSCRAMMs share 20 to 30% sequence identity and contain three N domains: N1, N2, and N3. Typically two of the N domains form the functional binding site by adopting a deviant immunoglobulin (DEv-Ig) fold which distinguishes itself from the classical Ig fold through additional β-strands.^5,6^ Following the A region, the MSCRAMM domain composition becomes more variable (Figure 1b), and only the C-termini are again homologous and shared among the different subgroups. The C-termini regions comprise a cell wall spanning region and an LPTXG sortase anchoring motif that facilitates covalent attachment of the adhesin to cell wall peptidoglycans.^7^ Thus far, ∼10 distinct MSCRAMMs have been identified and characterized in *S. aureus* (Table 1). Each *S. aureus* lineage possesses a particular combination of MSCRAMM variants at its surface.^8^ Some adhesins are constitutively expressed, like ClfA, while some are expressed only during specific periods of the *S. aureus* life cycle. For example, ClfB is only detectable in the early exponential phase.^3,9^ This surface variety is quite specific to *S. aureus* as opposed to other *Staphylococci* species.^10−12^

**Table 1:** Summary of Characterized *S. aureus* MSCRAMMs and Their Binding Partners

adhesin	binding partner(s) of the A region	biological role(s)
ClfA	fibrin(ogen), C-terminus γ-chain^26^	adhesion to fibrin(ogen) under high shear stress, formation of clumps and therefore immune evasion^27^
	fibrin(ogen), D region^28^	
	vWF and/or vWBP^29,30^	promotes cells and clumps arrest at damaged endothelium sites^31^
	complement factor 1^32,33^	degradation of complement factor C3b and therefore immune evasion^32^
ClfB	fibrin(ogen), αC region^34,35^	adhesion to fibrin(ogen) (less predominant than ClfA)^27^
	cytokeratin 10^36^	nasal colonization^27,37^
	loricrin^37^	
	corneodesmosin^38^	skin adhesion^38^
FnBPA	fibrin(ogen), C-terminus γ-chain^39^	adhesion to ECM^39−41^
	elastin^40,41^	
	plasmin(ogen)^20^	dissemination, immune evasion^42^
FnBPB	fibrin(ogen), C-terminus γ-chain^43^	adhesion to ECM^38,41,43,44^
	elastin^41^	
	fibronectin^43^	
	loricrin^44^	
	corneodesmosin^38^	
	histone H3^45^	dissemination, immune evasion^42^
	plasmin(ogen)^19,20^	
SdrC	β-neurexin^46^	unknown
SdrD	desmoglein-1^47^	adhesion to desquamated nasal epithelial cells^48^
SdrE	complement factor H^49^	immune evasion^49,50^
Cna	type 1 collagen^51,52^	adhesion to ECM^51,53,54^
	laminin^53^	
	complement factor C1q^52,53^	immune evasion^52^

MSCRAMMs are highly multivalent and promiscuous binders, and three or more binding partners have been identified for half of the adhesins studied here (Table 1). Many MSCRAMMs have been reported to bind fibrin(ogen),^13,14^ the soluble precursor of fibrin which constitutes the primary fibrous protein component of blood clots. Fibrin(ogen) binding activity is significant when considering the ability of *S. aureus* to modulate coagulation. Another significant feature of *S. aureus* compared to other *Staphylococci* species is its production and secretion of coagulases, which bind and activate (pro)thrombin (Figure 1a).^2,15^

On the one hand, *S. aureus* secretes coagulase and von Willebrand binding protein in order to activate prothrombin and promote the formation of fibrin clots,^16−18^ providing protection against host defense mechanisms.^2,15^ On the other hand, *S. aureus* FnBPs bind plasminogen with high affinity in the presence of fibrin^19,20^ and activate it by secreting staphylokinase (SAK; Figure 1a).^21^ Thus, the pathogen has the capacity not only to bind fibrinogen at its surface and assemble fibrin networks but also to activate fibrin degradation through plasmin.

The plasmin/SAK interaction involves the SAK N-terminus inserting inside the activation pocket of plasminogen and thereby modifying plasmin selectivity.^22,23^ By activating plasminogen into plasmin, *S. aureus* can degrade fibrin clots and clumps, IgG, and C3b opsonins and activate metalloproteases. These mechanisms facilitate immune escape and bacterial spreading.^15,24,25^

In this study, we considered several previously unexplored aspects of the interactions between *S. aureus* adhesins of the A region and protease components of the coagulation system. The first aspect is that during the course of human infection, *S. aureus* MSCRAMMs encounter thrombin and plasmin (Figure 1a). These are promiscuous serine proteases capable of hydrolyzing peptide bonds in a wide variety of protein/peptide substrates;^55^ however, we hypothesized that the natural role of *S. aureus* adhesins as virulence factors would render them resistant to proteolytic digestion by thrombin and plasmin. We exposed the functional binding units of the A region of various MSCRAMMs to thrombin and plasmin digestion and precisely localized the cutting sites within the overall sequence and structure (Figure 1c). One prior study showed that thrombin cuts FnBPA at a conserved site between the N1 and N2 without impacting its fibrinogen or elastin binding capacity,^56^ but otherwise the thrombin and plasmin proteolytic sensitivity of *S. aureus* adhesins was previously unknown.

The second aspect we considered was the role that SAK could play in modulating the proteolytic susceptibility of *S. aureus* adhesins of the A region. Recent computational modeling efforts allowed engineering of SAK for better thrombolytic behavior;^57^ however, its native role as a virulence factor in *S. aureus* infections has been poorly investigated. We evaluated the impact of SAK binding to plasmin on the digestion patterns observed within the adhesins. What emerged was a picture of a highly resistant A region with a limited number of cutting sites localized within specific subregions of the structure, with SAK binding leading to increased proteolytic resistance.

## Materials and Methods

### Reagents

All reagents were at least of analytical purity grade and were purchased from Sigma-Aldrich (St. Louis, MO, USA), Thermo Fisher Scientific (Waltham, MA, USA), GE Healthcare (Chicago, IL, USA) or New England Biolabs (Ipswich, MA, USA). Synthetic genes coding for the N2–N3 domains of the different adhesins and N1–N2 domains of Cna were purchased from TWIST Bioscience or from Thermo Fischer Scientific (see table). Primers for cloning were purchased from Microsynth AG (Balgach, Switzerland). Plasmin purified from human sera and recombinant human thrombin were purchased from Milan Analytica (Rheinfelden, Switzerland). Recombinant staphylokinase was purchased from Creative Enzymes (New York, USA). Buffers were filtered through a 0.2 μm poly(ether sulfone) membrane filter (Sarstedt, Nuembrecht, Germany) prior to use. The pH of all buffers was adjusted at room temperature.

### Cloning

Most constructs were obtained by inserting a synthetic gene fragment into an amplified pET28a plasmid using a Gibson assembly with homologous overhangs. The gene for ClfB was amplified from plasmid #101717 purchased from Addgene.^58^ Final plasmids allowed the expression of each adhesin with an N-terminal fibrinogen beta (Fgβ) peptide tag at the N-terminus and a HIS-YbbR tag at the C-terminus. The complete set of primary amino acid sequences of all protein constructs is given in the Supporting Information (Figure S1). SdrG was cloned in a plasmid containing only the C-terminus HIS-YbbR tags because it binds Fgβ. Sanger sequencing (Microsynth AG) confirmed the sequences of fusion proteins.

### Expression and Purification of Functional Binding Domains of *S. aureus* MSCRAMMs

Recombinant plasmids encoding N2–N3 domains (or N1–N2 domains in the case of Cna) were transformed into *E. coli* BL21 (DE3). Overnight preculture was started in 20 mL of Luria–Bertani (LB) medium with 50 μg mL^–1^ kanamycin at 37 °C. 500 mL LB culture was started with 0.1 optical density at 600 nm (OD600); 50 μg mL^–1^ kanamycin and 2 mM CaCl_2_ were also added for SdrD and SdrC constructs. The culture was placed at 37 °C and 200 rpm until an OD of ∼0.5–0.7 was reached.

For all constructs, 0.3 mM IPTG was used for induction, and cultures were then placed at 20 °C for 20 h. Cells were harvested by centrifugation at 4000*g* for 20 min at 4 °C.

All expressed recombinant proteins included a hexa-histidine (His_6_) tag for purification by immobilized metal ion affinity chromatography. The cell pellet was resuspended in TBS Ca^2+^ buffer (25 mM Tris, 72 mM NaCl, and 1 mM CaCl_2_; pH 7.2) and lysed by sonication. Cell debris was removed by centrifugation (14 000*g* for 20 min at 4 °C) and filtered (0.45 μm). The filtered supernatant was loaded onto a His-Trap FF 5 mL column (GE Healthcare) and washed with TBS Ca^2+^ buffer. Bound protein was eluted using TBS Ca^2+^ containing 250 mM imidazole buffer. Eluted protein was further purified using a Superose 6 10/300 GL size-exclusion column (GE Healthcare). Quality control was performed using SDS-PAGE, mass spectrometry, and thermal melting analysis (see below). Protein solutions for long-term storage were concentrated using a Vivaspin 6 centrifugal filter (molecular weight cutoff 10 kDa, GE Healthcare) and stored in 25% (v/v) glycerol at −20 °C.

### SDS-PAGE Analysis of Adhesin Digestion Kinetics by Plasmin

Twenty-five micromolar plasmin was mixed with 5 μM of the respective adhesin of interest in TBS Ca^2+^ buffer, corresponding to *t* = 0 min. After 3 min, 1 h, 3 h, and 6 h of incubation, a sample of the original mixture was withdrawn, gel loading solution was added, and the sample was heated at 95 °C for 3 min. Samples were loaded in 12% SDS-polyacrylamide gel (PAGE), and the gel was run at a constant voltage of 280 V. Gels were stained using SimplyBlue SafeStain (Thermo Scientific).

### LC-MS Analysis

Five micromolar plasmin or thrombin was incubated with 70 μM of the respective adhesin of interest in TBS Ca^2+^ buffer for 16 h at room temperature. When SAK was used, 5 μM of the toxin was first premixed with 5 μM of plasmin in TBS Ca^2+^ buffer for 10 min, and 70 μM of adhesin was then added. The *K*_D_ of this interaction is ∼10 nM; saturation of the binding sites should be reached.^58^ The reaction ran likewise for 16 h at room temperature. Samples were precipitated using methanol chloroform precipitation and resuspended 0.1% trifluoroacetic acid (TFA) solution so that a final concentration of 0.4 mg mL^–1^ of adhesin was obtained. One microliter of this sample was then injected at 0.3 mL min^–1^ on a Phenomenex Jupiter 5 μm C4 (50 × 2 mm) column, kept at 30 °C. HRMS-spectra were acquired on a Bruker maXis 4G ESI-QTOF (Bruker Daltonics), and data deconvolution was done with Bruker Compass DataAnalysis 4.4.

### Differential Scanning Fluorimetry (Nano DSF)

A Prometheus NT.48 instrument (NanoTemper Technologies) was used to characterize the thermal denaturation temperatures of the various adhesins. Standard capillaries were filled with 10 μL of each sample at 1 mg mL^–1^ and placed on the sample holder in triplicate. After a discovery scan that identified 50% intensity as an optimal setting, a one-step temperature gradient of 1 °C min^–1^ from 20 to 95 °C was applied, and the intrinsic protein fluorescence at 330 and 350 nm was recorded. Melting temperatures were calculated from the inflection points of the second derivative of the curves of the average of the three technical replicates.

## Results

### Characterization of Recombinant Adhesins

DNA sequences encoding the binding portions of the respective N-regions of each adhesin (Table 1) were cloned into pET28a plasmids. We included an N-terminal Fgβ tag in all constructs (with the exception of SdrG) and a C-terminal 6xHIS-YbbR tag to facilitate a variety of biophysical assays, purification, and labeling reactions. Amplification primers are provided in Table S1. All constructs were successfully expressed in *E. coli* BL21 (DE3) and purified using nickel chromatography and size exclusion. The quality of the purified products was assessed using SDS-PAGE, mass spectrometry, and thermal stability analysis (see below). The amino acid sequences of the final constructs can be found in the Supporting Information (Figure S1). In the following text, we use the terms MSCRAMMs and adhesins to refer only to the binding N-domains, corresponding to the A region, in our recombinant constructs.

To assess the quality and correct folding of the purified recombinant adhesins, we determined their respective molecular weights using liquid chromatography coupled to mass spectrometry (LC-MS) and characterized their denaturation temperatures using differential scanning fluorimetry (DSF; Table 2, Figures S2 and S3). LC-MS confirmed the expected molecular weights and indicated that the samples were sufficiently pure to carry out the intended protease digestion study. Only the construct containing the N1–N2 domains from *S. aureus* Cna showed a significant 17 Da difference between the theoretical expected mass (38 691 Da) and the mass obtained by LC-MS (38 674 Da; Table 2, Figure S3). This mass shift precisely corresponded to the loss of an ammonia group (NH_3_) and spontaneous formation of an intramolecular isopeptide bond between a Lys residue and an Asn or Gln residue in Cna. On the basis of analysis of its crystal structure and those of close homologues, an isopeptide bond was previously hypothesized in the N2 domain of *S. aureus* Cna; however, it had not been experimentally confirmed.^59^ The 71 °C melting temperature (*T*_m_) obtained for Cna was also consistent with the presence of this putative isopeptide bond, which is known to confer thermostability (Table 2, Figure S2).^60^ With similar overall fold architecture, the other adhesins in the library meanwhile exhibited *T*_m_ values between 56 and 61 °C, significantly lower than that of Cna. Other adhesins from *Streptococci* and subspecies spontaneously form isopeptide bonds, and these folds have been split and engineered into spontaneous protein ligation systems.^61,62^ Our results therefore suggest *S. aureus* Cna as a possible candidate for engineering of an orthogonal isopeptide bond system. Although these proteins are composed of two domains, a single denaturation peak event was observed by DSF for a large majority of them (Figure S2). This suggested the two DEv-IgG domains denatured together, exposing buried fluorescent amino acids in the same temperature range. Only FnBPA showed two distinct peaks at 44 and 56 °C (Figure S2), which we interpreted as indicative of two separate unfolding events, one of the domains being less stable than the other.

**Table 2:** Quality Assessment of the Purified Adhesins (Mass of the Undigested Proteins Determined by LC-MS)

adhesin	expected size (Da)	mass of undigested protein (Da)	denaturation temperature (°C)
ClfA	41 977	41 978	57
ClfB	43 914	43 914	56
FnBPA	40 801	40 800	44/56
FnBPB	41 010	41 010	48
SdrC	41 289	41 288	61
SdrD	41 035	41 035	58
SdrE	41 940	41 940	57
Cna^a^	38 691	38 674	71
SdrG	40 510	40 510	56

a Mass difference between expected and observed was consistent with an isopeptide bond.

### FnBPB N2–N3 Structure Prediction Using AlphaFold

**Figure 2:**
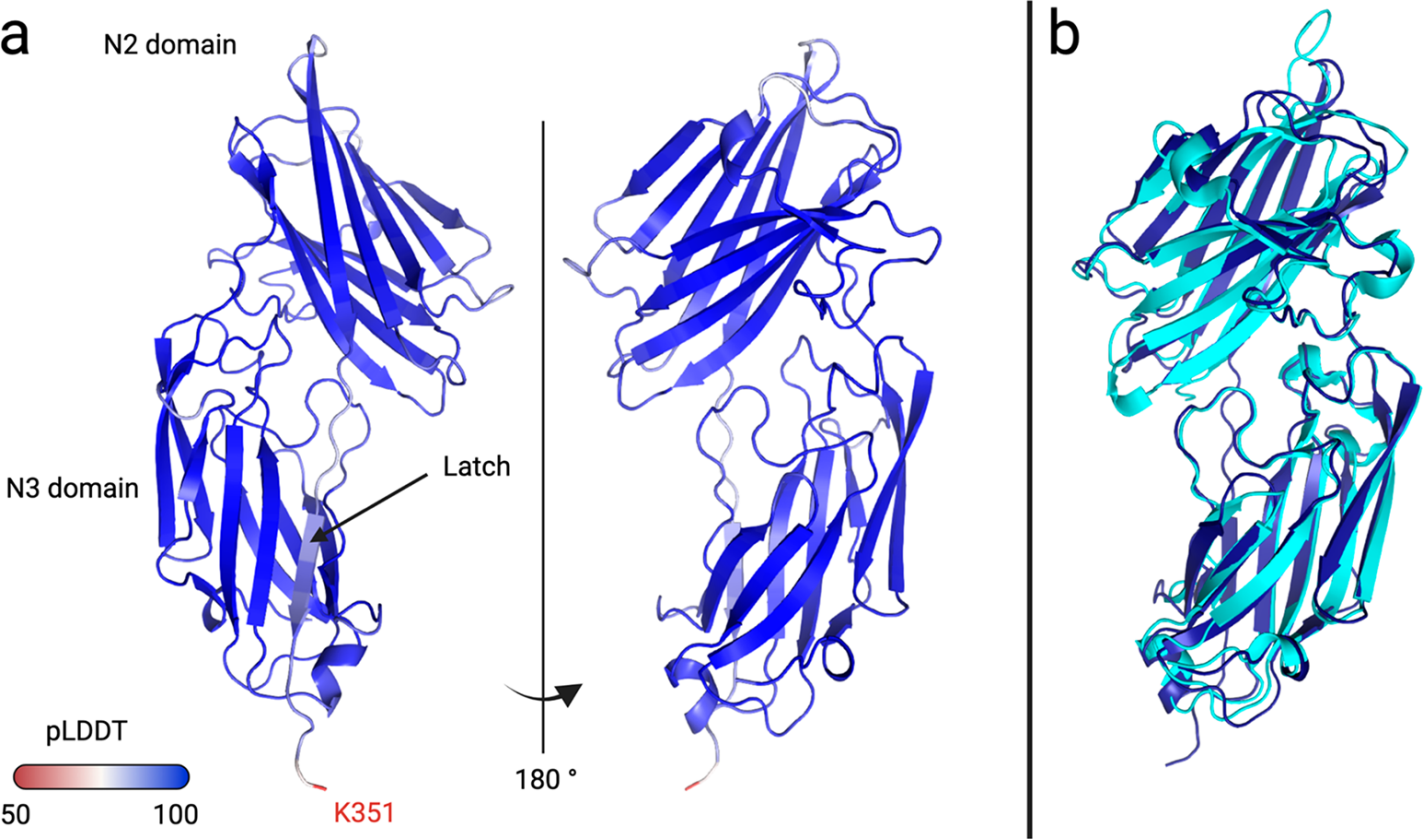
Structural model of FnBPB N2–N3 domains determined by AlphaFold. (a) The pLDDT value indicated high confidence in the prediction along the majority of the adhesin length. The minimum pLDDT was obtained for the C-terminus residue K351. (b) Superposition of FnBPA N2–N3 (light blue, PDB 4B5Z) with the predicted FnBPB N2–N3 structure (dark blue) obtained from AlphaFold.

We had no high-resolution structure for the FnBPB N2–N3 construct, so we used AlphaFold to generate a 3D structure prediction.^63^ The prediction depicts the N2 and N3 domains, each adopting the DEv-Ig fold, connected by a short linker (Figure 2a). Superposition of the model with the crystal structure of its closest homologue (FnBPA N2–N3; PDB 4B5Z) resulted in alignment of ∼1500 atoms with a final RMSD value of 0.893 Å (Figure 2b). PLDDT values reflect the confidence level in the predicted residue position and fell between 58.54 and 98.76 (Figure 2a, Figure S4). Lower confidence values were observed for linker residues, while residues located within predicted secondary structural elements showed high confidence (pLDDT > 90). Along the last β-strand (N341 to D346), the pLDDT values fell slightly to between 82.87 and 89.30 (Figure 2a, Figure S4).^63^

**Figure 3:**
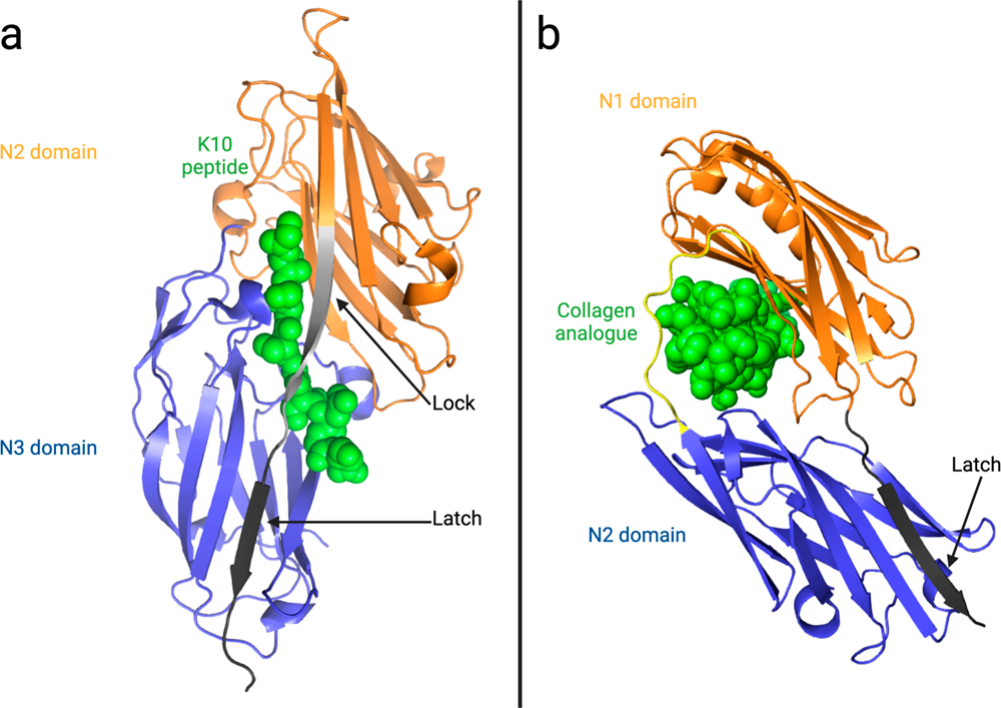
Binding mechanism of *S. aureus* MSCRAMMs. (a) High-resolution structure of the N2 (orange) and N3 (blue) domains of ClfB in complex with the K10 peptide (green spheres; PDB 4F20). The two N-domains adopt DEv-IgG folds connected by a linker. The binding pocket is a trench at the interface of N2 and N3. During assembly of the complex, the K10 peptide first docks in the trench via β-strand complementation with N3. The C-terminus of N2 then rearranges and forms interactions with the K10 peptide during the locking step of the DLL mechanism (light gray). A second β-strand complementation then occurs where the disordered C-terminus (in dark gray) of N2 folds to form a new β-strand that inserts in the N3 domain, forming the latch. (b) Cartoon representation of the hug mechanism performed by the N1 and N2 domains of Cna (blue and orange, respectively) in complex with a collagen analogue (green spheres; PDB 2F6A). The N-domains form a wrench where collagen is fixed at the interdomain interface by hydrophobic interactions. Based on structures with and without collagen, a sequential binding mechanism is also suggested. N2 first recognizes and binds collagen, which is then hugged by the adhesin through additional interactions with N1 and the linker. Last, latching consists of the C-terminal β-strand of N2 (in dark gray) inserting in the N1 β-sheet.

The MSCRAMMs studied here share a common binding mechanism, referred to as dock, lock, and latch (DLL). This mechanism was first described for the *S. epidermidis* MSCRAMM SdrG and involves β-strand complementation where the last β-strand of N3 is inserted into N2.^64,65^ The structural model of FnBPB N2–N3 obtained from AlphaFold corresponded to the latched conformation (Figure 2a). The DLL mechanism (Figure 3a) consists of sequential binding steps involving structural rearrangements. First, the partner docks in the trench formed at the interface between the two DEv-IgG folds, and next the C-terminal part of the adhesin rearranges. The flexible end forms contacts with the partner (corresponding to the lock step), until the last residues insert themselves into N2 (latch step). In the absence of a binding partner, the structure of the C-terminal portion of most adhesins could not be solved, where a few were found in the latched state.^51,66^ The flexible nature of the C-terminus of these adhesins helped explain the relative uncertainty of the position occupied by the last residues of FnBPB in the AlphaFold prediction (Figure 2a, Figure S4).

### Thrombin Digestion Profile of *S. aureus* MSCRAMMS

Thrombin, like plasmin, is a serine protease of the trypsin family capable of hydrolyzing peptide bonds immediately following Arg or Lys residues. The enzyme is somewhat promiscuous and accepts a range of peptide substrates.^67−70^ Previous work had identified a unique thrombin cutting site located between the N1 and the N2 domains of FnBPA. Here, we studied more generally how the binding regions of *S. aureus* MSCRAMMs are processed by this enzyme.^67^ Our purified recombinant adhesins were exposed to thrombin overnight, and the resulting mixture was precipitated and resuspended in a suitable solution for LC-MS analysis (Figure 1c).

After thorough analysis of the spectra (Figure S5), only one or two major peaks were observed for a majority of adhesins, corresponding to one or two protein populations with masses very close to the undigested adhesin (Table 3). In all adhesins, the N-terminus remained either completely undigested (starting at G2) or proteolyzed after R13, corresponding to cleavage of the Fgβ N-terminal affinity tag. As for the C-termini, for a majority of the proteins, the identified fragments ended at the terminal Ala of the tag, meaning that no digestion occurred. In the cases of ClfB and SdrG, digestion occurred in the C-terminal tag where the tag sequences were slightly different from those of the other recombinant proteins (Table 3, Figures S1 and S5). For example, we found peptides corresponding to G14–A393 for ClfA, G14–A372 for FnBPA, G14–A372 for FnBPB, G14–A373 for SdrC, G14–A379 for SdrD, G14–A385 for SdrE, and G14–A359 for Cna and peptides G14–R381 and G2–R333 for ClfB and SdrG, respectively (Table 3, Figure S5). We consider cleavage of the proteins at the affinity tag not to be physiologically relevant since the tags are flexible and unstructured and were introduced artificially into the N-termini and C-termini of our recombinant samples. We therefore classified core adhesins that were only cleaved at the affinity tags to be resistant to protease digestion.

**Table 3:** Identified Peptide Masses and Assignment to the Corresponding Sequence Fragments Determined by LC-MS Following Thrombin Digestion

				structural location of cutting sites	
adhesin	mass of undigested protein (Da)	mass(es) of thrombin digestion fragments (Da)	corresponding fragment	N-ter	C-ter
ClfA	41 978	40 634	G14 to A393	tag	undigested
ClfB	43 914	40 040	G14 to R381	tag	tag
FnBPA	40 800	25 155	G2 to K236	undigested	linker, N3
		33 856	G14 to K323	tag	linker, N3
		39 457	G14 to A372	tag	undigested
FnBPB	41 010	37 380	G14 to K351	tag	flexible C-ter
		39 667	G14 to A372	tag	undigested
SdrC	41 288	39 945	G14 to A373	tag	undigested
SdrD	41 035	39 692	G14 to A379	tag	undigested
SdrE	41 940	40 596	G14 to A385	tag	undigested
Cna	38 674	35 044	G14 to K338	tag	flexible C-ter
		37 331	G14 to A359	tag	undigested
SdrG	40 510	37 241	G2 to R333	undigested	tag

Only FnBPA, FnBPB, and Cna contained thrombin digestion sites located away from the disordered affinity tags. These constructs were clearly cut within the adhesin sequence and generated peaks with smaller molecular masses. Two digestion products were identified for FnBPA, G2 to K236 and G14 to K323, each corresponding to a thrombin cutting site located in a flexible loop of N3 (Figure S6). The sequence of FnBPA is highly variable among the different lineages of *S. aureus*, unlike the other adhesins,^71^ making these two thrombin cutting sites rather unconserved (Figure S7).

The only basic residue targeted by thrombin within FnBPB was K351, which was the last residue before the C-terminal tag. The AlphaFold structure prediction indicated that this residue was located in a flexible linker region (Figure 2a) connecting the N3 domain to the adjacent fibronectin binding domain in the context of the full MSCRAMM. Moreover, Lys at this position was poorly conserved among the different *S. aureus* lineages (Figure S8).

**Figure 4:**
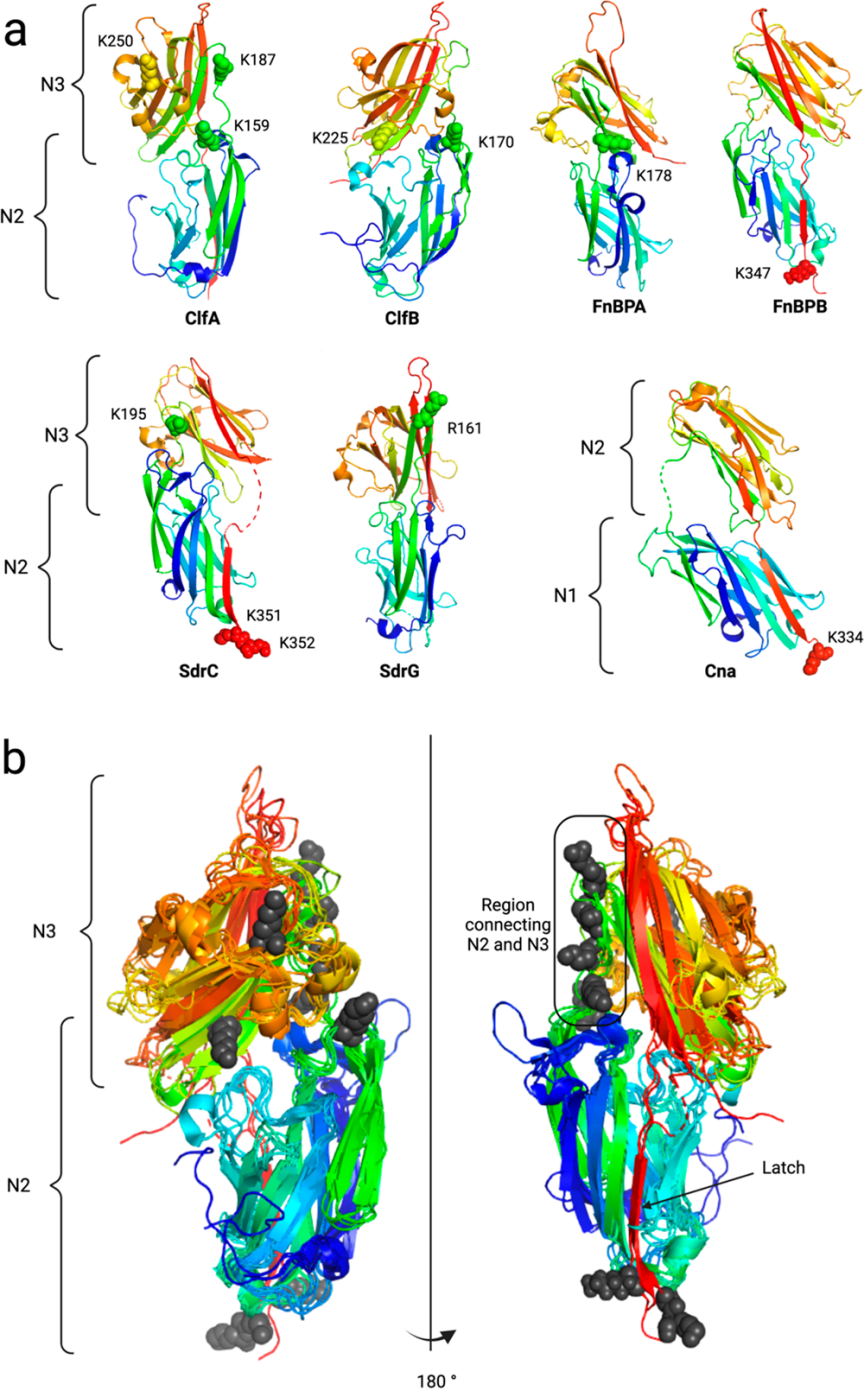
Localization of the plasmin cutting sites on MSCRAMM structures. (a) The basic residues targeted by plasmin are shown as spheres. The chains are colored as a rainbow, with the N-terminus in blue and the C-terminus in red. (b) Superposition of the N2–N3 domains of the adhesins and their plasmin cutting sites. The basic residues recognized by plasmin are shown as gray spheres. PDB codes: ClfA 5JQ6, ClfB 4F24, FnBPA 4B5Z, SdrC 6LXH, and SdrG 1R19.

A similar digestion pattern was observed for the Cna construct where a single cutting site was identified at the C-terminal end of N2. This MSCRAMM, whose structure was solved both in the presence and absence of a collagen analogue, binds through an alternative DLL mechanism called the hug mechanism (Figure 3b).^20^ Here, collagen is wrapped or hugged by the two DEv-IgG domains N1 and N2, forming a wrench-like structure. The binding is also latched by β-strand complementation where the last β-strand of N2 inserts into the N1 β-sheet. Based on the crystal structure, the identified cutting site within Cna located at K338 is highly conserved among the various *S. aureus* strains (Figure S9) and is located after the last residue involved in the latch, in the flexible end of N2 (Figures 3b and 4a). Since all the required structural elements for the DLL or the hug mechanism remained intact following thrombin digestion,^15,21^ our data demonstrate that thrombin digestion of FnBPB or Cna at the surface of *S. aureus* would in fact release the functional binding domains from the cell wall, generating a soluble virulence factor that maintains binding ability.

Aside from the cut sites identified at the affinity tags of all constructs, and the indicated sites of Cna, FnBPA, and FnBPB, the remainder of the *S. aureus* core adhesins (i.e., ClfA, ClfB, SdrC, SdrD, SdrE, SdrG) were completely resistant to thrombin digestion, and intact functional N2–N3 domains were still detectable after overnight exposure to the enzyme (Table 3, Figure S5).

### Plasmin Digestion Profile of *S. aureus* MSCRAMMS

*S. aureus* is known to bind plasminogen at its surface^67,68^ and activate both bound and unbound plasminogen into plasmin through secretion of SAK (Figure 1a).^68,69^ We examined the lytic susceptibility of the functional N-domains of the surface adhesins in the presence of plasmin using the same experimental strategy as was employed with thrombin. Purified recombinant adhesins were exposed to plasmin overnight, and the resulting mixture was prepared and analyzed by LC-MS. Table 4 provides an overview of the identified fragments.

**Table 4:** Identified Peptide Masses Determined by LC-MS after Plasmin Digestion and Assignment to the Corresponding Fragment

				structural location of cutting sites	
adhesin	mass of undigested protein (Da)	mass(es) of plasmin digestion fragments (Da)	corresponding fragment	N-ter	C-ter
ClfA	41 978	14 961	G14 to K159	tag	linker, N2
		17 845	G14 to K187	tag	linker between N2 and N3
		24 859	G14 to K250	tag	linker, N3
		40 321	G14 to S390^a^	tag	tag
ClfB	43 914	16 524	G14 to K170	tag	linker, N2
		22 364	G14 to K225	tag	β-strand, N3
		39 546	G14 to L377^a^	tag	tag
		39 674	G14 to K378	tag	tag
FnBPA	40 800	17 697	G14 to K178	tag	linker between N2 and N3
FnBPB	41 010	36 895	G14 to K347	tag	flexible C-ter
SdrC	41 288	17 773	K195 to K351	linker between N2 and N3	flexible C-ter
		17 902	K195 to K352	linker between N2 and N3	flexible C-ter
		37 531	G14 to K351	tag	flexible C-ter
		37 659	G14 to K352	tag	flexible C-ter
SdrD	41 035	39 379	G14 to S376^a^	tag	tag
		39 507	G14 to K377	tag	tag
SdrE	41 940	40 283	G14 to S382^a^	tag	tag
		40 412	G14 to K383	tag	tag
Cna	38 674	34 616	G14 to K334	tag	flexible C-ter
		35 044	G14 to K338	tag	flexible C-ter
SdrG	40 510	17 922	G2 to R161	undigested	linker between N2 and N3
		36 875	G2 to K330	undigested	tag

a Last residue of the fragment was not an Arg or a Lys, likely attributable to TAFI carboxypeptidase activity.

In several samples (i.e., ClfA, ClfB, SdrD, and SdrE), we observed peptide fragments that did not terminate with Lys or Arg at the C-terminal end as was expected for plasmin (Table 4). When analyzing the deconvoluted mass spectra (Figure S10), we found that twin peaks were present: one corresponded to a digested fragment with an unexpected C-terminal residue, and a second slightly larger fragment contained the expected C-terminal Lys residue (e.g., fragments G14 to L377 and G14 to K378 of ClfB; fragments G14 to S376 and G14 to K377 of SdrD; fragments G14 to S382 and G14 to K383 of SdrE). Only ClfA fragment G14 to S390 was not accompanied by a twin peak in the mass spectrum; however, we found that residue S390 in ClfA is indeed followed by K391. This led us to suspect that plasmin was cutting after K391, but the digestion product was further processed by exopeptidase activity, which removed the newly exposed C-terminal Lys. Literature indicated that plasmin forms interactions with the thrombin activatable fibrinolysis inhibitor (TAFI) *in vivo*(72,73) and that TAFI regulates fibrinolysis by removing C-terminal lysine residues from fibrin chains through exopeptidase activity. The human plasmin used in our experiments was purified directly from human sera; therefore, we interpreted our mass spectrometry results as indicating traces of TAFI contamination in our plasmin enzyme, which was consistent with removal of C-terminal Lys residues from the plasmin-digested MSCRAMM fragments. The difference in behavior of different peptide fragments exhibiting the absence of or partial or total removal of the C-terminal Lys could be explained by a short TAFI half-life^74,75^ or possible substrate specificity and substrate preference of TAFI.

Compared to thrombin digestion, which generated only one product in a majority of cases, plasmin led to the production of more diverse fragments (Table 4). It was indeed possible to identify large plasmin-resistant fragments in each construct where digestion occurred only in the affinity tags. For example, plasmin-resistant fragments were found for ClfA G14 to S390, ClfB G14 to K378, SdrD G14 to K377, SdrE G14 to K383, and SdrG G2 to K330. We subdivided the observed plasmin digestion susceptibility into four categories described below.

In the first category, we observed adhesins that were totally resistant to plasmin digestion. SdrD and SdrE fell into this category and we note that they were also not susceptible to thrombin digestion (Table 3). Only two identified fragments were found for each of these proteins, corresponding to fragments generated through plasmin cutting in the N-terminal Fgβ tag and in the C-terminus YbbR tag, followed by putative TAFI carboxypeptidase activity (Table 4, Figure S10) to remove terminal Lys residues.

The second category contained the adhesins FnBPB and Cna, which generated one or two large plasmin-resistant fragments, where the C-terminal cut site was located within the core domains themselves. About Cna more precisely, two of such fragments were produced: G14 to K334 and G14 to K338 (Table 4). The K338 C-terminal site was also obtained with thrombin digestion (Table 3, Figure S9), and K334 is the last residue whose structure could be solved without a collagen analogue (Figure 4a).^67,72^ This cutting site is well conserved in *S. aureus* strains (Figure S9), where the Lys is located immediately downstream of the latching strand residues (Figure 4a). Similarly, the FnBPB C-terminal cutting site at K347 represents the last residue of the plasmin-resistant fragment. According to the AlphaFold structure prediction, this Lys is in a flexible C-terminal region downstream of latching strand residues (Figure 4a). Unlike the identified thrombin cutting site located a couple residues downstream, this plasmin cutting site seemed highly conserved among different *S. aureus* strains (Figure S8), which was surprising since FnBPA and FnBPB N2–N3 regions are the most variable among the characterized MSCRAMMs.^71,76^ These findings implied that the functional binding N1–N2 domains of Cna and the N2–N3 domains of FnBPB can be cut and released from the *S. aureus* surface by plasmin and thrombin.

The third category included the adhesins ClfA, ClfB, SdrC, and SdrG where plasmin digestion produced two to four fragments (Table 4). Among the various digestion products, a large fragment close to the full-length protein could be found: G14 to S390 for ClfA, G14 to K378 for ClfB, G14 to K352 for SdrC, and G2 to K330 for SdrG. Since LC-MS is not the best method to evaluate the relative abundance of the different fragments, we performed SDS-PAGE to analyze digestion kinetics and estimate the abundance of the fragments (Figure S11). Purified adhesins were mixed with a 5-fold excess of plasmin. Aliquots of this initial reaction were taken at various time points. The aliquots were treated by adding gel loading solution and heating at 95 °C for 3 min to stop the reaction. After 6 h, all aliquots were loaded on SDS-gel. After migration and staining, we could observe the formation of digestion products and visually determine which fragments were the most abundant. For these four adhesins (ClfA, ClfB, SdrC, and SdrG), a high molecular weight band was still clearly visible after 6 h, with some additional smaller populations slowly appearing. The molecular masses of the largest populations were in agreement with the size of the largest digestion fragments identified by LC-MS (Table 4, Figures S10 and S11). We could determine that for these four adhesins, the large digestion products were generated by cuts away from the adhesin core itself (Table 4) and were highly resistant to plasmin.

Other fragments found in the third category showed different C-terminal cut sites: G14 to K159, G14 to K187, and G14 to K250 for ClfA; G14 to K170 and G14 to K225 for ClfB; and G2 to R161 for SdrG (Table 4). These C-terminal cut sites were localized to flexible linkers, both in the N2 and N3 domains and in the linker joining the N2 and N3 (Figure 4a,b). Based on the SDS-gel kinetic analysis, it was unfortunately not possible for us to determine whether the various digestion reactions occurred sequentially or stochastically in parallel. The three internal cutting sites in ClfA were well conserved even if not present in every strain (Figure S12), while the two ClfB cut sites were extremely conserved (Figure S13). Interestingly, ClfA K159 and ClfB K170 were located at the exact same structural location within the N2 domain (Figure 4a,b), even though the two cutting sites were divergent in terms of sequence: NVKK|TG for ClfA and KAPK|SG for ClfB.

Unlike the other adhesins, SdrC showed digested populations whose N-terminus was not in the tag but in the adhesin itself (Table 4), located in the linker connecting the two DEv-IgG fold domains (Figure 4). Surprisingly, we could only detect the fragments corresponding to the full N3 domain (K195 to K351 and K195 to K352) but not the fragment corresponding to the N2 domain, suggesting that after cleavage from N3, the N2 domain was further processed and degraded and that in the context of the full protein, N3 shields N2 from plasmin digestion. All SdrC fragments contained either K351 or K352 at the C-terminus. These Lys residues located immediately downstream of the latching strand (Figure 4a) were highly conserved (Figure S14).^72^ Our data therefore suggest that plasmin digestion of SdrC would lead to the release of either the full N2–N3 region or the N3 domain alone from the *S. aureus* surface.

A fourth category of digestion pattern was observed for FnBPA, where only one 18 kDa fragment was detected after overnight digestion. This fragment corresponded to plasmin cutting sites located in the N-terminal tag and in the linker connecting N2 and N3 domains (Table 4, Figure 4a,b). The entire N2 domain of FnBPA was resistant to plasmin, while N3 was most likely totally digested (opposite the case of SdrC, where N3 was retained by N2 was digested). Since the thermal denaturation analysis by DSF showed two temperature ranges for FnBPA where secondary structures were lost (Figure S1), a current hypothesis is that FnBPA N3 is more prone to proteolysis and less thermostable than N2. The first peak observed by DSF, at 44 °C, would correspond to N3 unfolding when the second peak at 56 °C would reflect N2 unfolding. As mentioned before, FnBPA shows poor sequence identity within the different lineages of the pathogen, making this site not present in every *S. aureus* strain (Figure S7).

We next structurally superimposed the N2–N3 domains and their plasmin cutting sites and observed that most of the cutting sites are located in N3 (Figure 4b). Only ClfA and ClfB had a shared cutting site in N2. Moreover, the sites in N3 were all on the same side of the molecule, where the trench is formed at the interface of the DEv-IgG folds. Half of the cutting sites were concentrated in the region connecting N2 and N3 with ClfA, FnBPA, SdrC, and SdrG all displaying this structural cutting site. The connecting region is made of a dozen residues and comprises a very short β-strand, but in some cases this latter strand is missing. Here the cut happened before, after, or within this small β-strand. A final hot spot we identified for plasmin cutting was the C-terminal end of these proteins, at sites found downstream of the latching strand (Figure 4a,b).

### SAK Interaction with Plasmin Enhances Resistance to Digestion

SAK interacts with the serine protease domain of plasmin with nanomolar affinity but not directly with residues of the active site. Doing so, it provokes structural changes, creating new subsites and changing the enzyme selectivity and specificity.^73^ We tested whether the interaction between SAK and plasmin would have an impact on the digestion pattern of the MSCRAMMs. A first control experiment consisted of mixing SAK and plasmin together overnight in the absence of any MSCRAMM substrate, analyzing digestion patterns by LC-MS and comparing them to the undigested masses (Figure S15). Full length undigested SAK had a molecular mass of 15 564 Da by LC-MS while after mixture with plasmin, its mass decreased to 14 336 Da. This corresponded to cutting after Lys 11, as previously reported (Figure S15).^77^ This plasmin digestion site in SAK is required for SAK activation.^23,77^ The SAK/plasmin digestion experiment on recombinant MSCRAMMs then consisted of premixing equimolar concentrations of SAK and plasmin for 10 min prior to addition of the respective MSCRAMM, followed by an overnight digestion reaction and analysis by LC-MS.

A larger number of total fragments were detected for SAK/plasmin digestion than for plasmin alone. We found that for several of the MSCRAMMs, two related fragments were detected with mass differences of ∼1343–1344 Da (Table 5). These twin fragment populations had the same C-terminal ends but different N-termini. One population started at G14, like a majority of the fragments generated by plasmin alone, and the other population started at G2, indicating that no cutting occurred in the Fgβ N-terminal tag. This pattern was observed for ClfA (G2 to S390 and G14 to S390; G2 to A393 and G14 to A393), FnBPA (G2 to Y322 and G14 to Y322; G2 to K323 and G14 to K323), SdrC (G2 to K352 and G14 to K352), SdrD (G2 to A379 and G14 to A379), and SdrE (G2 to S382 and G14 to S382; G2 to K383 and G14 to K383; G2 to A385 and G14 to A385). This suggested that the plasmin/SAK complex was less able to recognize and cut the flexible N-terminal tag of these constructs as compared with plasmin alone. We furthermore observed in the plasmin/SAK digested constructs putative TAFI activity that produced twin peaks for several of the identified fragments. This led to the identification of two extra populations for SdrE, for instance, G2 to S382 and G14 to S382 along with twin fragments G2 to K383 and G14 to K383 were generated by SAK/plasmin digestion.

**Table 5:** Identified Peptide Masses Determined by LC-MS after Plasmin/SAK Digestion and Assignment of the Corresponding Fragment

				structural location of cutting sites	
adhesin	mass of undigested protein (Da)	mass(es) of plasmin/SAK digestion fragments (Da)	corresponding fragment	N-ter	C-ter
ClfA	41 978	40 321	G14 to S390^a^	tag	tag
		40 634	G14 to A393	tag	undigested
		41 664	G2 to S390^a^	undigested	tag
		41 977	G2 to A393	undigested	undigested
ClfB	43 914	40 040	G14 to R381	tag	tag
		41 385	G2 to R383	undigested	tag
FnBPA	40 800	33 727	G14 to Y322^a^	tag	linker, N3
		33 855	G14 to K323	tag	linker, N3
		35 071	G2 to Y322^a^	undigested	linker, N3
		35 199	G2 to K323	undigested	linker, N3
FnBPB	41 010	36 895	G14 to K347^b^	tag	flexible C-ter
		37 139	G14 to K349	tag	flexible C-ter
		37 251	G14 to L350^a^	tag	flexible C-ter
		37 380	G14 to K351	tag	flexible C-ter
SdrC	41 288	37 530	G14 to K351^b^	tag	flexible C-ter
		37 658	G14 to K352^b^	tag	flexible C-ter
		39 002	G2 to K352^b^	undigested	flexible C-ter
SdrD	41 035	39 691	G14 to A379	tag	undigested
		41 034	G2 to A379	undigested	undigested
SdrE	41 940	25 098	Q155 to S382^a^	linker, N2	tag
		25 277	Q155 to K383	linker, N2	tag
		25 411	Q155 to A385	linker, N2	undigested
		40 282	G14 to S382^a^	tag	tag
		40 412	G14 to K383	tag	tag
		40 596	G14 to A385	tag	undigested
		41 617	G2 to S382^a^	undigested	tag
		41 755	G2 to K383	undigested	tag
		41 939	G2 to A385	undigested	undigested
Cna	38 674	35 044	G14 to K338^b^	tag	flexible C-ter
SdrG	40 510	37 240	G2 to R333	undigested	tag

a The last residue of the fragment was not an Arg or a Lys, likely attributable to TAFI carboxypeptidase activity.

b The C-terminal cutting site was the same as when the adhesin was exposed to plasmin alone.

The most important trend was that SAK interaction with plasmin either did not change the outcome of the digestion or it provided the adhesin with additional resistance to digestion. SdrD was still totally resistant, but the formation of the complex between plasmin and SAK led to different results. After overnight digestion, the full recombinant protein was still identified: fragments G2 to A379 and G14 to A379 were found. This meant that K377, in the YbbR tag, was no longer recognized as a substrate. However, this was not universally true for all constructs since K383 of the Fgβ-SdrE-HIS-YbbR construct, occupying the same position in the tag, was still recognized and cut by the plasmin/SAK complex.

**Figure 5:**
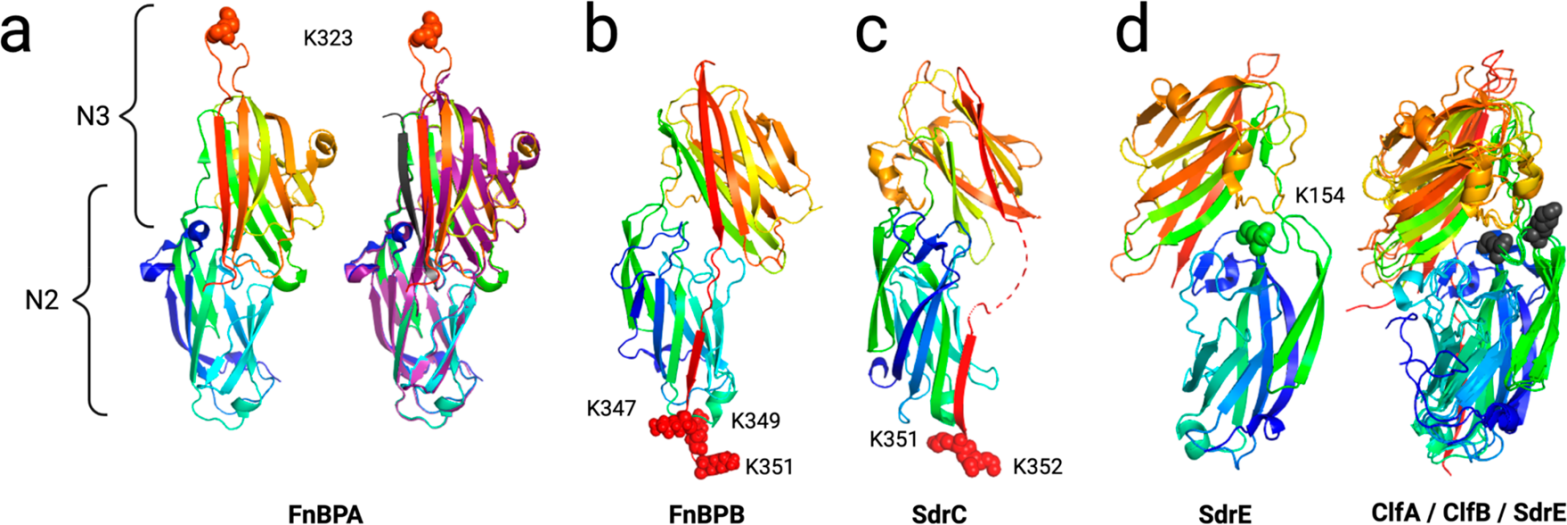
Localization of the plasmin/SAK cutting sites. Basic residues cleaved by the plasmin/SAK complex are shown as spheres. The chains are colored as a rainbow, with the N-terminus in blue and the C-terminus red. (A, left) FnBPA crystal structure in the absence of its binding partner. (Right) Superposition of FnBPA without (rainbow colors) and with (purple) the bound Fgβ peptide (dark gray). B and C show the plasmin/SAK cutting sites in FnBPB and SdrC, respectively. (D, left) SdrE crystal structure and localization of the single cutting site introduced upon SAK binding to plasmin. (Right) Superposition of the N2–N3 domains of the ClfA, ClfB, and SdrE. ClfA K159 and ClfB K170 plasmin cutting sites are shown along with SdrE K154, targeted by the plasmin/SAK complex. These three sites are in the same loop, connecting the last two β-strands of the N2 domain. PDB files: FnBPA 4B5Z and 4B60, SdrC 6LXH, SdrE 5WTA, ClfA 5JQ6, ClfB 4F24.

The outcome of Cna and FnBPB digestions also remained unchanged. No cutting occurred after K334 (Table 4) at the C-terminus of the Cna N2 domain, but the cleavage site after K338 was still recognized, making the sequence around this Lys the only one recognized by thrombin, plasmin, and the plasmin/SAK complex (Tables 3, 4, and 5 and Figure S9). Regarding FnBPB, the three Lys residues following the latching strand were found to be cutting sites for SAK/plasmin complexes: K347, K349, and K351 (Table 5, Figure 5B). K347 was already recognized by plasmin alone and K351 by thrombin (Tables 3 and 4, Figure S8).

For some adhesins, SAK binding to plasmin offered a protection to digestion. ClfA, ClfB, SdrC, and SdrG harbored plasmin cutting sites located in flexible loops of the N2 and/or N3 domains: ClfA K159, K187, and K250; ClfB K170 and K225; and SdrC K195 and SdrG R161 (Table 4). When plasmin was complexed with SAK, these cuts were no longer detected. In the case of ClfA, the cutting sites in the tags remained the same, while for ClfB and SdrG there were new sites in the C-terminus tag (Table 4 and Table 5). A peak corresponding to the undigested protein was detected for ClfA: G2 to A393 (Table 5). For SdrC, only the C-termini cutting sites after K351 and K352 remained (Figure 5C).

FnBPA gave mixed results, with some new sites being recognized by plasmin/SAK and other susceptible sites being protected. Larger fragments were identified which started at G2 or G14 and ended at K323 or Y322. The linker between N2 and N3 was no longer digested (Table 4, Figure 4a). The sequence recognized by the complex and located in the N3 subdomain was already identified as a thrombin target (Figures S6 and S7). The newly recognized K323 site was situated within a flexible linker connecting the last two β-strands of N3 (Figure 5A).^78^ FnBPA binds Fgγ using a variation of the DLL mechanism. In the absence of a latching strand, it binds its partner as efficiently. Fgγ forms an additional strand, parallel to the *G*′ strand in N3 analogous to a zipper interaction (Figure 5A).^78^ The plasmin/SAK digestion occurring after K323 would therefore likely prevent any interaction with Fgγ.

As for SdrE, a peak corresponding to the undigested adhesin was still detected; however, a new digestion site could be identified, after K154, when this adhesin was totally resistant to both plasmin and thrombin (Table 4, Table 5, Figure 5D). This unique cutting site was in a loop connecting the last two β-strands of the N2 domain (Figure 5C). However, the sequence of this cutting site is not strictly conserved in different strains, and variation of the residues downstream of the Lys residue was found (Figure S17), indicating that this digestion site may only be relevant for some *S. aureus* strains. Nonetheless, we noticed that the same loop was targeted by plasmin alone in both ClfA and ClfB (Figures 4b, 5d).

### DEv-IgG Fold Resistance

SdrG, an MSCRAMM from the surface of *S. epidermidis*, also showed a strong resistance toward blood protease digestion, despite the fact that this *Staphylococcus* strain is coagulase negative and does not hijack the host coagulation system like *S. aureus*.^79^ The recognition site on SdrG for plasmin was a site in the region connecting the N2 and the N3 domains (Figure 4a,b). This was in line with what was observed for *S. aureus* adhesins: overall, two weak spots susceptible to proteolysis were identified and located outside of the folded N domains. The first site was the region connecting the two DEv-IgG folds. Interestingly, in the many crystal structures solved for these proteins, the two functional N domains are oriented in a specific way one to another, due to large contact surfaces.^6,26,34,49,64,66,78,80^ The degree of freedom of the linker joining the N domains is therefore relatively restrained, while other linker regions containing protease-susceptible basic residues and joining the intradomain β-strands are more accessible. Yet the frequency of digestion in these interdomain loops was very low. The second weak spot was at the C-terminus, downstream of the latching strand. Unfortunately, we cannot properly evaluate the relevance of this site based on the results of the present study because the N2 or N3 domains of our recombinant proteins were followed by a short flexible linker, a HIS tag, and the YbbR tag, introducing a bias. In the *WT* proteins, the functional binding domains of the A region are followed by other domains (Figure 1a,b), possibly providing steric hindrance and blocking access of thrombin, plasmin, and/or plasmin/SAK complexes.

The *S. aureus* adhesins exhibit low sequence similarity (Figure S18), so we attributed the general and shared proteolytic resistance to a property of the structures adopted by the N domains. The crystal structure of ClfB (PDB 4F24) was used for a search on the DALI server that looks for close structural homologues. Many good hits were found, with *Z* scores >10 and RMSD < 5 Å. Among them were other *S. aureus* MSCRAMMs: Bbp (PDB 5CFA), SdrD (PDB 4JDZ), FnBPA (PDB 4B5Z), SdrC (PDB 6LEB), and ClfA (PDB 1N67), but also MSCRAMMs from other *Staphylococcus* species, for example, UafA from *S. saprophyticus* (PDB 3IRP), and from different pathogenic bacteria like ACE from *Enterococcus faecalis* (PDB 5CFA), Srr1 and Srr2 from *Streptococcus agalactiae* (PDB 4MBO and 4MBR, respectively). We also found structural homologues in MSCRAMMs from different organisms like ALS3, found at the surface of the pathogenic yeast *Candida albicans* (PDB 4LEE). Based on this, it appears that the DEv-IgG fold was selected by pathogenic organisms for many reasons. Not only are they capable of binding different partners, but they also provide extreme mechanical stability to these interactions.^58^*S. aureus* MSCRAMMs are able to sustain interactions up to 2 nN pulling force, the equivalent of a covalent bond.^52^ Moreover, in this work, we provide evidence that this fold is also resistant to trypsin-like enzymatic digestion.

## Discussion

A special feature of *S. aureus* is its ability to hijack the human coagulation cascade by activating thrombin and plasmin, two rather unspecific and promiscuous serine proteases. In the present work, we evaluated how *S. aureus* surface proteins are processed by these enzymes and whether or not SAK, a virulence factor that interacts with and activates plasmin, could influence the cutting site specificity. We focused on the N-terminal N domains for eight *S. aureus* adhesins along with SdrG from *S. epidermidis*, which all share a common Dev-Ig fold and binding mechanism. We did not address the protease susceptibility of the downstream B domains or fibronectin binding domains, which are part of the full length mature adhesins *in vivo* (Figure 1a). We found that after overnight exposure to thrombin, plasmin, or the plasmin/SAK complex, large protease-resistant fragments of the MSCRAMM binding regions were detected.

We found that in most cases, following thrombin digestion, only one population corresponding to hydrolysis of the peptide backbone at the N-terminus and C-terminus affinity tags could be detected. These flexible affinity tags included in our recombinant proteins are not considered physiologically relevant. Only three adhesins (Table 3) possessed one or two additional cutting sites at the C-terminal end. More diverse patterns were observed for plasmin digestion (Table 4, Figure 4), where half the adhesins exhibited complete protease resistance, while the other half contained digestion sites after the last β-strand (Table 4). The locations of these cutting sites meant that *in vivo* the full functional binding regions would be released into the blood containing all required elements for the DLL or the hug binding mechanism (Figure 3).

Cna N1–N2 domains and FnBPB N2–N3 domains have many characterized binding partners (Table 1). They both promote bacterial arrest on various substrates and facilitate escape from the immune system. The liberation of these binding domains into the bloodstream would mean the loss of their capacity to arrest the pathogen on ECM proteins, but since these proteins are highly functionally redundant, this role could be taken over by other MSCRAMMs. However, their release from the membrane would not prevent their immune escape activity (Table 1). Indeed, Cna N1–N2 domains would still bind complement factor C1q^45^ and in doing so would prevent C1q from interacting with C1r, leading to an early inhibition of the classical complement.^76^ FnBPB N2–N3 domains would still bind histone H3,^77^ preventing this protein from directly interacting with the pathogen’s membrane lipoteichoic acid, avoiding bleb formation and cell rupture.^81^ As for SdrC, to our knowledge, its sole characterized binding partner is β-neurexin, a protein expressed at the surface of neuronal cells (Table 1).^48^ Barbu and colleagues demonstrated that some strains of *S. aureus* are able to release a fragment of SdrC larger than just N2 and N3 in that stationary phase, and this could partially explain some rare cases of reversible acute tetraplegia associated with *S. aureus* infection. Plasmin-mediated release of N2–N3 from the SdrC polyprotein may therefore also play a role in this pathological process.

A spatially concentrated grouping of plasmin digestion sites was observed in the linker region between the two DEv-IgG fold subdomains of the proteins (Figure 4). The consequences of the linker digestion *in vivo* would include the release of binding domains from the *S. aureus* cell wall and retainment of a new N-terminal fragment on the digested MSCRAMM. When SAK interacted with plasmin, many of the internal cutting sites that were identified with plasmin alone were no longer susceptible to proteolytic digestion by plasmin (Tables 4 and 5). The modification of plasmin specificity through interaction with SAK therefore led to increased proteolytic resistance of the adhesins, suggesting conformational changes to the structure of the active site upon SAK binding. To the best of our knowledge, this is the first study reporting that SAK interaction with plasmin can modify plasmin specificity. In addition to SAK, clinical isolates of *S. aureus* also secrete coagulase and von-Willebrand factor binding protein, which bind and activate the crucial blood protease (pro)thrombin. In future studies, we imagine MSCRAMM susceptibility to thrombin digestion in the presence of coagulase and/or von-Willebrand binding protein could provide further insights.

SdrD was noteworthy as an adhesin that was highly resistant toward digestion (Tables 3, 4, and 5) and yet has no known blood borne binding partners. Its only known partner is the keratinocyte surface protein desmoglein 1,^49,50^ promoting *S. aureus* nasal colonization (Table 1).^48^ Recent work showed that the expression of SdrD improved survival of *Lactococcus lactis* in human blood, possibly by inducing killing of neutrophils, implying that SdrD does have one or several more binding partners in blood to be identified.^82^

Another noteworthy adhesin that we identified was SdrE, which binds complement factor H (Table 1).^49,50^ It was shown that this interaction allows *S. aureus* to mimic host cells and promotes immune evasion. In our data sets, SdrE was found to be totally resistant to both thrombin and plasmin. Moreover, this adhesin was the only one which for the plasmin/SAK complex induced a new cutting site within the N2 domain, although this protein was immune to plasmin digestion (Tables 4 and 5). This site was colocalized with the plasmin cutting sites found in structural homologues ClfA and ClfB (Figure 5) located in two β-strands upstream of the linker connecting the two DEv-IgG folds. These digestions would also leave the full N3 domain at the N-terminus of the MSCRAMMs.

This work therefore highlights a novel mode of interaction between *S. aureus* and protease components of the coagulation system. It was previously known that *S. aureus* activates plasmin and thrombin and secretes virulence factors including SAK. We show here that the binding domains of adhesive *S. aureus* MSCRAMMs are highly resistant to digestion by plasmin and thrombin. Based on the sequence divergence of these adhesins but the conservation of protease susceptible sites within their structures, it seems that structural aspects of the DEv-IgG fold common to these proteins and highly spread among human pathogenic organisms provide resistance toward undesired proteolysis. Further investigation into other pathogen surface proteins sharing this architecture will shed light on this virulence mechanism.

## Supporting Information Available

Biochemical and biophysical experimental results and sequence analysis (PDF)
