# Supplementary material for: Adhesive Virulence Factors of Staphylococcus aureus Resist Digestion by Coagulation Proteases Thrombin and Plasmin
Source: ACS Bio Med Chem Au. 2022 Sep 2;2(6):586–99. doi: 10.1021/acsbiomedchemau.2c00042 (PMC9782320; doi:10.1021/acsbiomedchemau.2c00042)
Supplement: Supplementary file 1 — bg2c00042_si_001.pdf [file bg2c00042_si_001.pdf]

Supplemental Information

Adhesive Virulence Factors of *Staphylococcus aureus* Resist Digestion by Coagulation Proteases Thrombin and Plasmin

Fanny Risser[1,2], Joanan López-Morales[1,2], Michael A. Nash[1,2,*]

[1] Institute of Physical Chemistry, Department of Chemistry, University of Basel, 4058 Basel, Switzerland
[2] Department of Biosystems Sciences and Engineering, ETH Zurich, 4058 Basel, Switzerland
*Correspondence to: michael.nash@unibas.ch

Construct	Strain	Primer type	Oligonucleotide sequence (5'-3') to amplify pET28a bakbone	Oligonucleotide sequence (5'-3') to amplify adhesin DNA sequence
pET28a-Fgβ-ClfA-HIS-YbbR	NCTC 8325	f	aaattgaaccaattccagagagcggtcatcatcatcaccat	x
		r	accggtgcatctgcagctacaccgctacctgtacctgc	x
pET28a-Fgβ-ClfB-HIS-YbbR	N315	f	tcaagcttcctaggtctaggagcggtcatcatcatcaccat	gcgcaggtacaggtagcggtatgggtaccccggttgttaatgcag
		r	ttaacaaccggggtacccataccgctacctgtacctgcg	tggtgatgatgatgaccgctcctagacctaggaagcttgagctc
pET28a-Fgβ-FnBPA-HIS-YbbR	NCTC 8325	f	cgaacggaaatgagaaaaatagcggtcatcatcatcacca	x
		r	ttacttgttacatctgtaccaccgctacctgtacctgcgct	x
pET28a-Fgβ-FnBPB-HIS-YbbR	NCTC 8325	f	acggcaaagataaactaaagagcggtcatcatcatcacca	x
		r	ttatttgttacatctgtaccaccgctacctgtacctgcgct	x
pET28a-Fgβ-SdrC-HIS-YbbR	NCTC 8325	f	ctaatggcgaccaaaagaaaagcggtcatcatcatcaccat	x
		r	tttgttccttgttgtggagcaccgctacctgtacctgc	x
pET28a-Fgβ-SdrD-HIS-YbbR	NCTC 8325	f	gtggcggagctggtcaagaaagcggtcatcatcatcaccatcatggtagcgac	x
		r	aaatcattaacatttttagcaccgctacctgtacctgc	x
pET28a-Fgβ-SdrE-HIS-YbbR	Mu50	f	gtactgttaaacctgaataaagcggtcatcatcatcaccatcatggtagc	x
		r	tttacattatttgaagcaacaccgctacctgtacctgc	x
pET28a-Fgβ-Cna-HIS-YbbR	NCTC 6131	f	ctgtaaaaggtgaattaaaaagcggtcatcatcatcaccat	x
		r	gttgatgaaatatctcgtgcaccgctacctgtacctgc	x

Table S1. Primers used to generate the constructs.
f: forward primer
r: reverse primer

The plasmids were all generated by Gibbson assembly between a pET28a plasmid coding for Fgβ and HIS-YbbR tags. The DNA sequence coding for the proteins also contained suitable overhanging ends in 5' and 3'. The pET28a-Fgβ-ClfB-HIS-YbbR was also generated by Gibbson assembly but starting from a plasmid containing the ClfB N2-N3 domains DNA sequence already at hand.

Fgβ-ClfA-HIS-YbbR

```
        10         20         30         40         50         60
MGTNEEGFFF SARGHRPLDG SGSGSGSAGT GSGVAADAPV AGTDITNQLT NVTVGIDSGT
        70         80         90        100        110        120
TVYPHQAGYV KLNYGFSVPN SAVKGDTFKI TVPKELNLNG VTSTAKVPPI MAGDQVLANG
       130        140        150        160        170        180
VIDSDGNVIY TFTDYVNTKD DVKATLTMPA YIDPENVKKT GNVTLATGIG STTANKTVLV
       190        200        210        220        230        240
DYEKYGKFYN LSIKGTIDQI DKTNNTYRQT IYVNPSGDNV IAPVLTGNLK PNTDSNALID
       250        260        270        280        290        300
QQNTSIKVYK VDNAADLSES YFVNPENFED VTNSVNITFP NPNQYKVEFN TPDDQITTPY
       310        320        330        340        350        360
IVVVNGHIDP NSKGDLALRS TLYGYNSNII WRSMSWDNEV AFNNGSGSGD GIDKPVVPEQ
       370                      380                              390
PDEPGEIEPI PESGHHHHHH GSDSLEFIAS KLA
```

Fgβ-ClfB-HIS-YbbR

```
        10         20         30         40         50         60
MGTNEEGFFF SARGHRPLDG SGSGSGSAGT GSGMGTPVVN AADAKGTNVN DKVTASNFKL
        70         80         90        100        110        120
EKTTFDPNQS GNTFMAANFT VTDKVKSGDY FTAKLPDSLT GNGDVDYSNS NNTMPIADIK
       130        140        150        160        170        180
STNGDVVAKA TYDILTKTYT FVFTDYVNNK ENINGQFSLP LFTDRAKAPK SGTYDANINI
       190        200        210        220        230        240
ADEMFNNKIT YNYSSPIAGI DKPNGANISS QIIGVDTASG QNTYKQTVFV NPKQRVLGNT
       250        260        270        280        290        300
WVYIKGYQDK IEESSGKVSA TDTKLRIFEV NDTSKLSDSY YADPNDSNLK EVTDQFKNRI
       310        320        330        340        350        360
YYEHPNVASI KFGDITKTYV VLVEGHYDNT GKNLKTQVIQ ENVDPVTNRD YSIFGWNNEN
       370                   380                   390             400
VVRYGGGSAD GDSAVELKLP RSRSGHHHHH HGSDSLEFIA SKLA
```

Fgβ-FnBPA-HIS-YbbR

```
        10         20         30         40         50         60
MGTNEEGFFF SARGHRPLDG SGSGSGSAGT GSGGTDVTSK VTVEIGSIEG HNNTNKVEPH
        70         80         90        100        110        120
AGQRAVLKYK LKFENGLHQG DYFDFTLSNN VNTHGVSTAR KVPEIKNGSV VMATGEVLEG
       130        140        150        160        170        180
GKIRYTFTND IEDKVDVTAE LEINLFIDPK TVQTNGNQTI TSTLNEEQTS KELDVKYKDG
       190        200        210        220        230        240
IGNYYANLNG SIETFNKANN RFSHVAFIKP NNGKTTSVTV TGTLMKGSNQ NGNQPKVRIF
       250        260        270        280        290        300
EYLGNNEDIA KSVYANTTDT SKFKEVTSNM SGNLNLQNNG SYSLNIENLD KTYVVHYDGE
       310        320        330        340        350        360
YLNGTDEVDF RTQMVGHPEQ LYKYYYDRGY TLTWDNGLVL YSNKANGNEK NSGHHHHHHG
       370
SDSLEFIASK LA
```

Fgβ-FnBPB-HIS-YbbR

```
        10         20         30         40         50         60
MGTNEEGFFF SARGHRPLDG SGSGSGSAGT GSGGTDVTNK VEVEEGSEIV GHKQDTNVVN
        70         80         90        100        110        120
PHNAERVTLK YKWKFGEGIK AGDYFDFTLS DNVETHGIST LRKVPEIKST DGQVMATGEI
       130        140        150        160        170        180
IGERKVRYTF KEYVQEKKDL TAELSLNLFI DPTTVTQKGN QNVEVKLGET TVSKIFNIQY
       190        200        210        220        230        240
LGGVRDNWGV TANGRIDTLN KVDGKFSHFA YMKPNNQSLS SVTVTGQVTK GNKPGVNNPT
       250        260        270        280        290        300
VKVYKHIGSD DLAESVYAKL DDVSKFEDVT DNMSLDFDTN GGYSLNFNNL DQSKNYVIKY
       310        320        330        340        350        360
EGYYDSNASN LEFQTHLFGY YNYYYTSNLT WKNGVAFYSN NAQGDGKDKL KSGHHHHHHG
       370
SDSLEFIASK LA
```

Fgβ-SdrC-HIS-YbbR

```
        10         20         30         40         50         60
MGTNEEGFFF SARGHRPLDG SGSGSGSAGT GSGAPQQGTN VNDKVHFSNI DIAIDKGHVN
        70         80         90        100        110        120
QTTGKTEFWA TSSDVLKLKA NYTIDDSVKE GDTFTFKYGQ YFRPGSVRLP SQTQNLYNAQ
       130        140        150        160        170        180
GNIIAKGIYD STTNTTTYTF TNYVDQYTNV RGSFEQVAFA KRKNATTDKT AYKMEVTLGN
       190        200        210        220        230        240
DTYSEEIIVD YGNKKAQPLI SSTNYINNED LSRNMTAYVN QPKNTYTKQT FVTNLTGYKF
       250        260        270        280        290        300
NPNAKNFKIY EVTDQNQFVD SFTPDTSKLK DVTDQFDVIY SNDNKTATVD LMKGQTSSNK
       310        320        330        340        350        360
QYIIQQVAYP DNSSTDNGKI DYTLDTDKTK YSWSNSYSNV NGSSTANGDQ KKSGHHHHHH
       370
GSDSLEFIAS KLA
```

Fgβ-SdrD-HIS-YbbR

```
        10         20         30         40         50         60
MGTNEEGFFF SARGHRPLDG SGSGSGSAGT GSGAKNVNDL ITSNTTLTVV DADKNNKIVP
        70         80         90        100        110        120
AQDYLSLKSQ ITVDDKVKSG DYFTIKYSDT VQVYGLNPED IKNIGDIKDP NNGETIATAK
       130        140        150        160        170        180
HDTANNLITY TFTDYVDRFN SVQMGINYSI YMDADTIPVS KNDVEFNVTI GNTTTKTTAN
       190        200        210        220        230        240
IQYPDYVVNE KNSIGSAFTE TVSHVGNKEN PGYYKQTIYV NPSENSLTNA KLKVQAYHSS
       250        260        270        280        290        300
YPNNIGQINK DVTDIKIYQV PKGYTLNKGY DVNTKELTDV TNQYLQKITY GDNNSAVIDF
       310        320        330        340        350        360
GNADSAYVVM VNTKFQYTNS ESPTLVQMAT LSSTGNKSVS TGNALGFTNN QSGGAGQESG
       370
HHHHHHGSDS LEFIASKLA
```

Fgβ-SdrE-HIS-YbbR

```
        10         20         30         40         50         60
MGTNEEGFFF SARGHRPLDG SGSGSGSAGT GSGVASNNVN DLITVTKQTI KVGDGKDNVA
        70         80         90        100        110        120
AAHDGKDIEY DTEFTIDNKV KKGDTMTINY DKNVIPSDLT DKNDPIDITD PSGEVIAKGT
       130        140        150        160        170        180
```

```
FDKATKQITY TFTDYVDKYE DIKARLTLYS YIDKQAVPNE TSLNLTFATA GKETSQNVSV
       190        200        210        220        230        240
DYQDPMVHGD SNIQSIFTKL DENKQTIEQQ IYVNPLKKTA TNTKVDIAGS QVDDYGNIKL
       250        260        270        280        290        300
GNGSTIIDQN TEIKVYKVNP NQQLPQSNRI YDFSQYEDVT SQFDNKKSFS NNVATLDFGD
       310        320        330        340        350        360
INSAYIIKVV SKYTPTSDGE LDIAQGTSMR TTDKYGYYNY AGYSNFIVTS NDTGGGDGTV
       370                                                     380
KPEGSGHHHH HHGSDSLEFI ASKLA
```

Fgβ-Cna-HIS-YbbR

```
        10         20         30         40         50         60
MGTNEEGFFF SARGHRPLDG SGSGSGSAGT GSGARDISST NVTDLTVSPS KIEDGGKTTV
        70         80         90        100        110        120
KMTFDDKNGK IQNGDMIKVA WPTSGTVKIE GYSKTVPLTV KGEQVGQAVI TPDGATITFN
       130        140        150        160        170        180
DKVEKLSDVS GFAEFEVQGR NLTQTNTSDD KVATITSGNK STNVTVHKSE AGTSSVFYYK
       190        200        210        220        230        240
TGDMLPEDTT HVRWFLNINN EKSYVSKDIT IKDQIQGGQQ LDLSTLNINV TGTHSNYYSG
       250        260        270        280        290        300
QSAITDFEKA FPGSKITVDN TKNTIDVTIP QGYGSYNSFS INYKTKITNE QQKEFVNNSQ
       310              320            330           340         350
AWYQEHGKEE VNGKSFNHTV HNINANAGIE GTVKGELKSG HHHHHHGSDS LEFIASKLA
```

SdrG-HIS-YbbR

```
        10         20         30         40         50         60
MGTEQGSNVN HLIKVTDQSI TEGYDDSDGI IKAHDAENLI YDVTFEVDDK VKSGDTMTVN
        70         80         90        100        110        120
IDKNTVPSDL TDSFAIPKIK DNSGEIIATG TYDNTNKQIT YTFTDYVDKY ENIKAHLKLT
       130        140        150        160        170        180
SYIDKSKVPN NNTKLDVEYK TALSSVNKTI TVEYQKPNEN RTANLQSMFT NIDTKNHTVE
       190        200        210        220        230        240
QTIYINPLRY SAKETNVNIS GNGDEGSTII DDSTIIKVYK VGDNQNLPDS NRIYDYSEYE
       250        260        270        280        290        300
DVTNDDYAQL GNNNDVNINF GNIDSPYIIK VISKYDPNKD DYTTIQQTVT MQTTINEYTG
       310        320        330        340        350        360
EFRTASYDNT IAFSTSSGQG QGDLPPEELK LPRSRHHHHH HGSLEVLFQG PDSLEFIASK
LA
```

Figure S1. Amino acid sequence of the purified recombinant proteins
The sequences of the N2-N3 domains are highlighted in red.

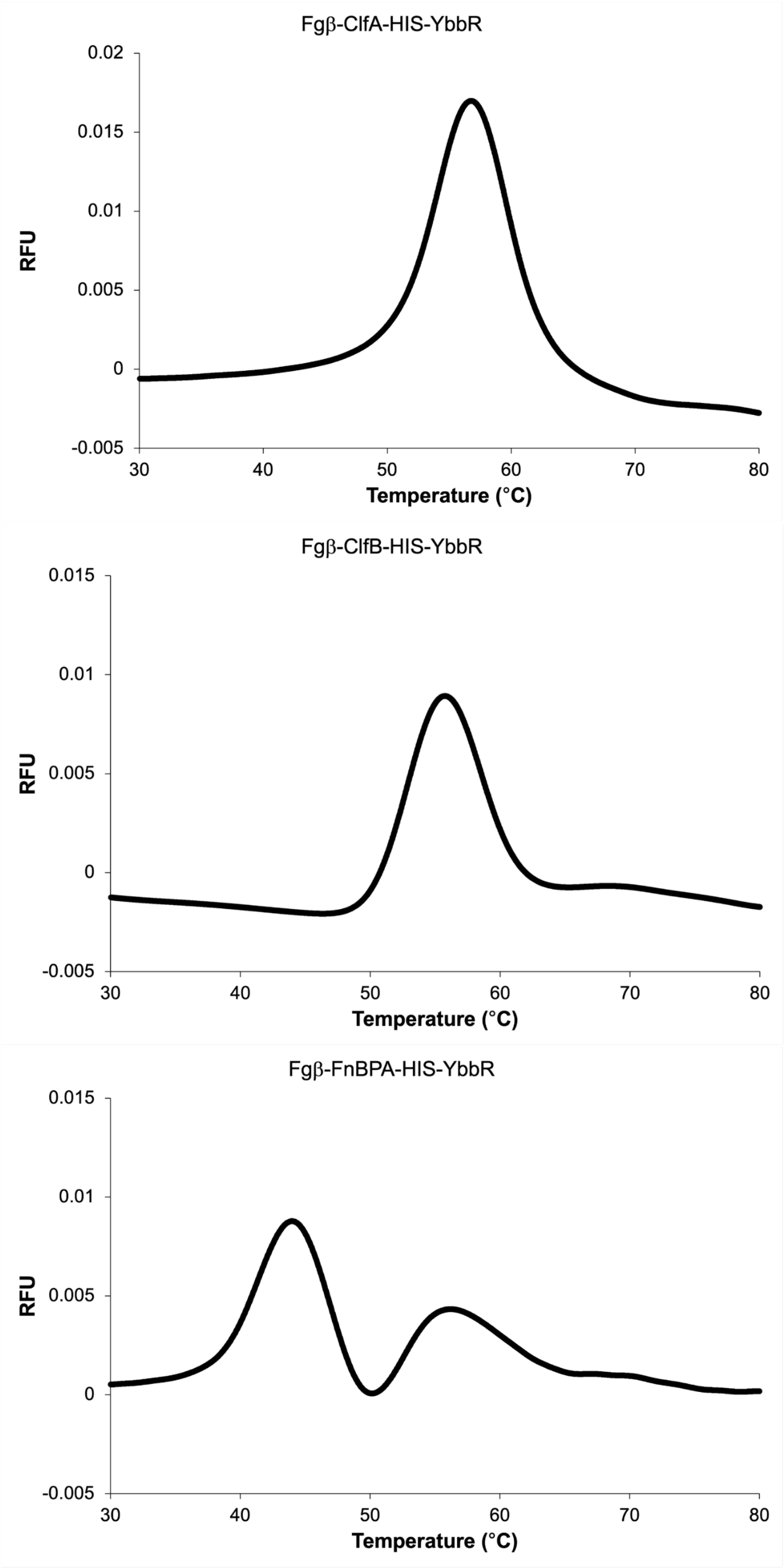

Fgβ-ClfA-HIS-YbbR
0.02
0.015
0.01
0.005
0
-0.005
RFU
30
40
50
60
70
80
Temperature (°C)
Fgβ-ClfB-HIS-YbbR
0.015
0.01
0.005
0
-0.005
RFU
30
40
50
60
70
80
Temperature (°C)
Fgβ-FnBPA-HIS-YbbR
0.015
0.01
0.005
0
-0.005
RFU
30
40
50
60
70
80
Temperature (°C)

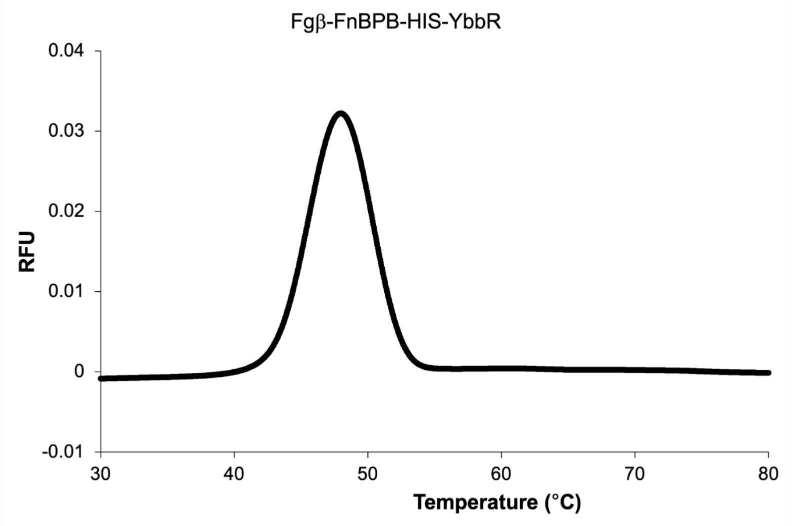
Fgβ-FnBPB-HIS-YbbR
0.04
0.03
0.02
0.01
0
-0.01
RFU
30
40
50
60
70
80
Temperature (°C)

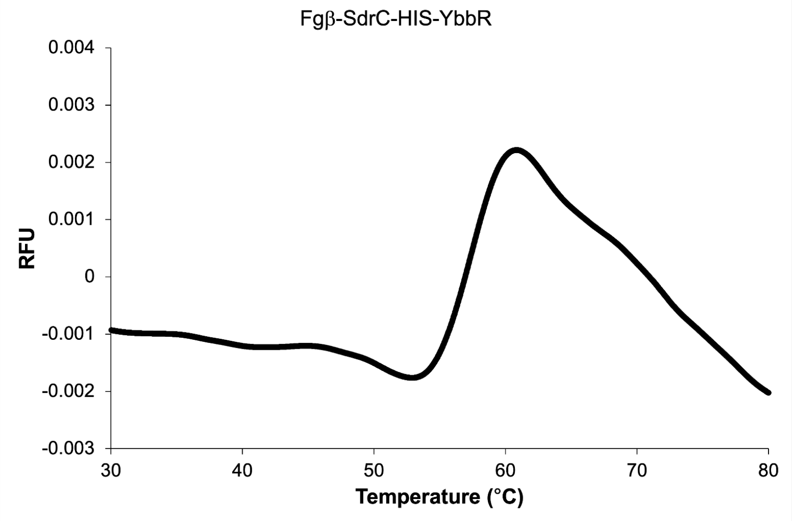
Fgβ-SdrC-HIS-YbbR
0.004
0.003
0.002
0.001
0
-0.001
-0.002
-0.003
RFU
30
40
50
60
70
80
Temperature (°C)

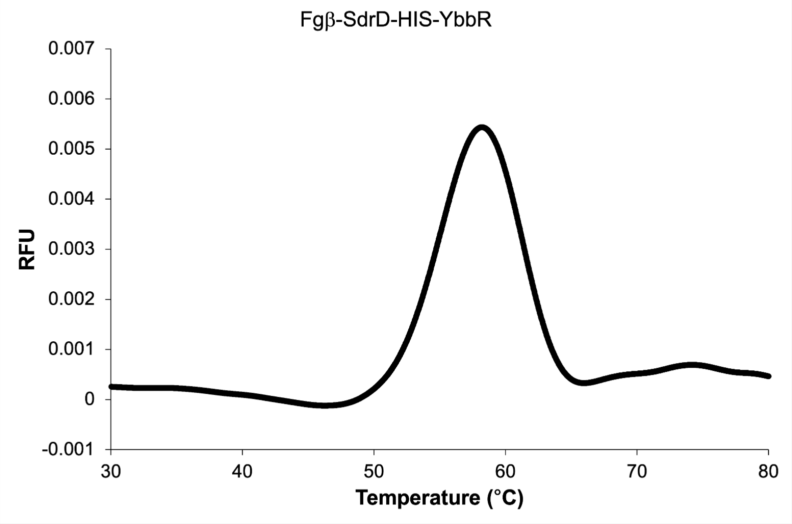
Fgβ-SdrD-HIS-YbbR
0.007
0.006
0.005
0.004
0.003
0.002
0.001
0
-0.001
RFU
30
40
50
60
70
80
Temperature (°C)

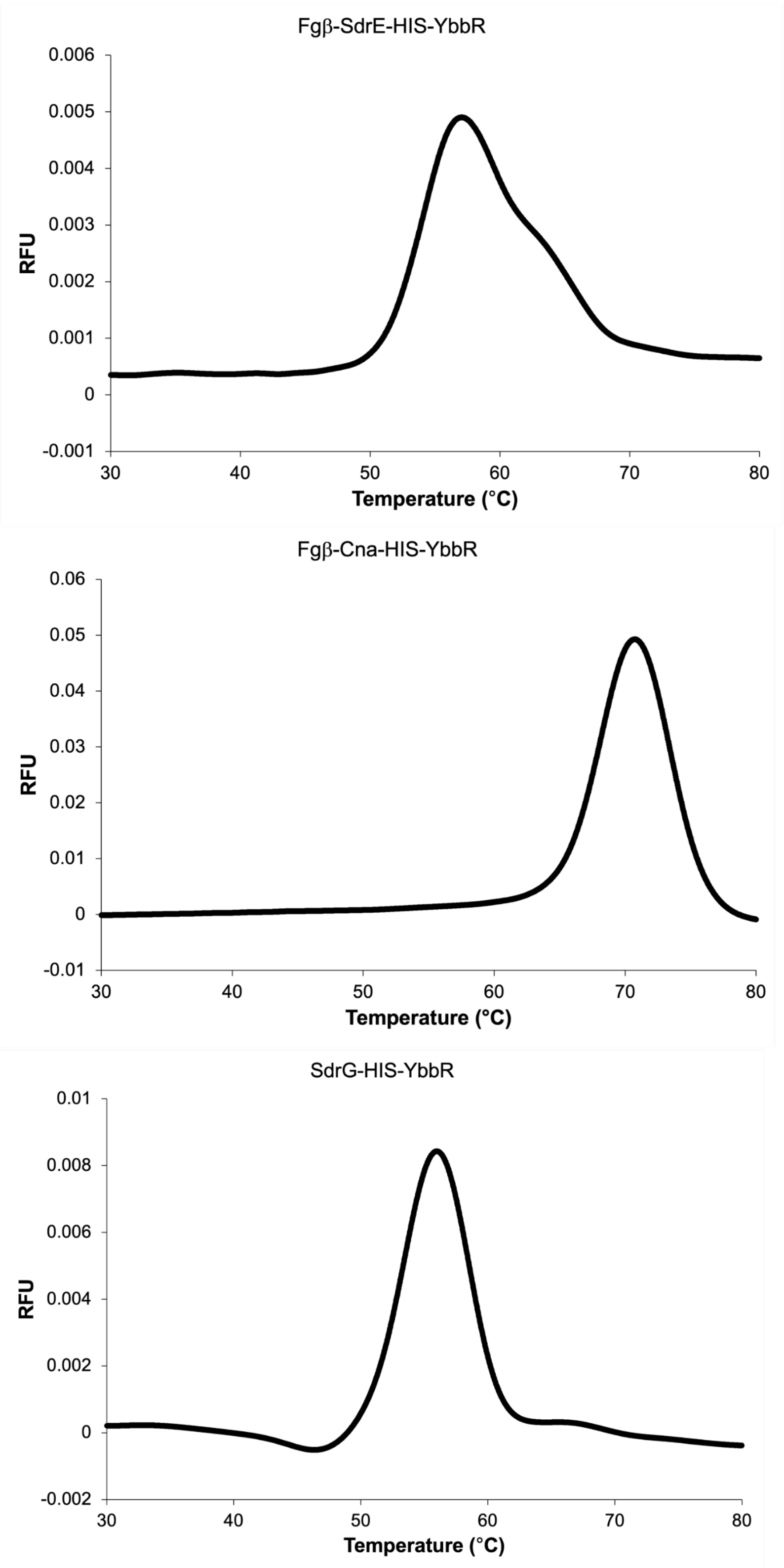

Figure S2. DSF data
The melting temperature is calculated by the first derivative of the F330/350 recorded plots.

Adhesin	Mass(es) of thrombin digestion fragments (Da)	Corresponding fragment	
		Construct numbering	Adhesin numbering
ClfA	40,634	G14 to A393	G-20 to A+21
ClfB	40,040	G14 to R381	G-23 to R+6
FnBPA	25,155	G2 to K236	G-32 to K396
	33,856	G14 to K323	G-20 to K483
	39,457	G14 to A372	G-20 to A+21
FnBPB	37,380	G14 to K351	G-20 to K480
	39,667	G14 to A372	G-20 to A+21
SdrC	39,945	G14 to A373	G-20 to A+21
SdrD	39,692	G14 to A379	G-20 to A+21
SdrE	40,596	G14 to A385	G-20 to A+22
Cna	35,044	G14 to K338	G-20 to K334
	37,331	G14 to A359	G-20 to A+21
SdrG	37,241	G2 to R333	G-2 to R+6

Adhesin	Mass(es) of plasmin digestion fragments (Da)	Corresponding fragment	
		Construct numbering	Adhesin numbering
ClfA	14,961	G14 to K159	G-20 to K346
	17,845	G14 to K187	G-20 to K374
	24,859	G14 to K250	G-20 to K436
	40,321	G14 to S390	G-20 to S+18#
ClfB	16,524	G14 to K170	G-23 to K336
	22,364	G14 to K225	G-23 to K391
	39,546	G14 to L377	G-23 to L+2
	39,674	G14 to K378	G-23 to K+3
FnBPA	17,697	G14 to K178	G-20 to K338
FnBPB	36,895	G14 to K347	G-20 to K476
SdrC	17,773	K195 to K351	K339 to K495
	17,902	K195 to K352	K339 to K496
	37,531	G14 to K351	G-20 to K495

	37,659	G14 to K352	G-20 to K496
SdrD	39,379	G14 to S376	G-20 to S+18
	39,507	G14 to K377	G-20 to K+19
SdrE	40,283	G14 to S382	G-20 to S+19
	40,412	G14 to K383	G-20 to K+20
Cna	34,616	G14 to K334	G-20 to K330
	35,044	G14 to K338	G-20 to K334
SdrG	17,922	G2 to R161	G-2 to R431
	36,875	G2 to K330	G-2 to K+3

**Adhesin**	**Mass(es) of plasmin/SAK complex digestion fragments (Da)**	**Corresponding fragment**	
		Construct numbering	Adhesin numbering
**ClfA**	40,321	G14 to S390	G-20 to S+18
	40,634	G14 to A393	G-20 to A+21
	41,664	G2 to S390	G-32 to S+18
	41,977	G2 to A393	G-32 to A+21
**ClfB**	40,040	G14 to R381	G-23 to R+6
	41,385	G2 to R383	G-35 to R+8
**FnBPA**	33,727	G14 to Y322	G-20 to Y482
	33,855	G14 to K323	G-20 to K483
	35,071	G2 to Y322	G-32 to Y482
	35,199	G2 to K323	G-32 to K483
**FnBPB**	36,895	G14 to K347	G-20 to K476
	37,139	G14 to K349	G-20 to K478
	37,251	G14 to L350	G-20 L479
	37,380	G14 to K351	G-20 to K480
**SdrC**	37,530	G14 to K351	G-20 to K495
	37,658	G14 to K352	G-20 to K496
	39,002	G2 to K352	G-32 to K496
**SdrD**	39,691	G14 to A379	G-20 to A+21
	41,034	G2 to A379	G-32 A+21
**SdrE**	25,098	Q155 to S382	Q391 to S+19
	25,277	Q155 to K383	Q391 to K+20

	25,411	Q155 to A385	Q391 to A+22
	40,282	G14 to S382	G-20 to S+19
	40,412	G14 to K383	G-20 to K+20
	40,596	G14 to A385	G-20 to A+22
	41,617	G2 to S382	G-32 to S+19
	41,755	G2 to K383	G-32 to K+2
	41,939	G2 to A385	G-32 to A+22
**Cna**	35,044	G14 to K338	G-20 to K334
**SdrG**	37,240	G2 to R333	G-2 to R+6

Table S2. Identified fragments after enzyme digestion. Both numbering systems are shown here, the first one refers to the amino acid position in the recombinant protein, starting at Met1. The second one is showing the amino acid number within the whole MSCRAMM, the amino acids in the N-terminus tag are shown as negative and the ones in the C-terminus tags as positive relative to the residues of the N2-N3 domains.

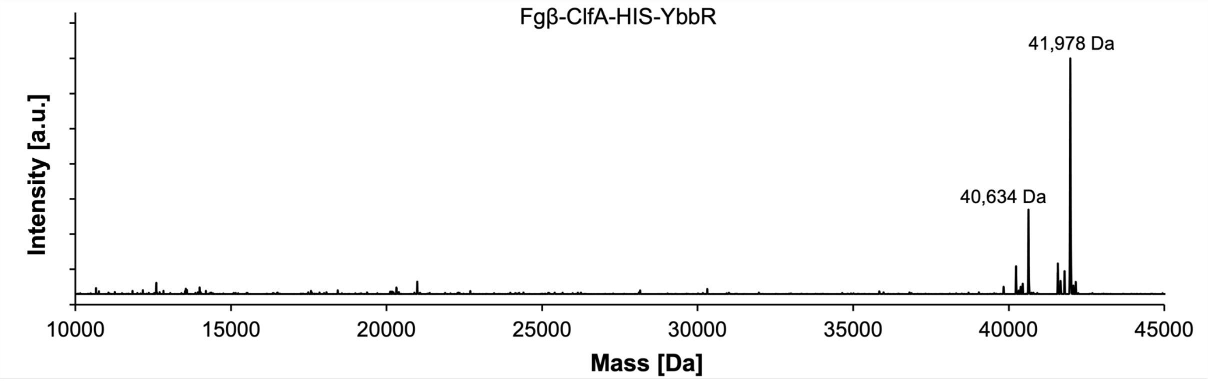

Fgβ-ClfA-HIS-YbbR
41,978 Da
40,634 Da
Intensity [a.u.]
10000
15000
20000
25000
30000
35000
40000
45000
Mass [Da]

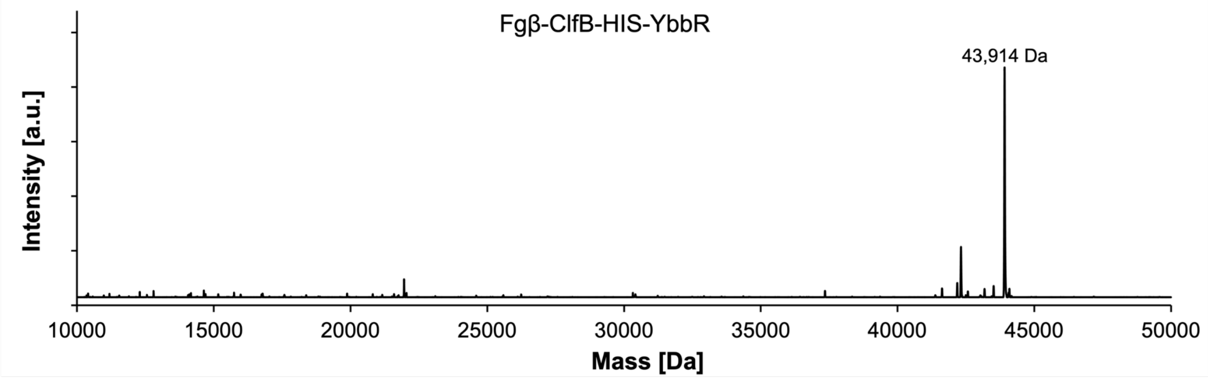

Fgβ-ClfB-HIS-YbbR
43,914 Da
Intensity [a.u.]
10000
15000
20000
25000
30000
35000
40000
45000
50000
Mass [Da]

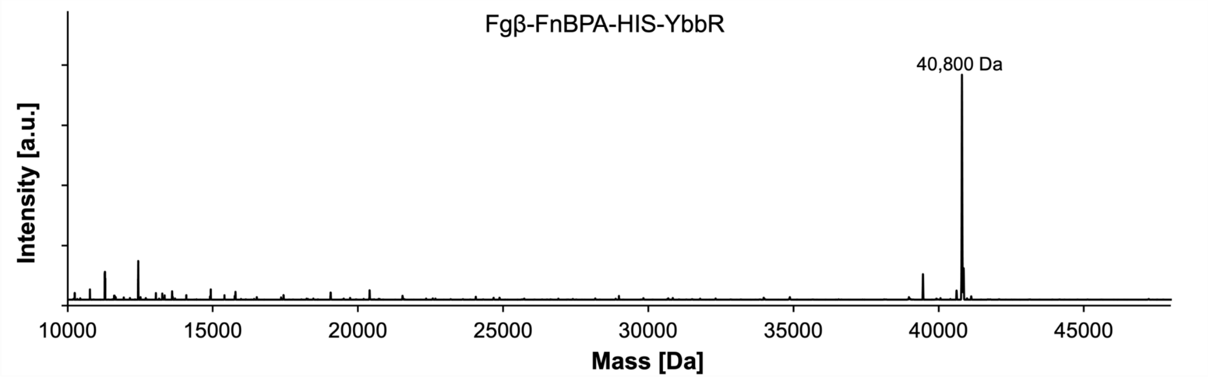

Fgβ-FnBPA-HIS-YbbR
40,800 Da
Intensity [a.u.]
10000
15000
20000
25000
30000
35000
40000
45000
Mass [Da]

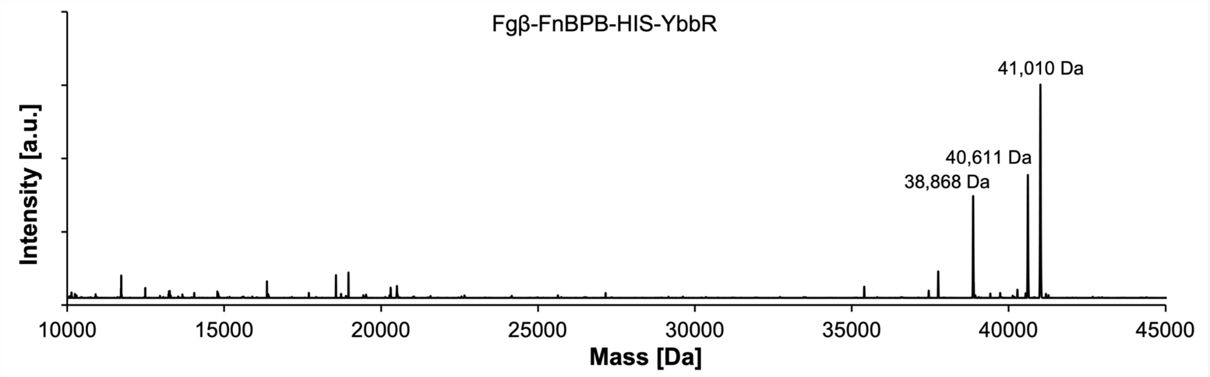

Fgβ-FnBPB-HIS-YbbR
41,010 Da
40,611 Da
38,868 Da
Intensity [a.u.]
10000
15000
20000
25000
30000
35000
40000
45000
Mass [Da]

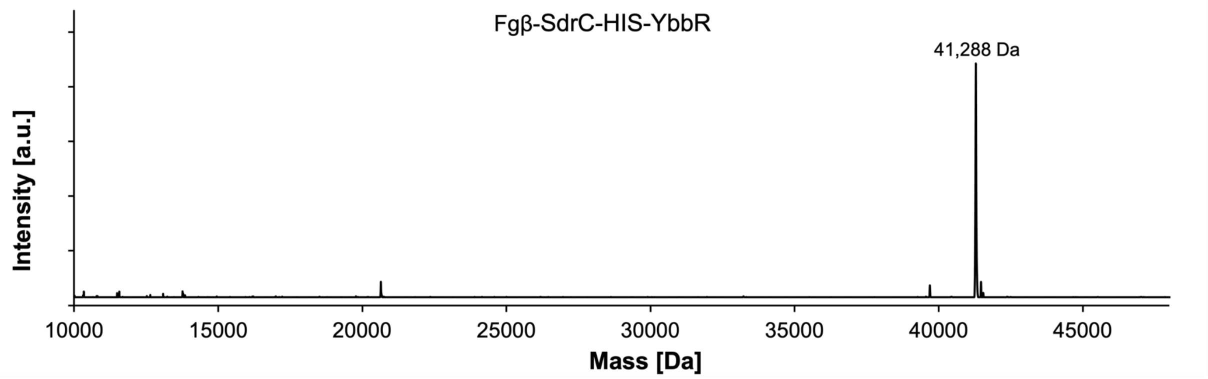
Fgβ-SdrC-HIS-YbbR
41,288 Da
Intensity [a.u.]
10000
15000
20000
25000
30000
35000
40000
45000
Mass [Da]

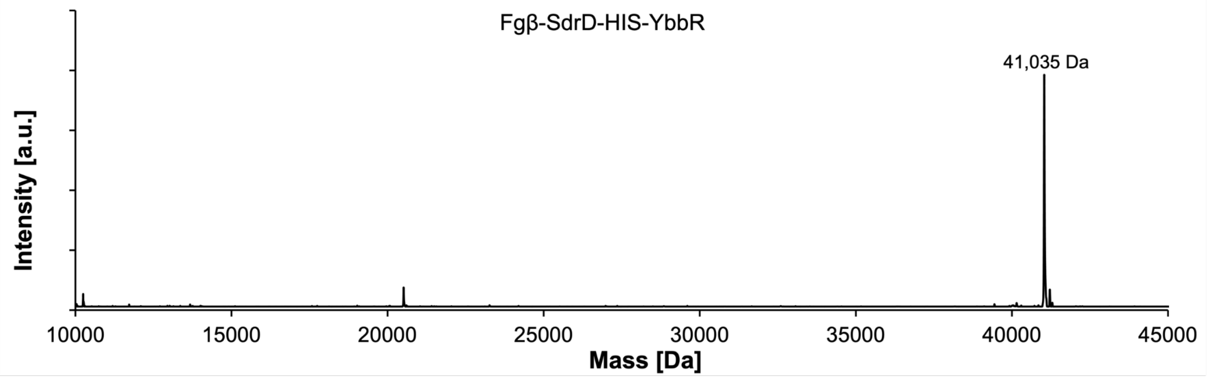
Fgβ-SdrD-HIS-YbbR
41,035 Da
Intensity [a.u.]
10000
15000
20000
25000
30000
35000
40000
45000
Mass [Da]

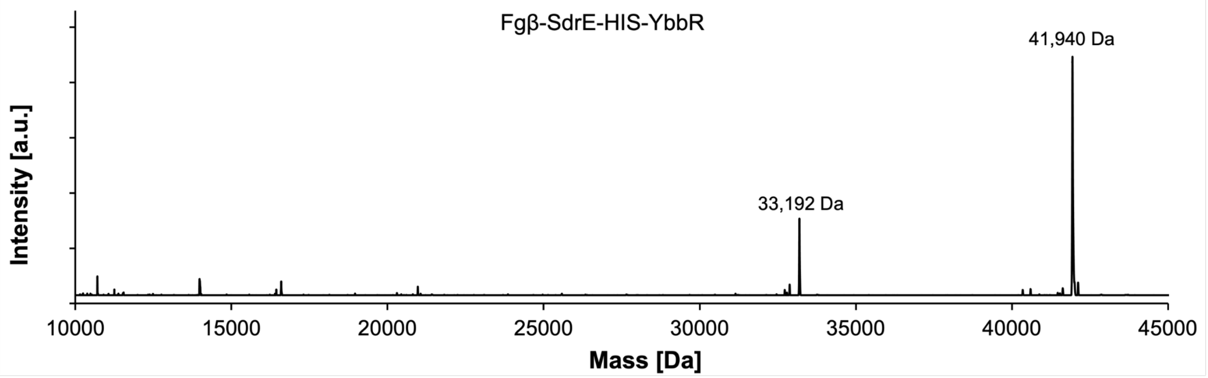
Fgβ-SdrE-HIS-YbbR
41,940 Da
33,192 Da
Intensity [a.u.]
10000
15000
20000
25000
30000
35000
40000
45000
Mass [Da]

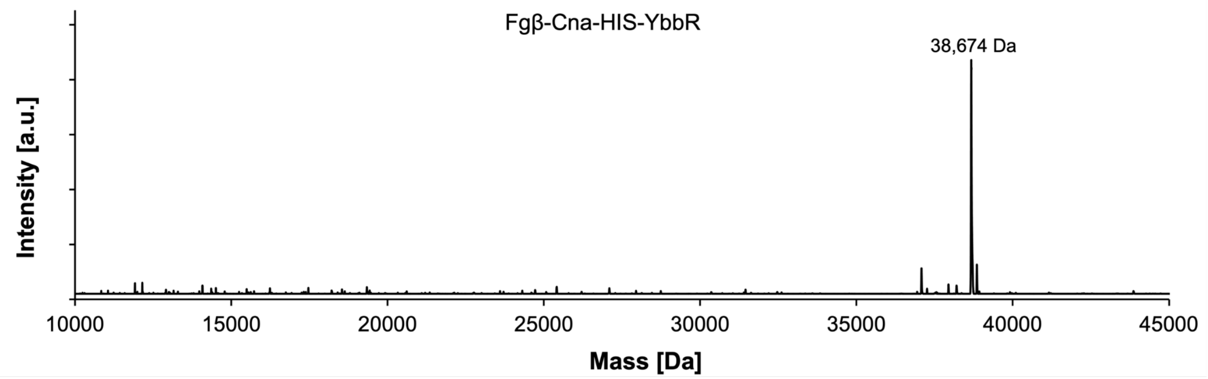
Fgβ-Cna-HIS-YbbR
38,674 Da
Intensity [a.u.]
10000
15000
20000
25000
30000
35000
40000
45000
Mass [Da]

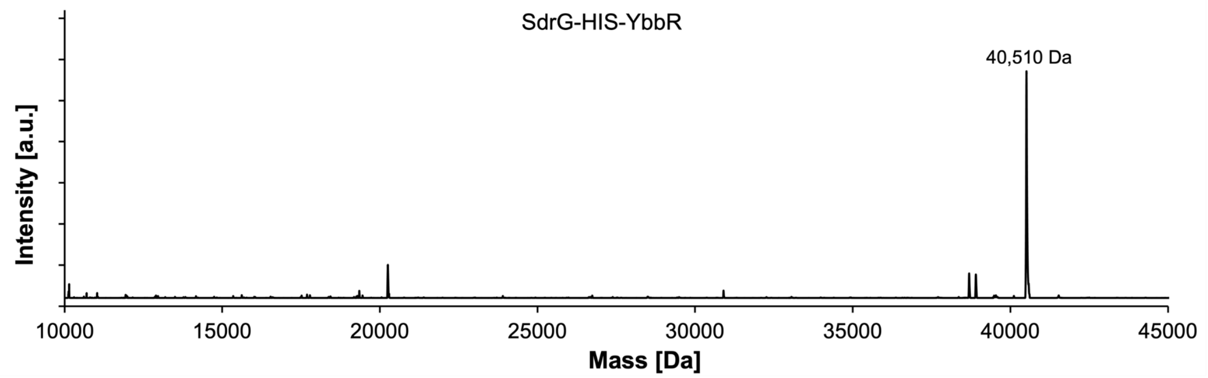

Figure S3. LC-MS spectra of undigested adhesins.

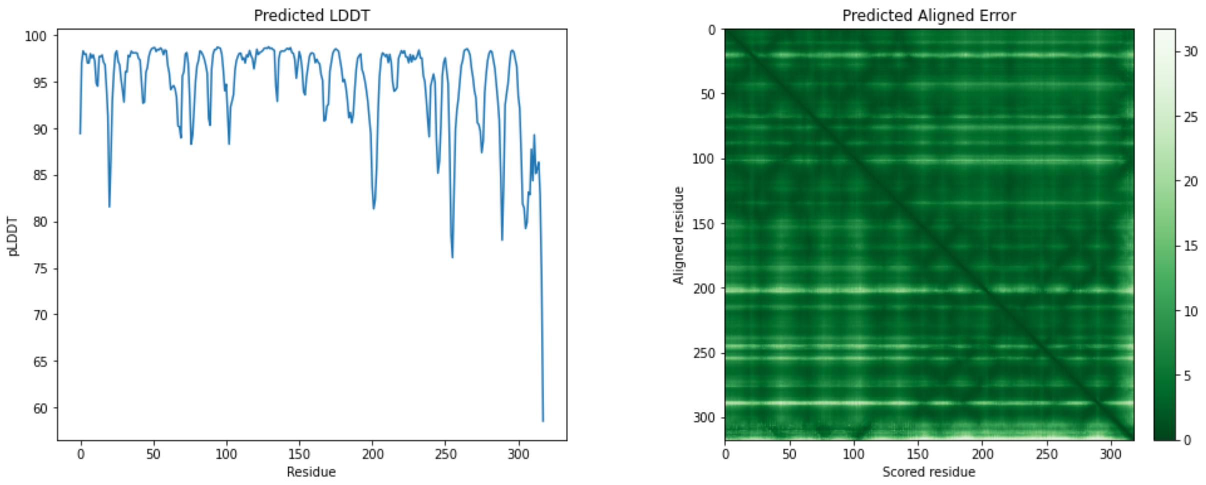

Figure S4. AlphaFold controls of FnBPB structure prediction. On the left side is a plot showing the predicted LDDT values obtained for each amino acid of the protein. Regions with pLDDT > 90 are expected to be modelled with high accuracy. Regions with pLDDT between 70 and 90 are expected to be modelled well (a generally good backbone prediction). Regions with pLDDT between 50 and 70 are low confidence and should be treated with caution. Here the residues with pLDDT values inferior to 90 are located in loops. On the right side is the predicted aligned error plot. It reports AlphaFold's expected position error at residue x, when the predicted and true structures are aligned on residue y. This is useful for assessing confidence in global features, especially domain packing. For residues x and y drawn from two different domains, a consistently low PAE at (x, y) suggests AlphaFold is confident about the relative domain positions.

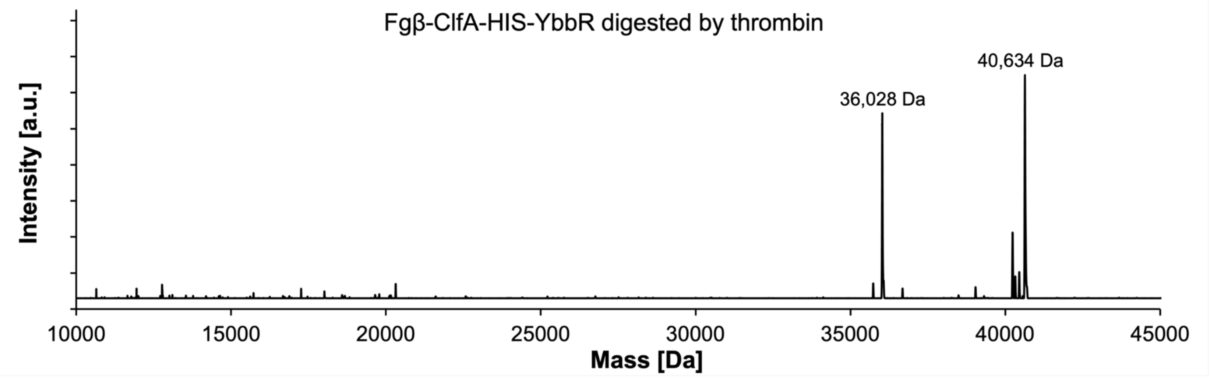
Fgβ-ClfA-HIS-YbbR digested by thrombin
40,634 Da
36,028 Da
Intensity [a.u.]
10000 15000 20000 25000 30000 35000 40000 45000
Mass [Da]

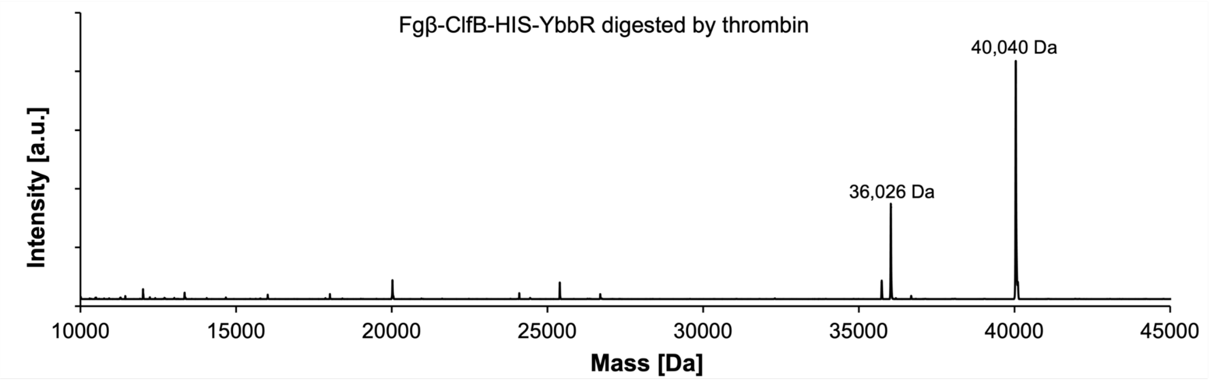
Fgβ-ClfB-HIS-YbbR digested by thrombin
40,040 Da
36,026 Da
Intensity [a.u.]
10000 15000 20000 25000 30000 35000 40000 45000
Mass [Da]

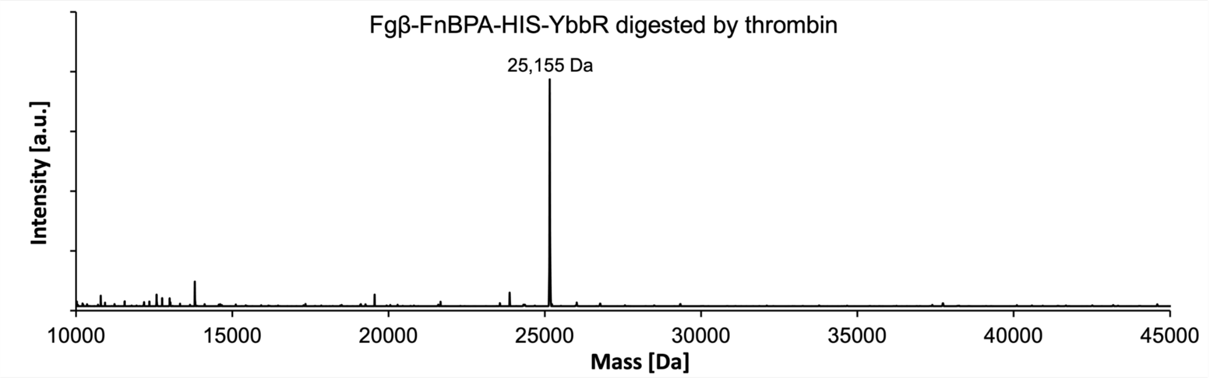
Fgβ-FnBPA-HIS-YbbR digested by thrombin
25,155 Da
Intensity [a.u.]
10000 15000 20000 25000 30000 35000 40000 45000
Mass [Da]

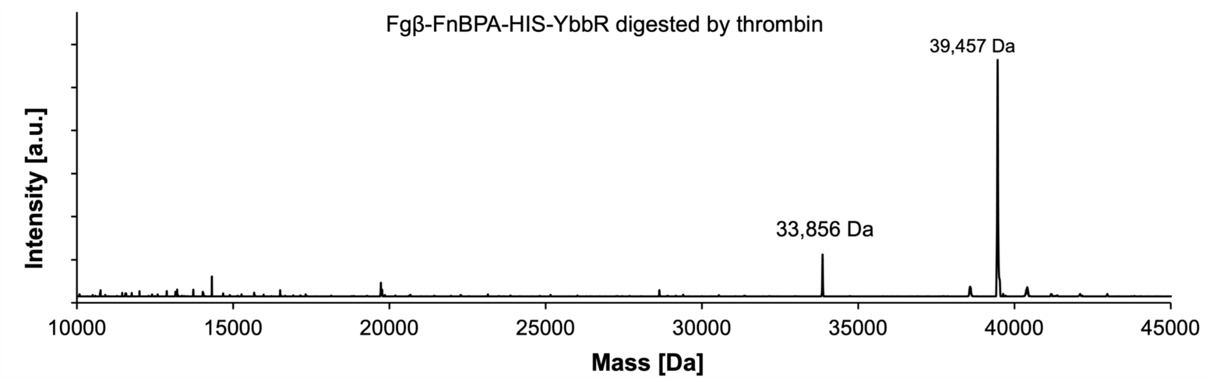
Fgβ-FnBPA-HIS-YbbR digested by thrombin
39,457 Da
33,856 Da
Intensity [a.u.]
10000 15000 20000 25000 30000 35000 40000 45000
Mass [Da]

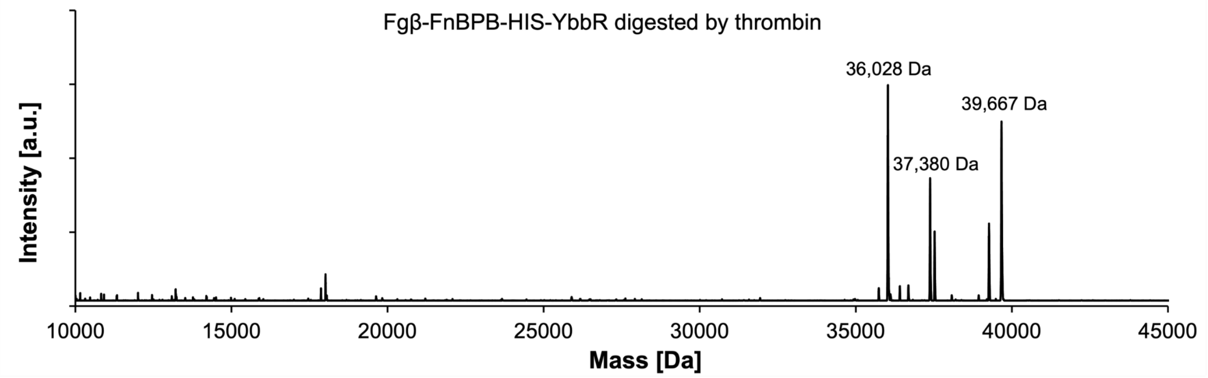
Fgβ-FnBPB-HIS-YbbR digested by thrombin
36,028 Da
39,667 Da
37,380 Da
Intensity [a.u.]
10000
15000
20000
25000
30000
35000
40000
45000
Mass [Da]

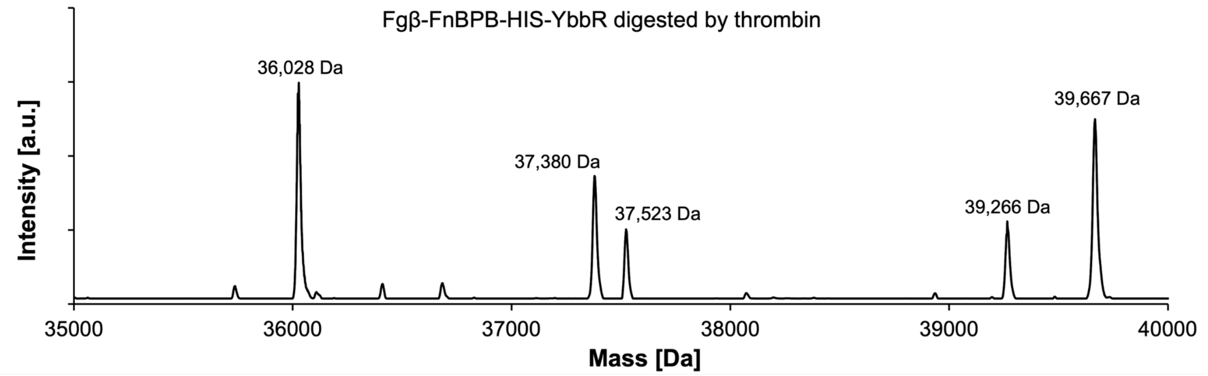
Fgβ-FnBPB-HIS-YbbR digested by thrombin
36,028 Da
39,667 Da
37,380 Da
37,523 Da
39,266 Da
Intensity [a.u.]
35000
36000
37000
38000
39000
40000
Mass [Da]

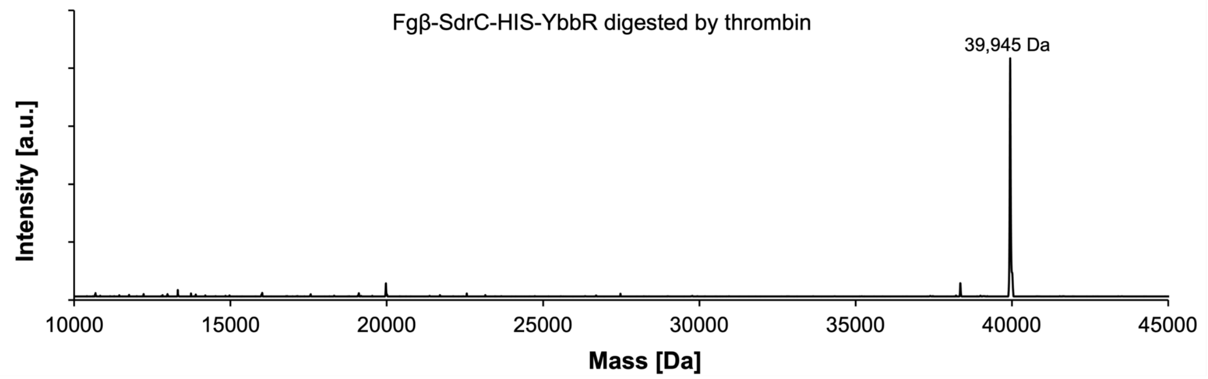
Fgβ-SdrC-HIS-YbbR digested by thrombin
39,945 Da
Intensity [a.u.]
10000
15000
20000
25000
30000
35000
40000
45000
Mass [Da]

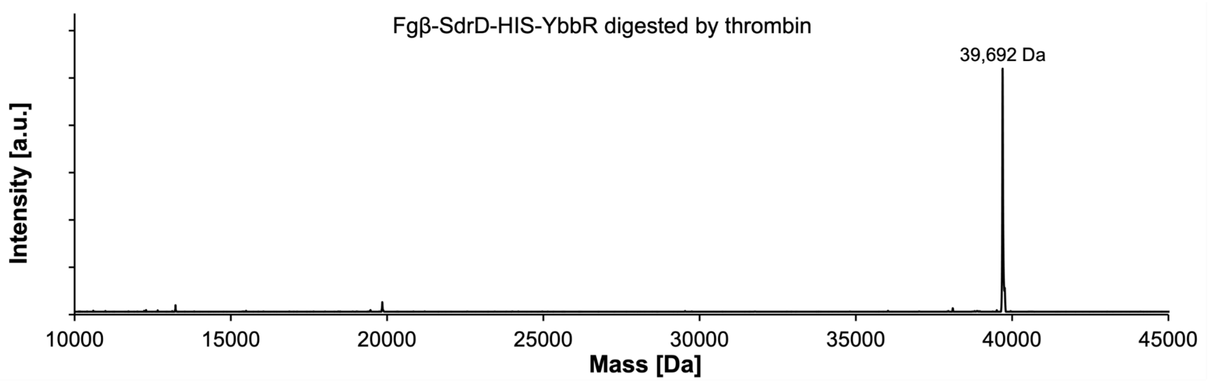
Fgβ-SdrD-HIS-YbbR digested by thrombin
39,692 Da
Intensity [a.u.]
10000
15000
20000
25000
30000
35000
40000
45000
Mass [Da]

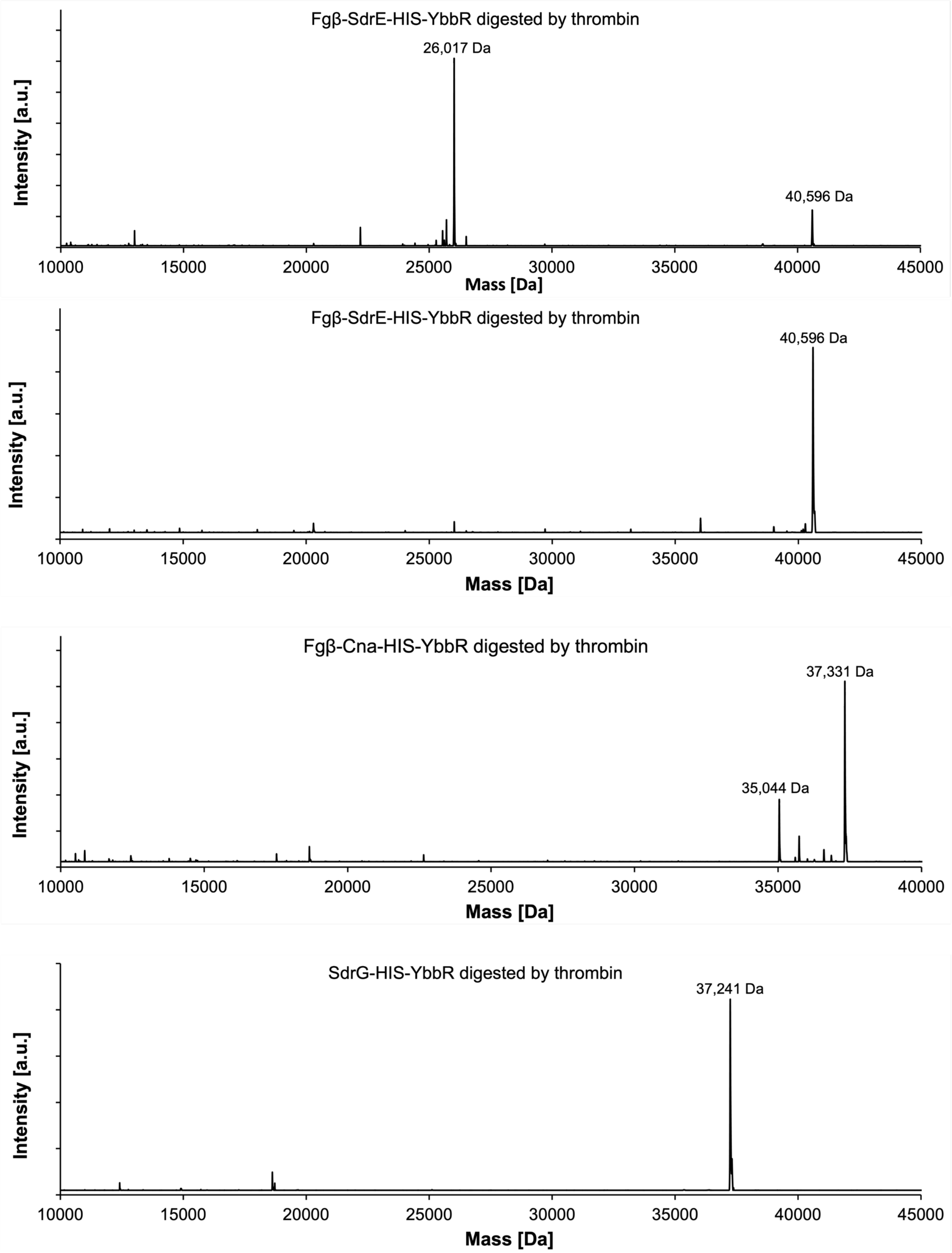

Figure S5. LC-MS spectra of thrombin digested adhesins. The 36,026 Da peak observed on some spectra corresponds to human thrombin.

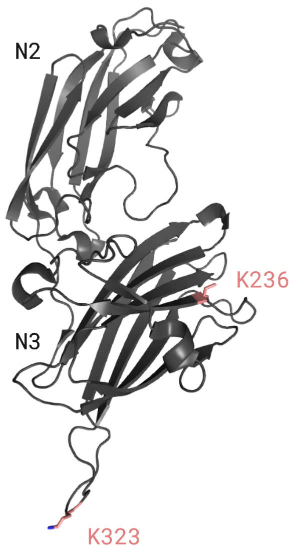

Figure S6. Localization of the thrombin cutting sites in FnBPA N2-N3 domains.

```
FnBPA_COL          GTDVTSKVTVEIGSIEGHNNTNKVEPHAGQRAVLKYKLKFENGLHQGDYFDFTLSNNVNT    60
FnBPA_NCTC8325     GTDVTSKVTVEIGSIEGHNNTNKVEPHAGQRAVLKYKLKFENGLHQGDYFDFTLSNNVNT    60
FnBPA_ISU926       GTDVTSKVTVTESSIEGHNNTNKVEPHAGQRAVLKYKLKFEDGLKKGDYFDFTLSNNVNT    60
FnBPA_Mu50         GTDVTSKVTVES-GSIEAPQGNKVEPHAGQRVVLKYKLKFADGLKRGDYFDFTLSNNVNT    59
FnBPA_N315         GTDVTSKVTVES-GSIEAPQGNKVEPHAGQRVVLKYKLKFADGLKRGDYFDFTLSNNVNT    59
FnBPA_MW2          GTDVTSKVTVES-GSIEAPQGNKVEPHAGQRVVLKYKLKFADGLKRGDYFDFTLSNNVNT    59
FnBPA_MSSA276      GTDVTSKVTVES-GSIEAPQGNKVEPHAGQRVVLKYKLKFADGLKRGDYFDFTLSNNVNT    59
FnBPA_ST228        GTDVTSKVTVES-GSIEAPQGNKVEPHAGQRVVLKYKLKFADGLKRGDYFDFTLSNNVNT    59
FnBPA_MRSA252      GTDVTSKVTVEDESKIEAPKGNNVQPHEGQRVVLKYKLKFQDGLKTGDYFDFTLSNNVNT    60
FnBPA_TCH60        GTDVTSKVTVEDESKIEAPKGNNVQPHEGQRVVLKYKLKFQDGLKTGDYFDFTLSNNVNT    60
FnBPA_ATCC25923    GTDVTSKVTVEDESEIEAPKGNNVQPHEGQRVVLKYKLKFQDGLKTGDYFDFTLSNNVNT    60
                   **********   .     : *:*:** ***.******** :**: **************

FnBPA_COL          HGVSTARKVPEIKNGSVVMATGEVLEGGKIRYTFTNDIEDKVDVTAELEINLFIDPKTVQ    120
FnBPA_NCTC8325     HGVSTARKVPEIKNGSVVMATGEVLEGGKIRYTFTNDIEDKVDVTAELEINLFIDPKTVQ    120
FnBPA_ISU926       YGVSTARKLPEIKNGSVVMATGQLLGDGKIRYTFTDYIDYKVNVIANLNLNLFIDPRIVK    120
FnBPA_Mu50         YGVSTARKVPEIKNGSVVMATGEILGNGNIRYTFTNEIEHKVEVTANLEINLFIDPKTVQ    119
FnBPA_N315         YGVSTARKVPEIKNGSVVMATGEILGNGNIRYTFTNEIEHKVEVTANLEINLFIDPKTVQ    119
FnBPA_MW2          YGVSTARKVPEIKNGSVVMATGEILGNGNIRYTFTNEIEHKVEVTANLEINLFIDPKTVQ    119
FnBPA_MSSA276      YGVSTARKVPEIKNGSVVMATGEILGNGNIRYTFTNEIEHKVEVTANLEINLFIDPKTVQ    119
FnBPA_ST228        YGVSTARKVPEIKNGSVVMATGEILGNGNIRYTFTNEIEHKVEVTANLEINLFIDPKTVQ    119
FnBPA_MRSA252      HGVATTRKVPDIKNGSLVMAKGQVLDNGRIRYTFTDYIKDKVNVTANLEINLFIDPKTVQ    120
FnBPA_TCH60        HGVATTRKVPDIKNGSLVMAKGQVLDNGRIRYTFTDYIKDKVNVTANLEINLFIDPKTVQ    120
FnBPA_ATCC25923    HGVATTRKVPDIKNGSLVMAKGQVLDNGRIRYTFTDYIKDKVNVTANLEINLFIDPKTVQ    120
                   :**:*:**:*:*****:***.*::* .*.******: *. **:* *:*::******: *:

                                              Plasmin
FnBPA_COL          TNGNQTITSTLNEEQTSKELDVKYKDGIGNYYANLNGSIETFNKANNRFSHVAFIKPNNG    180
FnBPA_NCTC8325     TNGNQTITSTLNEEQTSKELDVKYKDGIGNYYANLNGSIETFNKANNRFSHVAFIKPNNG    180
FnBPA_ISU926       NNGEETLTSKLNGKNTEKKIEVEYKDGVGKYYTNLNGSIETFNKADNKFTHVAYVKPING    180
FnBPA_Mu50         SNGEQKITSKLNGEETEKTIPVVYNPGVSNSYTNVNGSIETFNKESNKFTHIAYIKPMNG    179
FnBPA_N315         SNGEQKITSKLNGEETEKTIPVVYNPGVSNSYTNVNGSIETFNKESNKFTHIAYIKPMNG    179
FnBPA_MW2          SNGEQKITSKLNGEETEKTIPVVYNPGVSNSYTNVNGSIETFNKESNKFTHIAYIKPMNG    179
FnBPA_MSSA276      SNGEQKITSKLNGEETEKTIPVVYNPGVSNSYTNVNGSIETFNKESNKFTHIAYIKPMNG    179
FnBPA_ST228        SNGEQKITSKLNGEETEKTIPVVYNPGVSNSYTNVNGSIETFNKESNKFTHIAYIKPMNG    179
FnBPA_MRSA252      SNGQQTITSKLNGKETSGTMQITYKDGVKNQYTNVNGSIETFDKEKNKFTHVAYIKPING    180
FnBPA_TCH60        SNGQQTITSKLNGKETSGTMQITYKDGVKNQYTNVNGSIETFDKEKNKFTHVAYIKPING    180
FnBPA_ATCC25923    SNGQQTITSKLNGKETSGTMQITYKDGVKNQYTNVNGSIETFDKEKNKFTHVAYIKPING    180
                   .**::.:**.** ::*.  : : *: *: : *:*:*******:* .*:*:*:*::** **

                                             Thrombin
FnBPA_COL          K-TTSVTVTGTLMKGSNQNGNQPKVRIFEYLGNNEDIAKSVYANTTDTSKFKEVTSNMSG    239
FnBPA_NCTC8325     K-TTSVTVTGTLMKGSNQNGNQPKVRIFEYLGNNEDIAKSVYANTTDTSKFKEVTSNMSG    239
FnBPA_ISU926       NKSESVSITGSLTQGSNVSGDSPIVKVYEYQGKETDLPKSVSVNLTDNSKFKDVTSDMQN    240
FnBPA_Mu50         NQSNTVSVTGTLTEGSNLAGGQPTVKVYEYLGKKDELPQSVYANTSDTNKFKDVTKEMNG    239
FnBPA_N315         NQSNTVSVTGTLTEGSNLAGGQPTVKVYEYLGKKDELPQSVYANTSDTNKFKDVTKEMNG    239
FnBPA_MW2          NQSNTVSVTGTLTEGSNLAGGQPTVKVYEYLGKKDELPQSVYANTSDTNKFKDVTKEMNG    239
FnBPA_MSSA276      NQSNTVSVTGTLTEGSNLAGGQPTVKVYEYLGKKDELPQSVYANTSDTNKFKDVTKEMNG    239
FnBPA_ST228        NQSNTVSVTGTLTEGSNLAGGQPTVKVYEYLGKKDELPQSVYANTSDTNKFKDVTKEMNG    239
FnBPA_MRSA252      NNSDSVTVTGMLTQGSNENGTQPNVKIYEYVGVENGLPQSVYANTVDSTQLKDVTNQMGD    240
FnBPA_TCH60        NNSDSVTVTGMLTQGSNENGTQPNVKIYEYVGVENGLPQSVYANTVDSTQLKDVTNQMGD    240
FnBPA_ATCC25923    NNSDSVTVTGMLTQGSNENGTQPNVKIYEYVGVENGLPQSVYANTVDSTQLKDVTNQMGD    240
                   : : :*::** * :***  * .* *:::** * :  : :** .*  *..::*:**.:* .

                                                                     Plasmin/SAK
                                                                     Thrombin
FnBPA_COL          NLNLQNNGSYSLNIENLDKTYVVHYDGEYLNGTDEVDFRTQMVGHPEQLYKYY--YDRGY    297
FnBPA_NCTC8325     NLNLQNNGSYSLNIENLDKTYVVHYDGEYLNGTDEVDFRTQMVGHPEQLYKYY--YDRGY    297
FnBPA_ISU926       KLTVQENGNYQLNLEKLDKTYVIHYTGEYSKETDEVNFRTQVSAYPENSYRYYSYYNNHY    300
FnBPA_Mu50         KLSVQDNGSYSLNLDKLDKTYVIHYTGEYLQGSDQVNFRTELYGYPERAYKSYYVY-GGY    298
FnBPA_N315         KLSVQDNGSYSLNLDKLDKTYVIHYTGEYLQGSDQVNFRTELYGYPERAYKSYYVY-GGY    298
FnBPA_MW2          KLSVQDNGSYSLNLDKLDKTYVIHYTGEYLQGSDQVNFRTELYGYPERAYKSYYVY-GGY    298
FnBPA_MSSA276      KLSVQDNGSYSLNLDKLDKTYVIHYTGEYLQGSDQVNFRTELYGYPERAYKSYYVY-GGY    298
FnBPA_ST228        KLSVQDNGSYSLNLDKLDKTYVIHYTGEYLQGSDQVNFRTELYGYPERAYKSYYVY-GGY    298
FnBPA_MRSA252      KLKVQNNGSYSLNFDKLDKTYVIHYTGDYLNGTSEVNFRTQLTGYPENRYKTYYYYNNGY    300
FnBPA_TCH60        KLKVQNNGSYSLNFDKLDKTYVIHYTGDYLNGTSEVNFRTQLTGYPENRYKTYYYYNNGY    300
FnBPA_ATCC25923    KLKVQNNGSYSLNFDKLDKTYVIHYTGDYLNGTSEVNFRTQLTGYPENRYKTYYYYNNGY    300
                   :*.:*:**.*.**:::******:** *:* : :.:*:***:: .:**. *: *  *  *
```

```
FnBPA_COL           TLTWDNGLVLYSNKANGNGKN      318
FnBPA_NCTC8325      TLTWDNGLVLYSNKANGNEKN      318
FnBPA_ISU926        TLTWDNGLVLYSNKADGNGQN      321
FnBPA_Mu50          RLTWDNGLVLYSNKADGNGKN      319
FnBPA_N315          RLTWDNGLVLYSNKADGNGKN      319
FnBPA_MW2           RLTWDNGLVLYSNKADGNGKN      319
FnBPA_MSSA276       RLTWDNGLVLYSNKADGNGKN      319
FnBPA_ST228         RLTWDNGLVLYSNKADGNGKN      319
FnBPA_MRSA252       TLTWDNGLVLYSNKANGDGKY      321
FnBPA_TCH60         TLTWDNGLVLYSNKANGDGKY      321
FnBPA_ATCC25923     TLTWDNGLVLYSNKANGDGKY      321
                     **************:*: :
```

Figure S7. Sequence alignment of FnBPA N2-N3 domains of different *S*. *aureus* strains and localization of the cutting sites.

```
FnBPB_MW2           GTDVTSKVEVEEGSEIVGHNNKETNVVNPHNAERVTLKYKWKFEDGIKPGDYFDFTLSNN 60
FnBPB_MSSA276       GTDVTSKVEVEEGSEIVGHNNKETNVVNPHNAERVTLKYKWKFEDGIKPGDYFDFTLSNN 60
FnBPB_Mu50          GTDVTNKVEVTESS-LEG-HNKDSNIVNPHNAQRVTLKYKWKFGEGIKAGDYFDFTLSDN 58
FnBPB_N315          GTDVTNKVEVTESS-LEG-HNKDSNIVNPHNAQRVTLKYKWKFGEGIKAGDYFDFTLSDN 58
FnBPB_ATCC25923     GTDVTSKVKVTESS-LEG-HNKDSNIVNPHNAQRVTLKYKWKFGEGIKAGDYFDFTLSDN 58
FnBPB_TCH60         GTDVTSKVKVTESS-LEG-HNKDSNIVNPHNAQRVTLKYKWKFGEGIKAGDYFDFTLSDN 58
FnBPB_COL           GTDVTNKVEVEEGSEIVGHKQ-DTNVVNPHNAERVTLKYKWKFGEGIKAGDYFDFTLSDN 59
FnBPB_NCTC8325      GTDVTNKVEVEEGSEIVGHKQ-DTNVVNPHNAERVTLKYKWKFGEGIKAGDYFDFTLSDN 59
FnBPB_ISU926        GTDVTSKVKVEEGSEIVGHNNKETNVVNPHNAERVTLKYKWKFEDGIKPGDYFDFTLSNN 60
                    *****.**:* *.* : * :: ::*:******:********** :*** *********:*

FnBPB_MW2           VETHGISPLRKVPDIKSK-DDNILAVGKVMDERKIRYTFTDYINNKNNLMAELNLNLFID 119
FnBPB_MSSA276       VETHGISPLRKVPDIKSK-DDNILAVGKVMDERKIRYTFTDYINNKNNLMAELNLNLFID 119
FnBPB_Mu50          VETHGISTLRKVPEIKSSTEDKVMANGQVINERTIRYTFTDYINNKKDLTAELNLNLFID 118
FnBPB_N315          VETHGISTLRKVPEIKSSTEDKVMANGQVINERTIRYTFTDYINNKKDLTAELNLNLFID 118
FnBPB_ATCC25923     VETHGISTLRKVPEIKNSTEDKVMAIGEVIAERKIRYTFTDYIDNKKDLTAELSLNLFID 118
FnBPB_TCH60         VETHGISTLRKVPEIKNSTEDKVMAIGEVIAERKIRYTFTDYIDNKKDLTAELSLNLFID 118
FnBPB_COL           VETHGISTLRKVPEIKST-DGQVMATGEIIGERKVRYTFKEYVQEKKDLTAELSLNLFID 118
FnBPB_NCTC8325      VETHGISTLRKVPEIKST-DGQVMATGEIIGERKVRYTFKEYVQEKKDLTAELSLNLFID 118
FnBPB_ISU926        VETHGISPLRKVPDIKSK-DGQVMAVGEVNEERKIRYTFKEYVQGKKDLKAELDLNLFID 119
                    ******* *****:**.. :.:::* *::  **.:****.:*:: *::* ***.******

FnBPB_MW2           PTTVTKQGKQTVEVKLGENKISKEFDIKYLDGVKDNWGVTVNGRITLLDKENSKIHHLAY 179
FnBPB_MSSA276       PTTVTKQGKQTVEVKLGENKISKEFDIKYLDGVKDNWGVTVNGRITLLDKENSKIHHLAY 179
FnBPB_Mu50          PTTVTKQGSQKVEVTLGQNKVSKEFDIKYLDGVKDRMGVTVNGRIDTLNKEEGKFSHFAY 178
FnBPB_N315          PTTVTKQGSQKVEVTLGQNKVSKEFDIKYLDGVKDRMGVTVNGRIDTLNKEEGKFSHFAY 178
FnBPB_ATCC25923     PTTVTKQGKQKVEVTLGDKTIEKEFDIKYLDGVKDRMGVTVNGRIDTLNKADGKFSHFAY 178
FnBPB_TCH60         PTTVTKQGKQKVEVTLGDKTIEKEFDIKYLDGVKDRMGVTVNGRIDTLNKADGKFSHFAY 178
FnBPB_COL           PTTVTQKGNQNVEVKLGETTVSKIFNIQYLGGVRDNWGVTANGRIDTLNKVDGKFSHFAY 178
FnBPB_NCTC8325      PTTVTQKGNQNVEVKLGETTVSKIFNIQYLGGVRDNWGVTANGRIDTLNKVDGKFSHFAY 178
FnBPB_ISU926        PTTVTKRGNQNIEVTLGEKKISKQFDIKYLDGVKDNWGVTVNGRIHTLNKQEGRFSHFAY 179
                    *****::*.*.:**.**:..:.* *:*:**.**:*. ***.****  *:* :.:: *:**

FnBPB_MW2           INPKKSDMTSITINGFFAKGGLYTGNVPTVKVYEYLRSDELPESVYANTNDQEKFKDVTN 239
FnBPB_MSSA276       INPKKSDMTSITINGFFAKGGLYTGNVPTVKVYEYLRSDELPESVYANTNDQEKFKDVTN 239
FnBPB_Mu50          VKPNNQSLTSVTVTGQVTSGYKQSANNPTVKVYKHIGSDELAESVYAKLDDTSKFEDVTE 238
FnBPB_N315          VKPNNQSLTSVTVTGQVTSGYKQSANNPTVKVYKHIGSDELAESVYAKLDDTSKFEDVTE 238
FnBPB_ATCC25923     VKPNNQSLSSVTVTGQVTSGYKQNANKPTVKVYKHIGSDELAESVYAKLDDVNKFEDVTE 238
FnBPB_TCH60         VKPNNQSLSSVTVTGQVTSGYKQNANKPTVKVYKHIGSDELAESVYAKLDDVNKFEDVTE 238
FnBPB_COL           MKPNNQSLSSVTVTGQVTKGNKPGVNNPTVKVYKHIGSDDLAESVYAKLDDVSKFEDVTD 238
FnBPB_NCTC8325      MKPNNQSLSSVTVTGQVTKGNKPGVNNPTVKVYKHIGSDDLAESVYAKLDDVSKFEDVTD 238
FnBPB_ISU926        VKPNNQSLTSVTVTGQVTSGYKQNAKNPTVKVYKHIGSDELAESVYGDVLDETKFEDVTD 239
                    ::*::..::*:*:.* .:.*     : ******::: **:* ****..  *  **:***:

FnBPB_MW2           DMSDKLTLSENGSYKLTLDALN-KKSYVVSFEGKYNENDKELLFRTNLHGYHANYGY--- 295
FnBPB_MSSA276       DMSDKLTLSENGSYKLTLDALN-KKSYVVSFEGKYNENDKELLFRTNLHGYHANYGY--- 295
FnBPB_Mu50          KVN--LSYTSNGGYTLNLGDLDNSKDYVIKYEGEYDQNAKDLNFRTHLSGYHKYYPYYPY 296
FnBPB_N315          KVN—LSYTSNGGYTLNLGDLDNSKDYVIKYEGEYDQNAKDLNFRTHLSGYHKYYPYYPY  296
FnBPB_ATCC25923     KVN--LSYTSNGGYTLNLSDLDNSNDYVIKYEGEYDQNAKDLNFRTHLSGYHKNYPYYPY 296
FnBPB_TCH60         KVN—LSYTSNGGYTLNLSDLDNSNDYVIKYEGEYDQNAKDLNFRTHLSGYHKNYPYYPY  296
FnBPB_COL           NMS--LDFDTNGGYSLNFNNLDQSKNYVIKYEGYYDSNASNLEFQTHLFGYYNYY----- 291
FnBPB_NCTC8325      NMS--LDFDTNGGYSLNFNNLDQSKNYVIKYEGYYDSNASNLEFQTHLFGYYNYY----- 291
FnBPB_ISU926        KLS--LTLTGNGGYTLDFSELDKTKNYVIKYEGDYNSSASDLNFQTKLSGYHNYY----- 292
                    .:.  *    **.*.* :. *: .:.**:.:** *:.. .:* *:*:* **:   *

                                                    Thrombin & Plasmin/SAK
                                                Plasmin & Plasmin/SAK
                                                  Plasmin/SAK
FnBPB_MW2           YYYYPVSLTWDNGVAFYSNNAQGDGKDKPN 325
FnBPB_MSSA276       YYYYPVSLTWDNGVAFYSNNAQGDGKDKPN 325
FnBPB_Mu50          YPYYPVQLTWNNGVAFYSNNAKGDGKDKPN 326
FnBPB_N315          YPYYPVQLTWNNGVAFYSNNAKGDGKDKPN 326
FnBPB_ATCC25923     YSYYPVQLTWNNGVAFYSNNAQGDGKDKPN 326
FnBPB_TCH60         YSYYPVQLTWNNGVAFYSNNAQGDGKDKPN 326
FnBPB_COL           Y---TSNLTWKNGVAFYSNNAQGDGKDKLK 318
FnBPB_NCTC8325      Y---TSNLTWKNGVAFYSNNAQGDGKDKLK 318
FnBPB_ISU926        YSYYPVQLTWNNGVAFYSNNAQGDGKDKPK 322
                    *     .***.**********:****** :
```

Figure S8. Sequence alignment of FnBPB N2-N3 domains of different *S. aureus* strains

```
Cna_MW2          ARDISSTNVTDLTVSPTKIEDGGKTTVKMTFDDKSVKIQNGDTIKVAWPTSGTVKIEGYS      60
Cna_MSSA276      ARDISSTNVTDLTVSPTKIEDGGKTTVKMTFDDKSVKIQNGDTIKVAWPTSGTVKIEGYS      60
Cna_NCTC6131     ARDISSTNVTDLTVSPSKIEDGGKTTVKMTFDDKNGKIQNGDMIKVAWPTSGTVKIEGYS      60
Cna_MRSA252      ARDISSTNVTDLTVSPSKIEDGGKTTVKMTFDDKNGKIQNGDTIKVAWPTSGTVKIEGYS      60
Cna_ATCC25923    ARDISSTNVTDLTVSPSKIEDGGKTTVKMTFDDKNGKIQNGDTIKVAWPTSGTVKIEGYS      60
Cna_TCH60        ARDISSTNVTDLTVSPSKIEDGGKTTVKMTFDDKNGKIQNGDTIKVAWPTSGTVKIEGYS      60
Cna_ST228        ARDISSTNVTDLTVSPSKIEDGGKTTVKMTFDDKNGKIQNGDTIKVAWPTSGTVKIEGYS      60
Cna_ISU926       ARDISSTNVTDLTVSPSKIEDGGKTTVKMTFDDKNGKIQNGDTIKVAWPTSGTVKIEGYS      60
                 ****************:*****************. ****** *****************

Cna_MW2          KTVPLTVKGEQVGQAVITPDGATITFNDKVEKLSDVSGFAEFEVQGRNLTQTNTSDDKVA      120
Cna_MSSA276      KTVPLTVKGEQVGQAVITPDGATITFNDKVEKLSDVSGFAEFEVQGRNLTQTNTSDDKVA      120
Cna_NCTC6131     KTVPLTVKGEQVGQAVITPDGATITFNDKVEKLSDVSGFAEFEVQGRNLTQTNTSDDKVA      120
Cna_MRSA252      KTVSLTVKGEQVGQAVITPDGATITFNDKVEKLSDVSGFAEFEVQGRNLTQTNTSDDKVA      120
Cna_ATCC25923    KTVSLTVKGEQVGQAVITPDGATITFNDKVEKLSDVSGFAEFEVQGRNLTQTNTSDDKVA      120
Cna_TCH60        KTVSLTVKGEQVGQAVITPDGATITFNDKVEKLSDVSGFAEFEVQGRNLTQTNTSDDKVA      120
Cna_ST228        KTVSLTVKGEQVGQAVITPDGATITFNDKVEKLSDVSGFAEFEVQGRNLTQTNTSDDKVA      120
Cna_ISU926       KTVPLTVKGEQVGQAVITPDGATITFNDKVEKLSDVSGFAEFEVQGRNLTQTNTSDDKVA      120
                 *** ********************************************************

Cna_MW2          TITSGNKSTNVTVHKSEAGTSSVFYYKTGDMLPEDTTHVRWFLNINNEKRYVSKDITIKD      180
Cna_MSSA276      TITSGNKSTNVTVHKSEAGTSSVFYYKTGDMLPEDTTHVRWFLNINNEKRYVSKDITIKD      180
Cna_NCTC6131     TITSGNKSTNVTVHKSEAGTSSVFYYKTGDMLPEDTTHVRWFLNINNEKSYVSKDITIKD      180
Cna_MRSA252      TITSGNKSTNVTVHKSEAGTSSVFYYKTGDMLPEDTTHVRWFLNINNEKRYVSKDITIKD      180
Cna_ATCC25923    TITSGNKSTNVTVHKSEAGTSSVFYYKTGDMLPEDTTHVRWFLNINNEKRYVSKDITIKD      180
Cna_TCH60        TITSGNKSTNVTVHKSEAGTSSVFYYKTGDMLPEDTTHVRWFLNINNEKRYVSKDITIKD      180
Cna_ST228        TITSGNKSTNVTVHKSEAGTSSVFYYKTGDMLPEDTTHVRWFLNINNEKRYVSKDITIKD      180
Cna_ISU926       TITSGNKSTNVTVHKSEAGTSSVFYYKTGDMLPEDTTHVRWFLNINNEKSYVSKDITIKD      180
                 ************************************************* **********

Cna_MW2          QIQGGQQLDLSTLNINVTGTHSNYYSGSNAITDFEKAFPGSKITVDNTKNTIDVTIPQGY      240
Cna_MSSA276      QIQGGQQLDLSTLNINVTGTHSNYYSGSNAITDFEKAFPGSKITVDNTKNTIDVTIPQGY      240
Cna_NCTC6131     QIQGGQQLDLSTLNINVTGTHSNYYSGQSAITDFEKAFPGSKITVDNTKNTIDVTIPQGY      240
Cna_MRSA252      QIQGGQQLDLSTLNINVTGTHSNYYSGPNAITDFEKAFPGSKITVDNTKNTIDVTIPQGY      240
Cna_ATCC25923    QIQGGQQLDLSTLNINVTGTHSNYYSGPNAITDFEKAFPGSKITVDNTKNTIDVTIPQGY      240
Cna_TCH60        QIQGGQQLDLSTLNINVTGTHSNYYSGPNAITDFEKAFPGSKITVDNTKNTIDVTIPQGY      240
Cna_ST228        QIQGGQQLDLSTLNINVTGTHSNYYSGPNAITDFEKAFPGSKITVDNTKNTIDVTIPQGY      240
Cna_ISU926       QIQGGQQLDLSTLNINVTGTHSDYYSGPNAITDFEKAFPGSKITVDNTKNTIDVTIPQGY      240
                 **********************:**** .*******************************

Cna_MW2          GSYNSFSINYKTKITNEQQKEFVNNSQAWYQEHGKEEVNGKSFNHTVHNINANAGIEGTV      300
Cna_MSSA276      GSYNSFSINYKTKITNEQQKEFVNNSQAWYQEHGKEEVNGKSFNHTVHNINANAGIEGTV      300
Cna_NCTC6131     GSYNSFSINYKTKITNEQQKEFVNNSQAWYQEHGKEEVNGKSFNHTVHNINANAGIEGTV      300
Cna_MRSA252      GSLNSFSINYKTKITNEQQKEFVNNSQAWYQEHGKEEVNGKAFNHTVHNINANAGIEGTV      300
Cna_ATCC25923    GSLNSFSINYKTKITNEQQKEFVNNSQAWYQEHGKEEVNGKAFNHTVHNINANAGIEGTV      300
Cna_TCH60        GSLNSFSINYKTKITNEQQKEFVNNSQAWYQEHGKEEVNGKAFNHTVHNINANAGIEGTV      300
Cna_ST228        GSLNSFSINYKTKITNEQQKEFVNNSQAWYQEHGKEEVNGKAFNHTVHNINANAGIEGTV      300
Cna_ISU926       GSLNSFSINYKTKITNEQQKEFVNNSQAWYQEHGKEEVNGKSFNHTVHNINANAGIEGTV      300
                 ** **************************************:******************

                 Plasmin
                     Thrombin & Plasmin & Plasmin/SAK
Cna_MW2          KGELK     305
Cna_MSSA276      KGELK     305
Cna_NCTC6131     KGELK     305
Cna_MRSA252      KGELK     305
Cna_ATCC25923    KGELK     305
Cna_TCH60        KGELK     305
Cna_ST228        KGELK     305
Cna_ISU926       KGELK     305
                 *****
```

Figure S9. Sequence alignment of Cna N1-N2 domains of different *S. aureus* strains

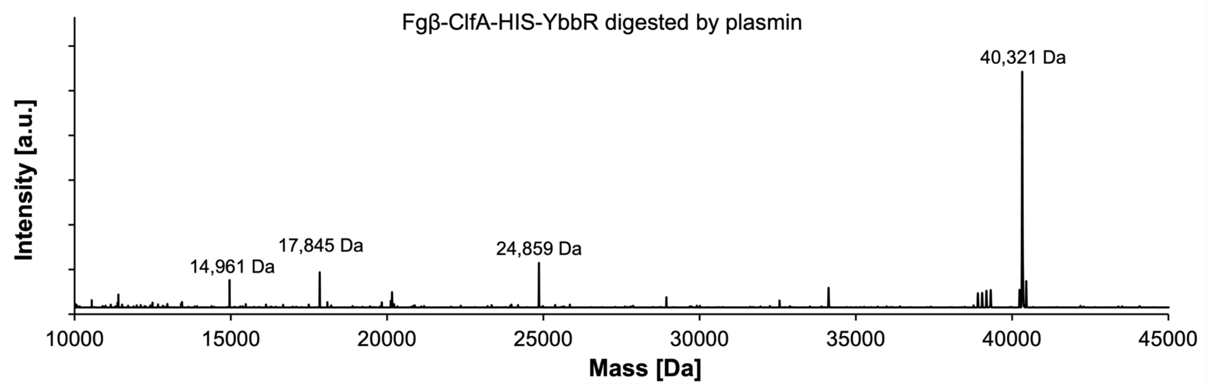
Fgβ-ClfA-HIS-YbbR digested by plasmin
40,321 Da
14,961 Da
17,845 Da
24,859 Da
Intensity [a.u.]
10000
15000
20000
25000
30000
35000
40000
45000
Mass [Da]

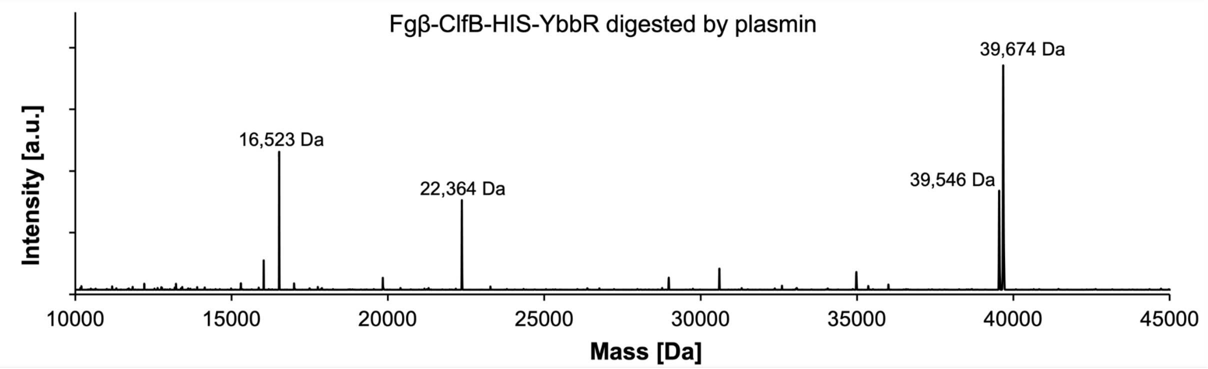
Fgβ-ClfB-HIS-YbbR digested by plasmin
39,674 Da
16,523 Da
22,364 Da
39,546 Da
Intensity [a.u.]
10000
15000
20000
25000
30000
35000
40000
45000
Mass [Da]

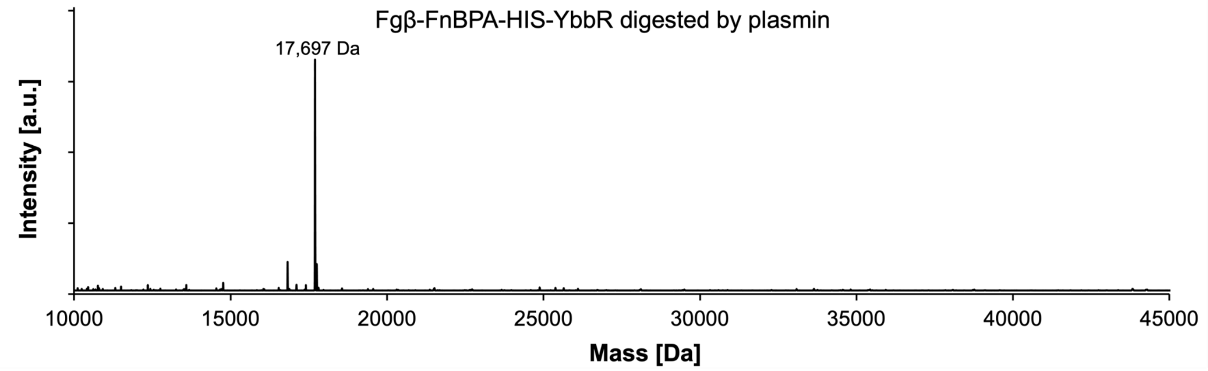
Fgβ-FnBPA-HIS-YbbR digested by plasmin
17,697 Da
Intensity [a.u.]
10000
15000
20000
25000
30000
35000
40000
45000
Mass [Da]

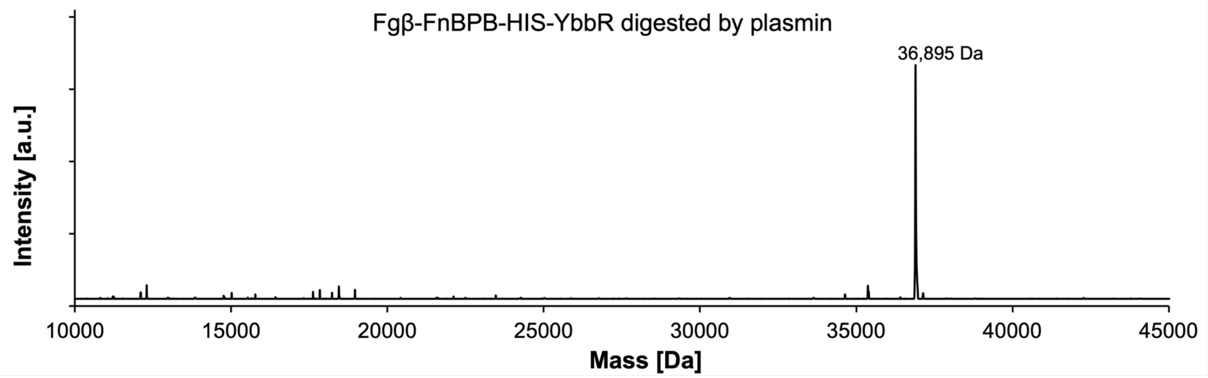
Fgβ-FnBPB-HIS-YbbR digested by plasmin
36,895 Da
Intensity [a.u.]
10000
15000
20000
25000
30000
35000
40000
45000
Mass [Da]

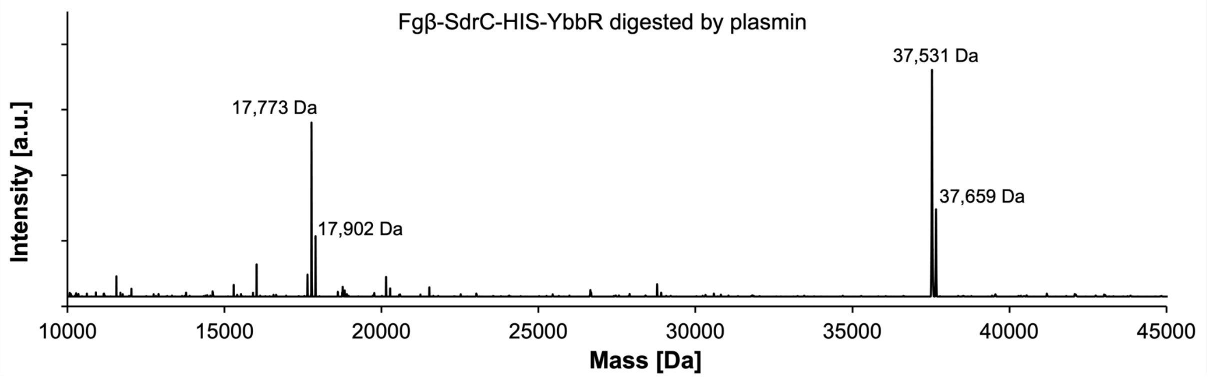
Fgβ-SdrC-HIS-YbbR digested by plasmin
37,531 Da
17,773 Da
37,659 Da
17,902 Da
Intensity [a.u.]
10000
15000
20000
25000
30000
35000
40000
45000
Mass [Da]

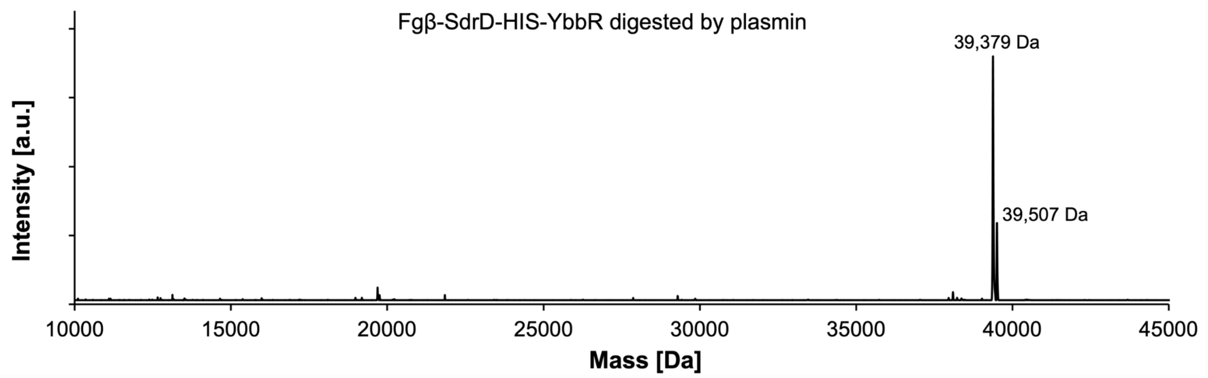
Fgβ-SdrD-HIS-YbbR digested by plasmin
39,379 Da
39,507 Da
Intensity [a.u.]
10000
15000
20000
25000
30000
35000
40000
45000
Mass [Da]

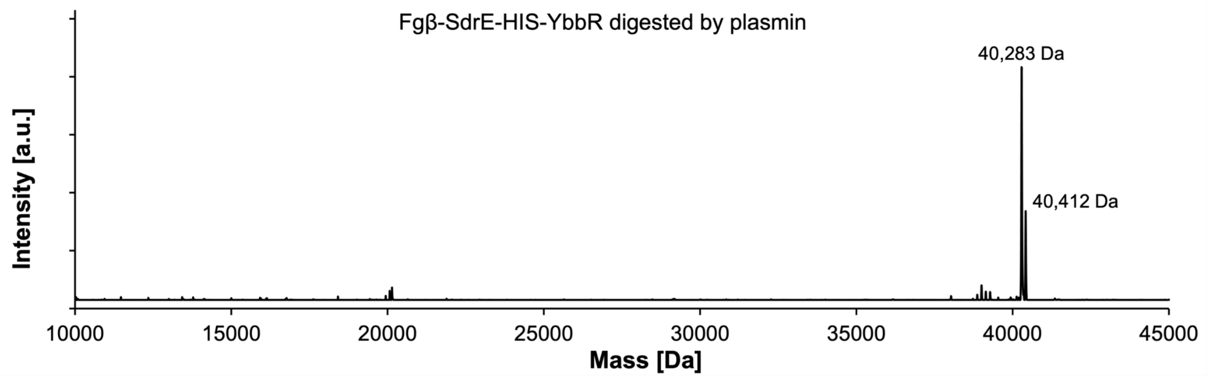
Fgβ-SdrE-HIS-YbbR digested by plasmin
40,283 Da
40,412 Da
Intensity [a.u.]
10000
15000
20000
25000
30000
35000
40000
45000
Mass [Da]

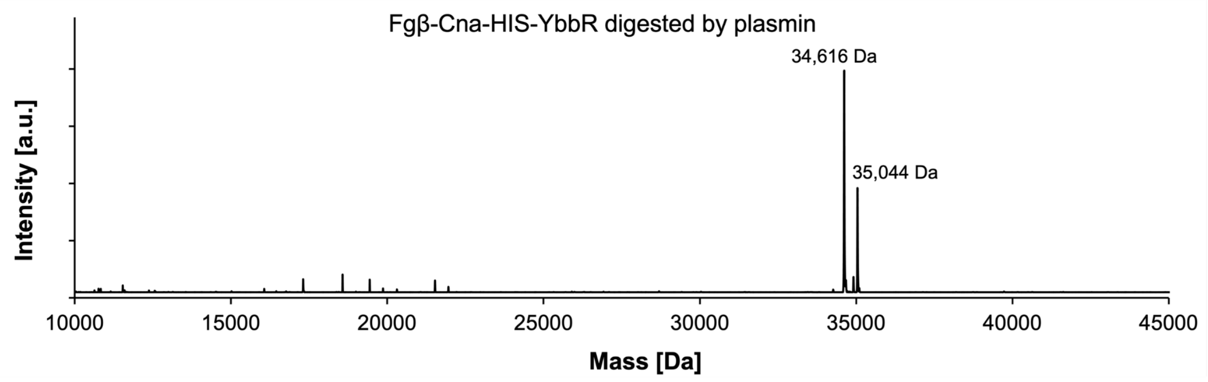
Fgβ-Cna-HIS-YbbR digested by plasmin
34,616 Da
35,044 Da
Intensity [a.u.]
10000
15000
20000
25000
30000
35000
40000
45000
Mass [Da]

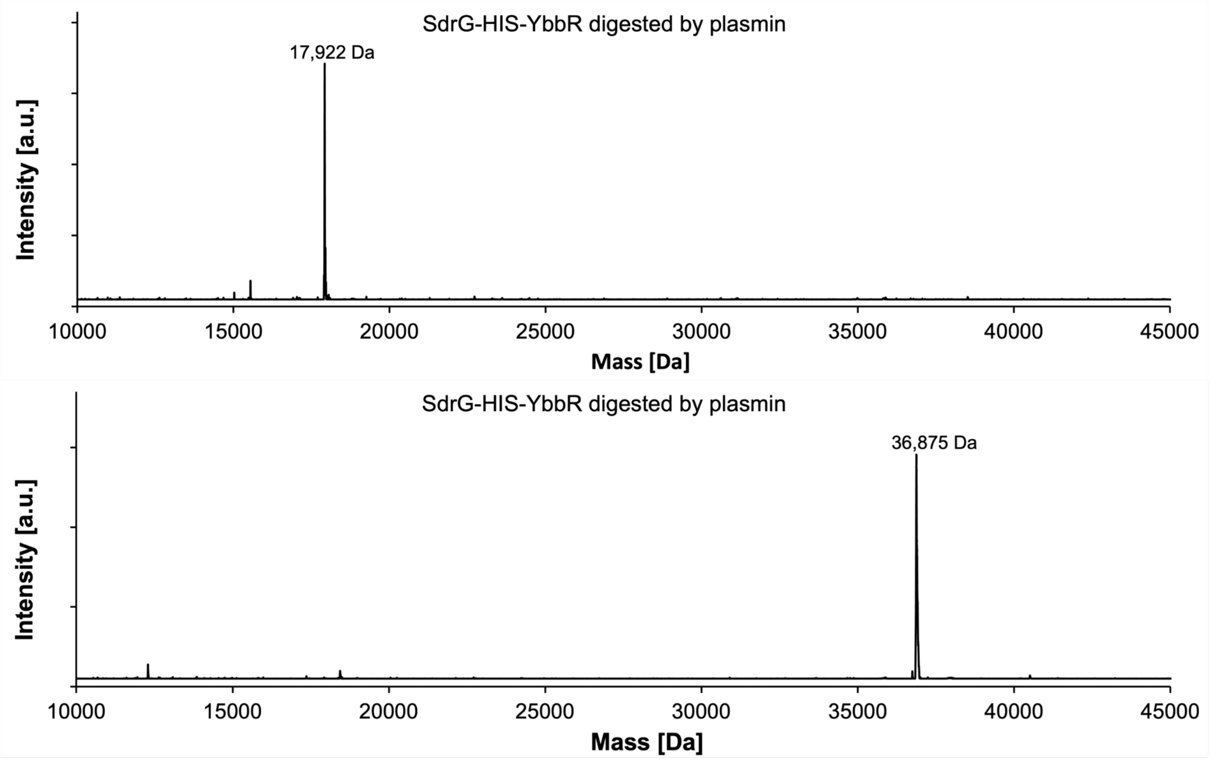

Figure S10. LC-MS spectra of plasmin digested adhesins.

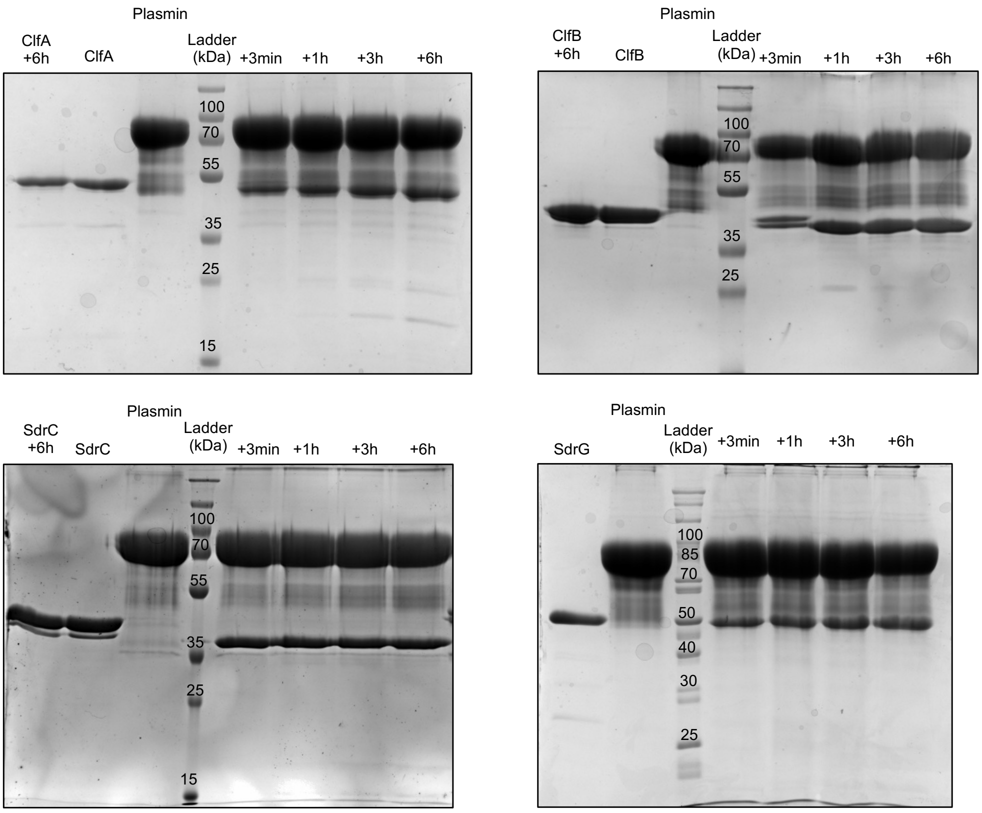

Figure S11. SDS-gel analysis of plasmin digestion kinetics.

```
ClfA_Mu50          VAADAPAAGTDITNQLTDVKVTIDSGTTVYPHQAGYVKLNYGFSVPNSAVKGDTFKITVP      60
ClfA_N315          VAADAPAAGTDITNQLTDVKVTIDSGTTVYPHQAGYVKLNYGFSVPNSAVKGDTFKITVP      60
ClfA_MW2           VAADAPAAGKDITNQLTNVTVGIDSGDTVYPHQAGYVKLNYGFSVPNSAVKGDTFKITVP      60
ClfA_MSSA276       VAADAPAAGKDITNQLTNVTVGIDSGDTVYPHQAGYVKLNYGFSVPNSAVKGDTFKITVP      60
ClfA_MRSA252       VAADAPAAGKDITNQLTNVTVGIDSGDTVYPHQAGYVKLNYGFSVPNEAVQGDTFKITVP      60
ClfA_ATCC25923     VAADAPAAGKDITNQLTNVTVGIDSGDTVYPHQAGYVKLNYGFSVPNEAVQGDTFKITVP      60
ClfA_TCH60         VAADAPAAGKDITNQLTNVTVGIDSGDTVYPHQAGYVKLNYGFSVPNEAVQGDTFKITVP      60
ClfA_ISU926        AAADAPAVGTDITNQLTNVTVGIDSGTTVYPHQAGYVKLNYGFSVPNSAVQGDTFKITVP      60
ClfA_COL           VAADAPAAGTDITNQLTNVTVGIDSGTTVYPHQAGYVKLNYGFSVPNSAVKGDTFKITVP      60
ClfA_NCTC8325      VAADAPVAGTDITNQLTNVTVGIDSGTTVYPHQAGYVKLNYGFSVPNSAVKGDTFKITVP      60
                   .*****..*.*******:*.* **** ********************.**:*********

ClfA_Mu50          KELNLNGVTSTAKVPPIMAGDQVLANGVIDSDGNVIYTFTDYVDNKENVTANITMPAYID      120
ClfA_N315          KELNLNGVTSTAKVPPIMAGDQVLANGVIDSDGNVIYTFTDYVDNKENVTANITMPAYID      120
ClfA_MW2           KELNLNGVTSTAKVPPIMAGDQVLANGVIDSDGNVIYTFTDYVDTKENVTANITMPAYID      120
ClfA_MSSA276       KELNLNGVTSTAKVPPIMAGDQVLANGVIDSDGNVIYTFTDYVDTKENVTANITMPAYID      120
ClfA_MRSA252       KELNLNGVTSTAKVPPIMAGDQVLANGVIDSDGNVIYTFTDYVNTKDDVKATLTMPAYID      120
ClfA_ATCC25923     KELNLNGVTSTAKVPPIMAGDQVLANGVIDSDGNVIYTFTDYVNTKDDVKATLTMPAYID      120
ClfA_TCH60         KELNLNGVTSTAKVPPIMAGDQVLANGVIDSDGNVIYTFTDYVNTKDDVKATLTMPAYID      120
ClfA_ISU926        KELNLNGVTSTAKVPPIMAGDQVLANGVIDSDGNVIYTFTDYVDTKNDVKATLTVPAYID      120
ClfA_COL           KELNLNGVTSTAKVPPIMAGDQVLANGVIDSDGNVIYTFTDYVNTKDDVKATLTMPAYID      120
ClfA_NCTC8325      KELNLNGVTSTAKVPPIMAGDQVLANGVIDSDGNVIYTFTDYVNTKDDVKATLTMPAYID      120
                   *******************************************:.*::*.*.:*:*****

                        Plasmin                     Plasmin
ClfA_Mu50          PENVTKTGNVTLTTGIGTNTASKTVLIDYEKYGQFHNLSIKGTIDQIDKTNNTYRQTIYV      180
ClfA_N315          PENVTKTGNVTLTTGIGTNTASKTVLIDYEKYGQFHNLSIKGTIDQIDKTNNTYRQTIYV      180
ClfA_MW2           PENVTKTGNVTLTTGIGSTTANKTVLVDYEKYGKFYNLSIKGTIDQIDKTNNTYRQTIYV      180
ClfA_MSSA276       PENVTKTGNVTLTTGIGSTTANKTVLVDYEKYGKFYNLSIKGTIDQIDKTNNTYRQTIYV      180
ClfA_MRSA252       PENVTKTGNVTLATGIGSTTANKTVLVDYEKYGKFYNLSIKGTIDQIDKTNNTYRQTIYV      180
ClfA_ATCC25923     PENVTKTGNVTLATGIGSTTANKTVLVDYEKYGKFYNLSIKGTIDQIDKTNNTYRQTIYV      180
ClfA_TCH60         PENVTKTGNVTLATGIGSTTANKTVLVDYEKYGKFYNLSIKGTIDQIDKTNNTYRQTIYV      180
ClfA_ISU926        PENVTKTGNVTLTTGIGNTTANKTVLVDYEKYGKFYNLSIKGTIDQIDKTNNTYRQTIYV      180
ClfA_COL           PENVKKTGNVTLATGIGSTTANKTVLVDYEKYGKFYNLSIKGTIDQIDKTNNTYRQTIYV      180
ClfA_NCTC8325      PENVKKTGNVTLATGIGSTTANKTVLVDYEKYGKFYNLSIKGTIDQIDKTNNTYRQTIYV      180
                   ****.*******:****..**.****:******:*:************************

                                                       Plasmin
ClfA_Mu50          NPSGDNVVLPALTGNLIPNTKSNALIDAKNTDIKVYRVDNANDLSESYYVNPSDFEDVTN      240
ClfA_N315          NPSGDNVVLPALTGNLIPNTKSNALIDAKNTDIKVYRVDNANDLSESYYVNPSDFEDVTN      240
ClfA_MW2           NPSGDNVIAPVLTGNLKPNTDSNALIDQQNTSIKVYKVDNAADLSESYFVNPENFEDVTN      240
ClfA_MSSA276       NPSGDNVIAPVLTGNLKPNTDSNALIDQQNTSIKVYKVDNAADLSESYFVNPENFEDVTN      240
ClfA_MRSA252       NPSGDNVIAPVLTGNLKPNTDSNALIDAQNTSIKVYKVDNASDLSESYYVNPDNFEDVTD      240
ClfA_ATCC25923     NPSGDNVIAPVLTGNLKPNTDSNALIDAQNTSIKVYKVDNASDLSESYYVNPDNFEDVTD      240
ClfA_TCH60         NPSGDNVIAPVLTGNLKPNTDSNALIDAQNTSIKVYKVDNASDLSESYYVNPDNFEDVTD      240
ClfA_ISU926        NPSGDNVIAPVLTGNLKPNTDSNALIDQQNTSIKVYKVDNAADLSESYFVNPENFEDVTN      240
ClfA_COL           NPSGDNVIAPVLTGNLKPNTDSNALIDQQNTSIKVYKVDNAADLSESYFVNPENFEDVTN      240
ClfA_NCTC8325      NPSGDNVIAPVLTGNLKPNTDSNALIDQQNTSIKVYKVDNAADLSESYFVNPENFEDVTN      240
                   *******: *.***** ***.****** :**.****:**** ******:***.:*****:

ClfA_Mu50          QVRISFPNANQYKVEFPTDDDQITTPYIVVVNGHIDPASTGDLALRSTFYGYDSNFIWRS      300
ClfA_N315          QVRISFPNANQYKVEFPTDDDQITTPYIVVVNGHIDPASTGDLALRSTFYGYDSNFIWRS      300
ClfA_MW2           SVNITFPNPNQYKVEFNTPDDQITTPYIVVVNGHIDPNSKGDLALRSTLYGYDSRFVWRS      300
ClfA_MSSA276       SVNITFPNPNQYKVEFNTPDDQITTPYIVVVNGHIDPNSKGDLALRSTLYGYDSRFVWRS      300
ClfA_MRSA252       SVNITFPNPNQYKVEFNTPDDQITTPYIVVVNGHIDPNSKGDLALRSTLYGYNSNIIWRS      300
ClfA_ATCC25923     SVNITFPNPNQYKVEFNTPDDQITTPYIVVVNGHIDPNSKGDLALRSTLYGYNSNIIWRS      300
ClfA_TCH60         SVNITFPNPNQYKVEFNTPDDQITTPYIVVVNGHIDPNSKGDLALRSTLYGYNSNIIWRS      300
ClfA_ISU926        SVNITFPNPNQYKVEFNTPDDQITTPYIVVVNGHIDPNSKGDLALRSTLYGYNSNIIWRS      300
ClfA_COL           SVNITFPNPNQYKVEFNTPDDQITTPYIVVVNGHIDPNSKGDLALRSTLYGYNSNIIWRS      300
ClfA_NCTC8325      SVNITFPNPNQYKVEFNTPDDQITTPYIVVVNGHIDPNSKGDLALRSTLYGYNSNIIWRS      300
                   .*.*:*** ******* * ****************** *.********:***:*.::***

ClfA_Mu50          MSWDNEVAFNNGSGSGDGIDKPVVPEQPDEPGEIEPIPE       339
ClfA_N315          MSWDNEVAFNNGSGSGDGIDKPVVPEQPDEPGEIEPIPE       339
ClfA_MW2           MSWDNEVAFNNGSGSGDGIDKPVVPEQPDEPGEIEPIPE       339
ClfA_MSSA276       MSWDNEVAFNNGSGSGDGIDKPVVPEQPDEPGEIEPIPE       339
ClfA_MRSA252       MSWDNEVAFNNGSGSGDGIDKPVVPEQPDEPGEIEPIPE       339
ClfA_ATCC25923     MSWDNEVAFNNGSGSGDGIDKPVVPEQPDEPGEIEPIPE       339
ClfA_TCH60         MSWDNEVAFNNGSGSGDGIDKPVVPEQPDEPGEIEPIPE       339
ClfA_ISU926        MSWDNEVAFNNGSGSGDGIDKPVVPEQPDEPGEIEPIPE       339
ClfA_COL           MSWDNEVAFNNGSGSGDGIDKPVVPEQPDEPGEIEPIPE       339
ClfA_NCTC8325      MSWDNEVAFNNGSGSGDGIDKPVVPEQPDEPGEIEPIPE       339
                   ***************************************
```

Figure S12. Sequence alignment of ClfA N2-N3 domains of different *S. aureus* strains.

```
ClfB_MW2            PVVNAADAKGTNVNDKVTAKDFQLEKTTFDPNQSGNTFMAANFTVTGQVKSGDYFTAKLP     60
ClfB_MSSA276        PVVNAADAKGTNVNDKVTAKDFQLEKTTFDPNQSGNTFMAANFTVTGQVKSGDYFTAKLP     60
ClfB_MRSA252        PVVNAADAKGTNVNDKVTASDFKLEKTAFDPNQSGNTFMAANFKVTGQVKSGDYFTAKLP     60
ClfB_ATCC25923      PVVNAADAKGTNVNDKVTASDFKLEKTAFDPNQSGNTFMAANFKVTGQVKSGDYFTAKLP     60
ClfB_TCH60          PVVNAADAKGTNVNDKVTASDFKLEKTAFDPNQSGNTFMAANFKVTGQVKSGDYFTAKLP     60
ClfB_COL            PVVNAADAKGTNVNDKVTASNFKLEKTTFDPNQSGNTFMAANFTVTDKVKSGDYFTAKLP     60
ClfB_Mu50           PVVNAADAKGTNVNDKVTASNFKLEKTTFDPNQSGNTFMAANFTVTDKVKSGDYFTAKLP     60
ClfB_N315           PVVNAADAKGTNVNDKVTASNFKLEKTTFDPNQSGNTFMAANFTVTDKVKSGDYFTAKLP     60
ClfB_NCTC8325       PVVNAADAKGTNVNDKVTASNFKLEKTTFDPNQSGNTFMAANFTVTDKVKSGDYFTAKLP     60
ClfB_ST228          PVVNAADAKGTNVNDKVTASNFKLEKTTFDPNQSGNTFMAANFTVTDKVKSGDYFTAKLP     60
ClfB_ISU926         PVVNAADAKGTNVNDKVTASNLQLQKTTFDPNQSGNTFMAANFTVTDKVKSGDYFTAKLP     60
                    *******************.:::*:**:***************.**.:************

ClfB_MW2            DSVTGNGDVDYSNSNNTMPIADIVNDKNEVVAKATYDILTKTYTFVFTDYVNDKQNINGK     120
ClfB_MSSA276        DSVTGNGDVDYSNSNNTMPIADIVNDKNEVVAKATYDILTKTYTFVFTDYVNDKQNINGK     120
ClfB_MRSA252        DSVTGNGDVDYSNSNNTMPIADIKSTNGDVVAKATYDILTKTYTFVFTDYVNDKENINGQ     120
ClfB_ATCC25923      DSVTGNGDVDYSNSNNTMPIADIKSTNGDVVAKATYDILTKTYTFVFTDYVNDKENINGQ     120
ClfB_TCH60          DSVTGNGDVDYSNSNNTMPIADIKSTNGDVVAKATYDILTKTYTFVFTDYVNDKENINGQ     120
ClfB_COL            DSLTGNGDVDYSNSNNTMPIADIKSTNGDVVAKATYDILTKTYTFVFTDYVNNKENINGQ     120
ClfB_Mu50           DSLTGNGDVDYSNSNNTMPIADIKSTNGDVVAKATYDILTKTYTFVFTDYVNNKENINGQ     120
ClfB_N315           DSLTGNGDVDYSNSNNTMPIADIKSTNGDVVAKATYDILTKTYTFVFTDYVNNKENINGQ     120
ClfB_NCTC8325       DSLTGNGDVDYSNSNNTMPIADIKSTNGDVVAKATYDILTKTYTFVFTDYVNNKENINGQ     120
ClfB_ST228          DSLTGNGDVDYSNSNNTMPIADIKSTNGDVVAKATYDILTKTYTFVFTDYVNNKENINGQ     120
ClfB_ISU926         DSLTGNGDVDYSNSNNTMPIADIKSTNGDVVAKATYDILTKTYTFVFTDYVNDKENINGQ     120
                    **:******************** . :.:***********************:*:****:

                                Plasmin
ClfB_MW2            FSLPLFTDRAKAPKSGTYDANINIADEMFNNKITYNYSSPIAGIDKPNGANISSQIIGVD     180
ClfB_MSSA276        FSLPLFTDRAKAPKSGTYDANINIADEMFNNKITYNYSSPIAGIDKPNGANISSQIIGVD     180
ClfB_MRSA252        FSLPLFTDRAKAPKSGTYDANINIADEMFDNKITYNYSSPIAGIDKPNGANISSQIIGVD     180
ClfB_ATCC25923      FSLPLFTDRAKAPKSGTYDANINIADEMFDNKITYNYSSPIAGIDKPNGANISSQIIGVD     180
ClfB_TCH60          FSLPLFTDRAKAPKSGTYDANINIADEMFDNKITYNYSSPIAGIDKPNGANISSQIIGVD     180
ClfB_COL            FSLPLFTDRAKAPKSGTYDANINIADEMFNNKITYNYSSPIAGIDKPNGANISSQIIGVD     180
ClfB_Mu50           FSLPLFTDRAKAPKSGTYDANINIADEMFNNKITYNYSSPIAGIDKPNGANISSQIIGVD     180
ClfB_N315           FSLPLFTDRAKAPKSGTYDANINIADEMFNNKITYNYSSPIAGIDKPNGANISSQIIGVD     180
ClfB_NCTC8325       FSLPLFTDRAKAPKSGTYDANINIADEMFNNKITYNYSSPIAGIDKPNGANISSQIIGVD     180
ClfB_ST228          FSLPLFTDRAKAPKSGTYDANINIADEMFNNKITYNYSSPIAGIDKPNGANISSQIIGVD     180
ClfB_ISU926         FSLPLFTDRAKAPKSGTYDANINIADEMFDNKITYNYSSPIAGIDKPNGANISSQIIGVD     180
                    *****************************:******************************

                            Plasmin
ClfB_MW2            TASGQNTYKQTVFVNPKQRVLGNTWVYIKGYQDKIEESSGKVSATDTKLRIFEVNDTSKL     240
ClfB_MSSA276        TASGQNTYKQTVFVNPKQRVLGNTWVYIKGYQDKIEESSGKVSATDTKLRIFEVNDTSKL     240
ClfB_MRSA252        TASGQNTYKQTVFVNPKQRVLGNTWVYIKGYQDKIEESSGKVSATDTKLRIFEVNDTSKL     240
ClfB_ATCC25923      TASGQNTYKQTVFVNPKQRVLGNTWVYIKGYQDKIEESSGKVSATDTKLRIFEVNDTSKL     240
ClfB_TCH60          TASGQNTYKQTVFVNPKQRVLGNTWVYIKGYQDKIEESSGKVSATDTKLRIFEVNDTSKL     240
ClfB_COL            TASGQNTYKQTVFVNPKQRVLGNTWVYIKGYQDKIEESSGKVSATDTKLRIFEVNDTSKL     240
ClfB_Mu50           TASGQNTYKQTVFVNPKQRVLGNTWVYIKGYQDKIEESSGKVSATDTKLRIFEVNDTSKL     240
ClfB_N315           TASGQNTYKQTVFVNPKQRVLGNTWVYIKGYQDKIEESSGKVSATDTKLRIFEVNDTSKL     240
ClfB_NCTC8325       TASGQNTYKQTVFVNPKQRVLGNTWVYIKGYQDKIEESSGKVSATDTKLRIFEVNDTSKL     240
ClfB_ST228          TASGQNTYKQTVFVNPKQRVLGNTWVYIKGYQDKIEESSGKVSATDTKLRIFEVNDTSKL     240
ClfB_ISU926         TASGQNTYKQTVFVNPKQRVLGNTWVYIKGYQDKIEESSGKVSATDTKLRIFEVNDTSKL     240
                    ************************************************************

ClfB_MW2            SDSYYADPNDSNLKEVTDQFKDKITYKYQNVASINFGDINKTYVVLVEGHYDKTGKNLKT     300
ClfB_MSSA276        SDSYYADPNDSNLKEVTDQFKDKITYKYQNVASINFGDINKTYVVLVEGHYDKTGKNLKT     300
ClfB_MRSA252        SDSYYADPNDSNLKEVTGEFKDKISYKYDNVASINFGDINKTYVVLVEGHYDNTGKNLKT     300
ClfB_ATCC25923      SDSYYADPNDSNLKEVTGEFKDKISYKYDNVASINFGDINKTYVVLVEGHYDNTGKNLKT     300
ClfB_TCH60          SDSYYADPNDSNLKEVTGEFKDKISYKYDNVASINFGDINKTYVVLVEGHYDNTGKNLKT     300
ClfB_COL            SDSYYADPNDSNLKEVTDQFKNRIYYEHPNVASIKFGDITKTYVVLVEGHYDNTGKNLKT     300
ClfB_Mu50           SDSYYADPNDSNLKEVTDQFKNRIYYEHPNVASIKFGDITKTYVVLVEGHYDNTGKNLKT     300
ClfB_N315           SDSYYADPNDSNLKEVTDQFKNRIYYEHPNVASIKFGDITKTYVVLVEGHYDNTGKNLKT     300
ClfB_NCTC8325       SDSYYADPNDSNLKEVTDQFKNRIYYEHPNVASIKFGDITKTYVVLVEGHYDNTGKNLKT     300
ClfB_ST228          SDSYYADPNDSNLKEVTDQFKNRIYYEHPNVASIKFGDITKTYVVLVEGHYDNTGKNLKT     300
ClfB_ISU926         SDSYYADPNDSNLKEVTGEFNNRIFYEHPNVASINFGDINKTYVVLVEGHYDNTGKNLKT     300
                    *****************.:*:::* *:: *****:****.************:*******
```

```
ClfB_MW2            QVIQENVDPATGKDYSIFGWNNENVVRYGGGSADGDSAV        339
ClfB_MSSA276        QVIQENVDPATGKDYSIFGWNNENVVRYGGGSADGDSAV        339
ClfB_MRSA252        QVIQENIDPATGKDYSIFGWNNENVVRYGGGSADGDSAV        339
ClfB_ATCC25923      QVIQENIDPATGKDYSIFGWNNENVVRYGGGSADGDSAV        339
ClfB_TCH60          QVIQENIDPATGKDYSIFGWNNENVVRYGGGSADGDSAV        339
ClfB_COL            QVIQENVDPVTNRDYSIFGWNNENVVRYGGGSADGDSAV        339
ClfB_Mu50           QVIQENVDPVTNRDYSIFGWNNENVVRYGGGSADGDSAV        339
ClfB_N315           QVIQENVDPVTNRDYSIFGWNNENVVRYGGGSADGDSAV        339
ClfB_NCTC8325       QVIQENVDPVTNRDYSIFGWNNENVVRYGGGSADGDSAV        339
ClfB_ST228          QVIQENVDPVTNRDYSIFGWNNENVVRYGGGSADGDSAV        339
ClfB_ISU926         QVIQENIDPATGKDYSIFGWNNENVVRYGGGSADGDSAV        339
                    ******:**.*.:**************************
```

Figure S13. Sequence alignment of ClfB N2-N3 domains of different *S. aureus* strains.

```
SdrC_Mu50          APQQGTNVNDKVHFTNIDIAIDKGHVNKTTGNTEFWATSSDVLKLKANYTIDDSVKEGDT      60
SdrC_N315          APQQGTNVNDKVHFTNIDIAIDKGHVNKTTGNTEFWATSSDVLKLKANYTIDDSVKEGDT      60
SdrC_ST228         APQQGTNVNDKVHFTNIDIAIDKGHVNKTTGNTEFWATSSDVLKLKANYTIDDSVKEGDT      60
SdrC_COL           APQQGTNVNDKVHFSNIDIAIDKGHVNQTTGKTEFWATSSDVLKLKANYTIDDSVKEGDT      60
SdrC_NCTC8325      APQQGTNVNDKVHFSNIDIAIDKGHVNQTTGKTEFWATSSDVLKLKANYTIDDSVKEGDT      60
SdrC_MW2           APQQGTNVNDKVHFSNIDIAIDKGHLNKDTGKTEFWATSSDVLKLKANYTIDDSVKEGDT      60
SdrC_MSSA276       APQQGTNVNDKVHFSNIDIAIDKGHLNKDTGKTEFWATSSDVLKLKANYTIDDSVKEGDT      60
SdrC_ISU926        APQQGTNVNDKVHFSNIDIAIDKGHVNSTTGKTEFWATSSDVLKLKANYTIDDSVKEGDT      60
SdrC_MRSA252       APQQGTNVNDKVHFSNIDIAIDKGHVNSTTGKTEFWATSSDVLKLKANYTIDDSVKEGDT      60
SdrC_ATCC25923     APQQGTNVNDKVHFSNIDIAIDKGHVNSTTGKTEFWATSSDVLKLKANYTIDDSVKEGDT      60
SdrC_TCH60         APQQGTNVNDKVHFSNIDIAIDKGHVNSTTGKTEFWATSSDVLKLKANYTIDDSVKEGDT      60
                   **************:**********:*. **:****************************

SdrC_Mu50          FTFKYGQYFRPGSVRLPSQTQNLYNAQGNIIAKGIYDSKTNTTTYTFTNYVDQYTNVSGS      120
SdrC_N315          FTFKYGQYFRPGSVRLPSQTQNLYNAQGNIIAKGIYDSKTNTTTYTFTNYVDQYTNVSGS      120
SdrC_ST228         FTFKYGQYFRPGSVRLPSQTQNLYNAQGNIIAKGIYDSKTNTTTYTFTNYVDQYTNVSGS      120
SdrC_COL           FTFKYGQYFRPGSVRLPSQTQNLYNAQGNIIAKGIYDSTTNTTTYTFTNYVDQYTNVRGS      120
SdrC_NCTC8325      FTFKYGQYFRPGSVRLPSQTQNLYNAQGNIIAKGIYDSTTNTTTYTFTNYVDQYTNVRGS      120
SdrC_MW2           FTFKYGQYFRPGSVRLPSQTQNLYNAQGNIIAKGIYDSTTNTTTYTFTNYVDQYTNVSGS      120
SdrC_MSSA276       FTFKYGQYFRPGSVRLPSQTQNLYNAQGNIIAKGIYDSTTNTTTYTFTNYVDQYTNVSGS      120
SdrC_ISU926        FTFKYGQYFRPGSVRLPSQTQNLYNAQGNIIAKGIYDSTTNTTTYTFTNYVDQYTNVSGS      120
SdrC_MRSA252       FTFKYGQYFRPGSVRLPSQTQNLYNAQGNIIAKGIYDSTTNTTTYTFTNYVDQYTNVSGS      120
SdrC_ATCC25923     FTFKYGQYFRPGSVRLPSQTQNLYNAQGNIIAKGIYDSTTNTTTYTFTNYVDQYTNVSGS      120
SdrC_TCH60         FTFKYGQYFRPGSVRLPSQTQNLYNAQGNIIAKGIYDSTTNTTTYTFTNYVDQYTNVSGS      120
                   **************************************.****************** **

                                                           Plasmin
SdrC_Mu50          FEQVAFAKRENATTDKTAYKMEVTLGNDTYSKDVIVDYGNQKGQQLISSTNYINNEDLSR      180
SdrC_N315          FEQVAFAKRENATTDKTAYKMEVTLGNDTYSKDVIVDYGNQKGQQLISSTNYINNEDLSR      180
SdrC_ST228         FEQVAFAKRENATTDKTAYKMEVTLGNDTYSKDVIVDYGNQKGQQLISSTNYINNEDLSR      180
SdrC_COL           FEQVAFAKRKNATTDKTAYKMEVTLGNDTYSEEIIVDYGNKKAQPLISSTNYINNEDLSR      180
SdrC_NCTC8325      FEQVAFAKRKNATTDKTAYKMEVTLGNDTYSEEIIVDYGNKKAQPLISSTNYINNEDLSR      180
SdrC_MW2           FEQVAFAKRENATTDKTAYKMEVSLGNDTYSEEIIVDYGNKKAQPLISSTNYINNEDLSR      180
SdrC_MSSA276       FEQVAFAKRENATTDKTAYKMEVSLGNDTYSEEIIVDYGNKKAQPLISSTNYINNEDLSR      180
SdrC_ISU926        FEQVAFAKRENATTDKTAYKMEVTLGNDAYSEEIIVDYGNKKAQPLISSTNYINNEDLSR      180
SdrC_MRSA252       FEQVAFAKRENATTDKTAYKMEVTLGNDAYSEEIIVDYGNKKAQPLISSTNYINNEDLSR      180
SdrC_ATCC25923     FEQVAFAKRENATTDKTAYKMEVTLGNDAYSEEIIVDYGNKKAQPLISSTNYINNEDLSR      180
SdrC_TCH60         FEQVAFAKRENATTDKTAYKMEVTLGNDVYSEEIIVDYGNKKAQPLISSTNYINNEDLSR      180
                   *********:*************:****.**:::******:*.* ***************

SdrC_Mu50          NMTVYYVNQPKKTYTKETFVTNLTGYKFNPDAKNFKIYEVTDQNQFVDSFTPDTSKLKDVT      240
SdrC_N315          NMTVYYVNQPKKTYTKETFVTNLTGYKFNPDAKNFKIYEVTDQNQFVDSFTPDTSKLKDVT      240
SdrC_ST228         NMTVYYVNQPKKTYTKETFVTNLTGYKFNPDAKNFKIYEVTDQNQFVDSFTPDTSKLKDVT      240
SdrC_COL           NMTAYVNQPKNTYTKQTFVTNLTGYKFNPNAKNFKIYEVTDQNQFVDSFTPDTSKLKDVT      240
SdrC_NCTC8325      NMTAYVNQPKNTYTKQTFVTNLTGYKFNPNAKNFKIYEVTDQNQFVDSFTPDTSKLKDVT      240
SdrC_MW2           NMTAYVNQPKNTYTKQTFVTNLTGYKFNPNAKNFKIYEVTDQNQFVDSFTPDTSKLKDVT      240
SdrC_MSSA276       NMTAYVNQPKNTYTKQTFVTNLTGYKFNPNAKNFKIYEVTDQNQFVDSFTPDTSKLKDVT      240
SdrC_ISU926        NMTVYVNQPKNTYTKETFVSTLTGYKFNPDAKNFKIYEVTDQNQFVDSFTPDTSKLKEVT      240
SdrC_MRSA252       NMTVYVNQPKNTYTKETFVSTLTGYKFNPDAKNFKIYEVTDQNQFVDSFTPDTSKLIDVT      240
SdrC_ATCC25923     NMTVYVNQPKNTYTKETFVSTLTGYKFNPDAKNFKIYEVTDQNQFVDSFTPDTSKLIDVT      240
SdrC_TCH60         NMTVYVNQPKNTYTKETFVSTLTGYKFNPDAKNFKIYEVTDQNQFVDSFTPDTSKLIDVT      240
                   ***.******:****:***:.********:************************** :**

SdrC_Mu50          GQFDVIYSNDNKTATVDLLNGQSSSDKQYIIQQVAYPDNSSTDNGKIDYTLETQNGKSSW      300
SdrC_N315          GQFDVIYSNDNKTATVDLLNGQSSSDKQYIIQQVAYPDNSSTDNGKIDYTLETQNGKSSW      300
SdrC_ST228         GQFDVIYSNDNKTATVDLLNGQSSSDKQYIIQQVAYPDNSSTDNGKIDYTLETQNGKSSW      300
SdrC_COL           DQFDVIYSNDNKTATVDLMKGQTSSNKQYIIQQVAYPDNSSTDNGKIDYTLDTDKTKYSW      300
SdrC_NCTC8325      DQFDVIYSNDNKTATVDLMKGQTSSNKQYIIQQVAYPDNSSTDNGKIDYTLDTDKTKYSW      300
SdrC_MW2           NQFNITYSNDNKTATVDLMNGQTSSNKQYIIQQVAYPDNTSTDNGKIDYTLDTDKTKYSW      300
SdrC_MSSA276       NQFNITYSNDNKTATVDLMNGQTSSNKQYIIQQVAYPDNTSTDNGKIDYTLDTDKTKYSW      300
SdrC_ISU926        NQFNITYSNDNKTATVDLLNGQTSSNKQYIIQQVAYPDNTSTDNGKIDYTLETQNGKSSW      300
SdrC_MRSA252       DKFKITYSNDNKTATVDLMNGQTNSNKQYIIQQVAYPDNTSTDNGKIDYTLDTDKTKYSW      300
SdrC_ATCC25923     DKFKITYSNDNKTATVDLMNGQTNSNKQYIIQQVAYPDNTSTDNGKIDYTLDTDKTKYSW      300
SdrC_TCH60         DKFKITYSNDNKTATVDLMNGQTNSNKQYIIQQVAYPDNTSTDNGKIDYTLDTDKTKYSW      300
                   .:*.: ************::**:.*:*************:***********:*:: * **
```

```
                                  Plasmin & Plasmin SAK
SdrC_Mu50           SNSYSNVNGSSTANGDQKK        319
SdrC_N315           SNSYSNVNGSSTANGDQKK        319
SdrC_ST228          SNSYSNVNGSSTANGDQKK        319
SdrC_COL            SNSYSNVNGSSTANGDQKK        319
SdrC_NCTC8325       SNSYSNVNGSSTANGDQKK        319
SdrC_MW2            SNSYSNVNGSSTANGDQKK        319
SdrC_MSSA276        SNSYSNVNGSSTANGDQKK        319
SdrC_ISU926         SNSYSSVNGSSTANGDQKK        319
SdrC_MRSA252        SNSYSSVNGSSTANGDQKK        319
SdrC_ATCC25923      SNSYSSVNGSSTANGDQKK        319
SdrC_TCH60          SNSYSSVNGSSTANGDQKK        319
                    *****.*************
```

Figure S14. Sequence alignment of SdrC N2-N3 domains of different *S. aureus* strains.

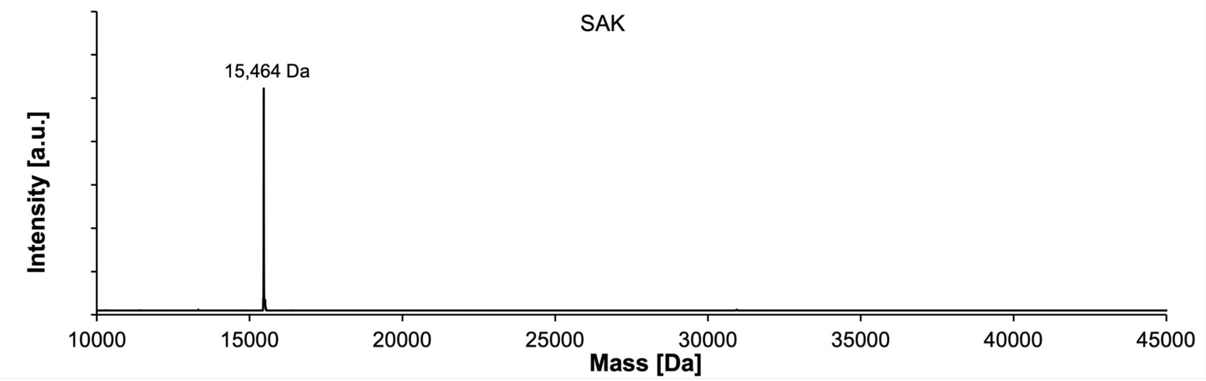

Sequence of undigested SAK:

```
        10          20          30          40          50          60
SSSFDKGKYK  KGDDASYFEP  TGPYLMVNVT  GVDGKGNELL  SPHYVEFPIK  PGTTLTKEKI

        70          80          90         100         110         120
EYYVEWALDA  TAYKEFRVVE  LDPSAKIEVT  YYDKNKKKEE  TKSFPITEKG  FVVPDLSEHI

       130
KNPGFNLITK VVIEKK
```

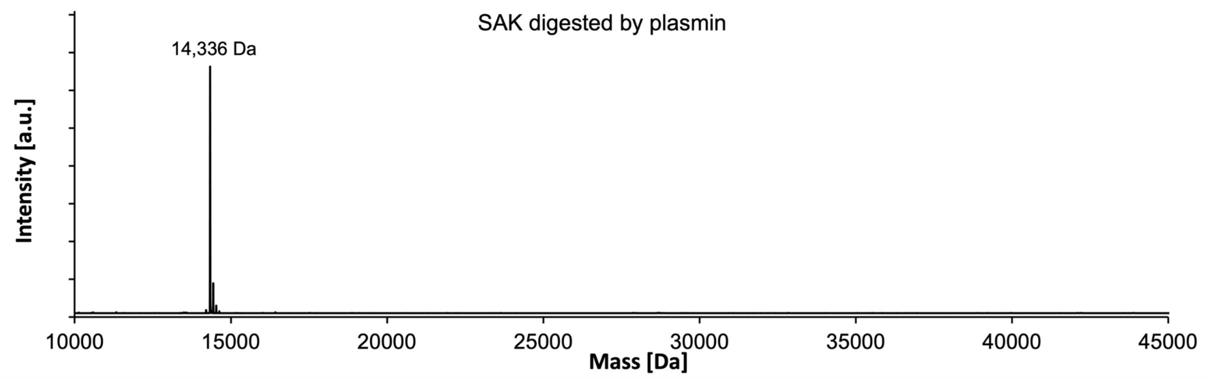

Determination of digested SAK sequence:

```
        10          20          30          40          50          60
KGDDASYFEP  TGPYLMVNVT  GVDGKGNELL  SPHYVEFPIK  PGTTLTKEKI  EYYVEWALDA

        70          80          90         100         110         120
TAYKEFRVVE  LDPSAKIEVT  YYDKNKKKEE  TKSFPITEKG  FVVPDLSEHI  KNPGFNLITK


VVIEKK
```

Figure S15. LC-MS spectra and sequence determination of SAK and SAK digested by plasmin overnight.

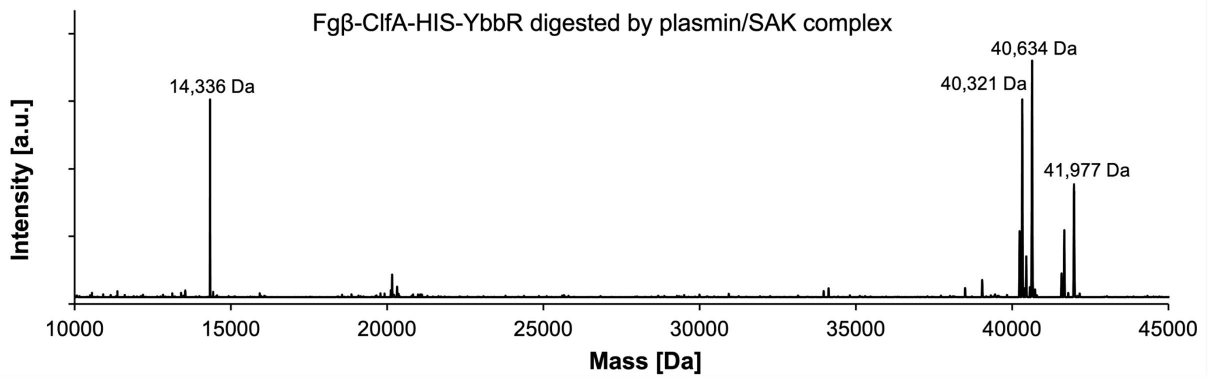
Fgβ-ClfA-HIS-YbbR digested by plasmin/SAK complex
Intensity [a.u.]
14,336 Da
40,634 Da
40,321 Da
41,977 Da
10000
15000
20000
25000
30000
35000
40000
45000
Mass [Da]

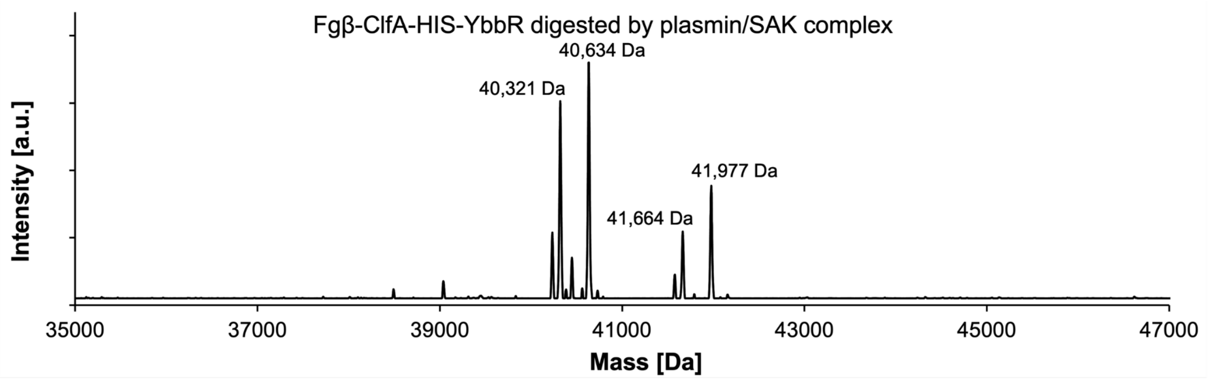
Fgβ-ClfA-HIS-YbbR digested by plasmin/SAK complex
Intensity [a.u.]
40,634 Da
40,321 Da
41,977 Da
41,664 Da
35000
37000
39000
41000
43000
45000
47000
Mass [Da]

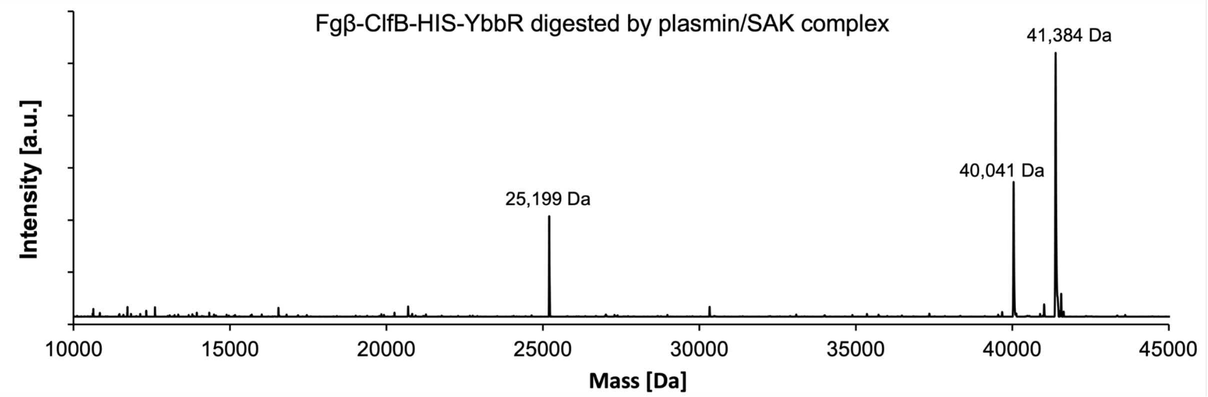
Fgβ-ClfB-HIS-YbbR digested by plasmin/SAK complex
Intensity [a.u.]
41,384 Da
40,041 Da
25,199 Da
10000
15000
20000
25000
30000
35000
40000
45000
Mass [Da]

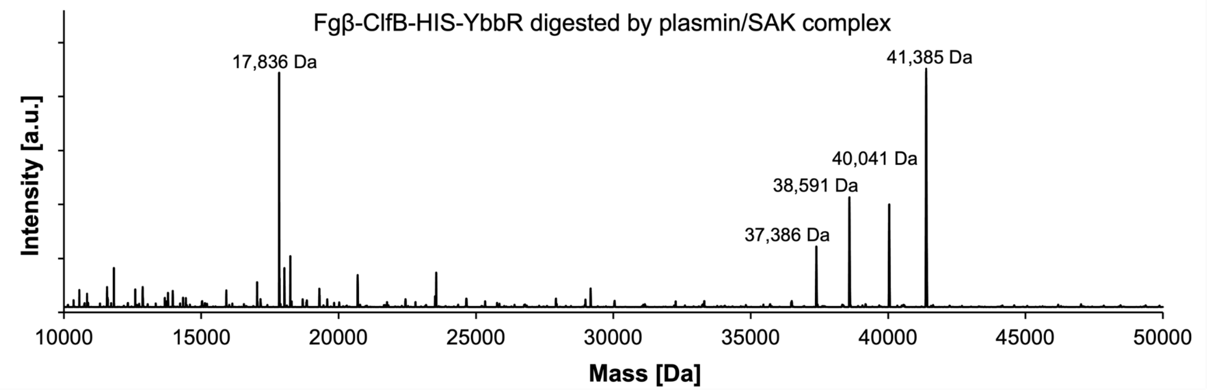
Fgβ-ClfB-HIS-YbbR digested by plasmin/SAK complex
Intensity [a.u.]
17,836 Da
41,385 Da
40,041 Da
38,591 Da
37,386 Da
10000
15000
20000
25000
30000
35000
40000
45000
50000
Mass [Da]

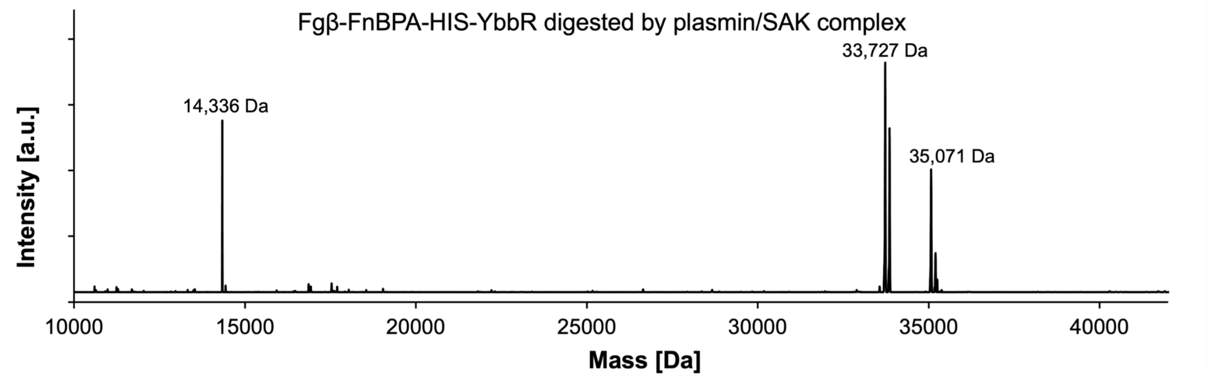
Fgβ-FnBPA-HIS-YbbR digested by plasmin/SAK complex
14,336 Da
33,727 Da
35,071 Da
Intensity [a.u.]
Mass [Da]
10000
15000
20000
25000
30000
35000
40000

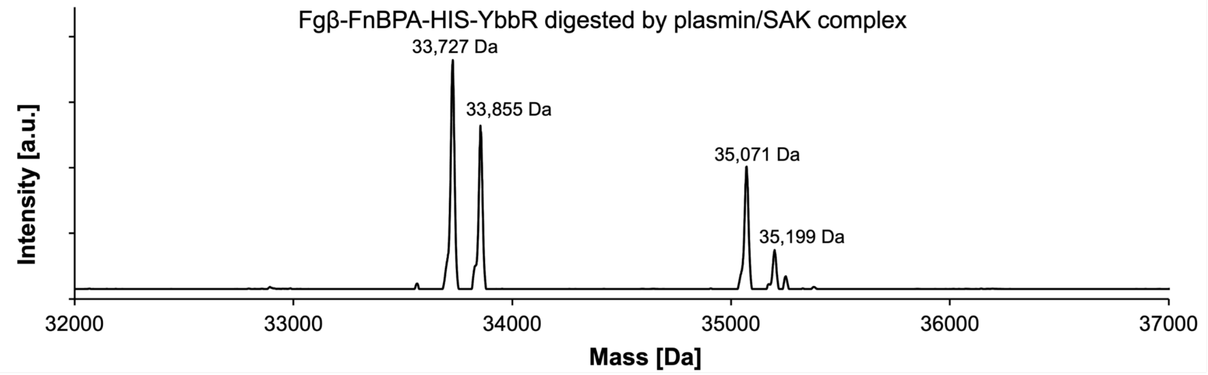
Fgβ-FnBPA-HIS-YbbR digested by plasmin/SAK complex
33,727 Da
33,855 Da
35,071 Da
35,199 Da
Intensity [a.u.]
Mass [Da]
32000
33000
34000
35000
36000
37000

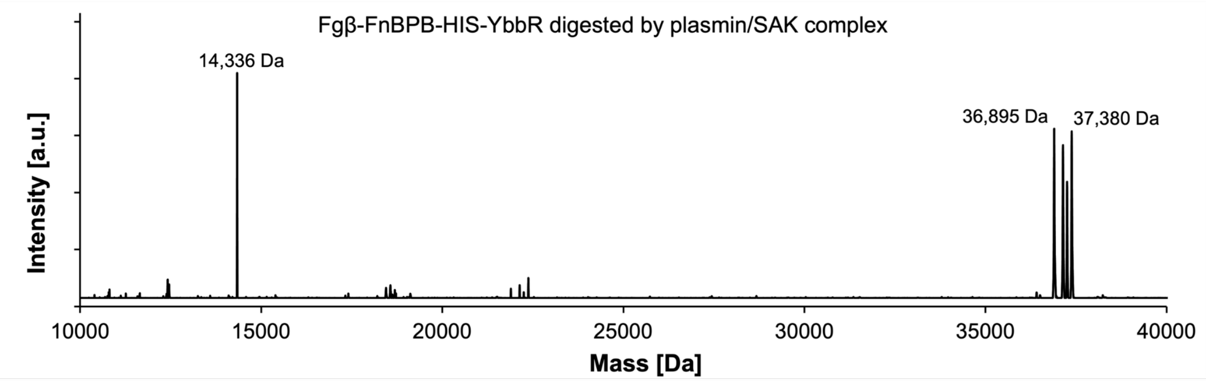
Fgβ-FnBPB-HIS-YbbR digested by plasmin/SAK complex
14,336 Da
36,895 Da
37,380 Da
Intensity [a.u.]
Mass [Da]
10000
15000
20000
25000
30000
35000
40000

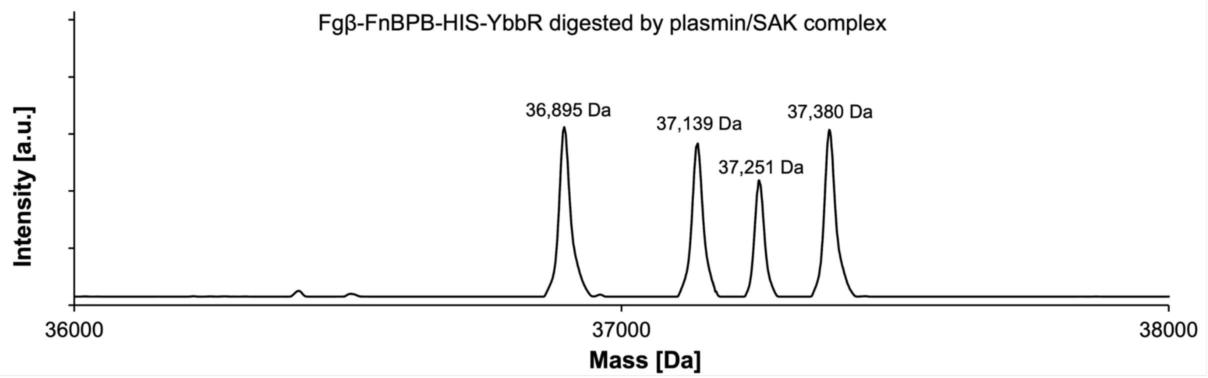
Fgβ-FnBPB-HIS-YbbR digested by plasmin/SAK complex
36,895 Da
37,139 Da
37,251 Da
37,380 Da
Intensity [a.u.]
Mass [Da]
36000
37000
38000

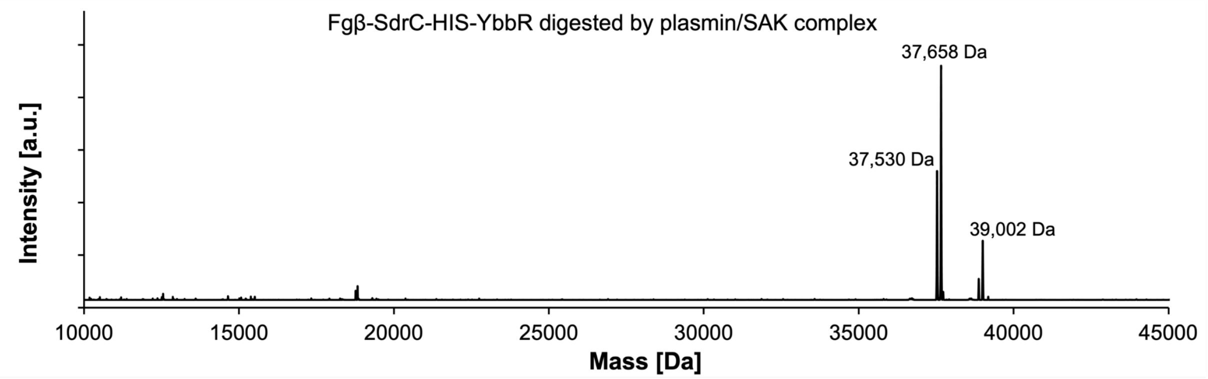
Fgβ-SdrC-HIS-YbbR digested by plasmin/SAK complex
37,658 Da
37,530 Da
39,002 Da
Intensity [a.u.]
Mass [Da]
10000
15000
20000
25000
30000
35000
40000
45000

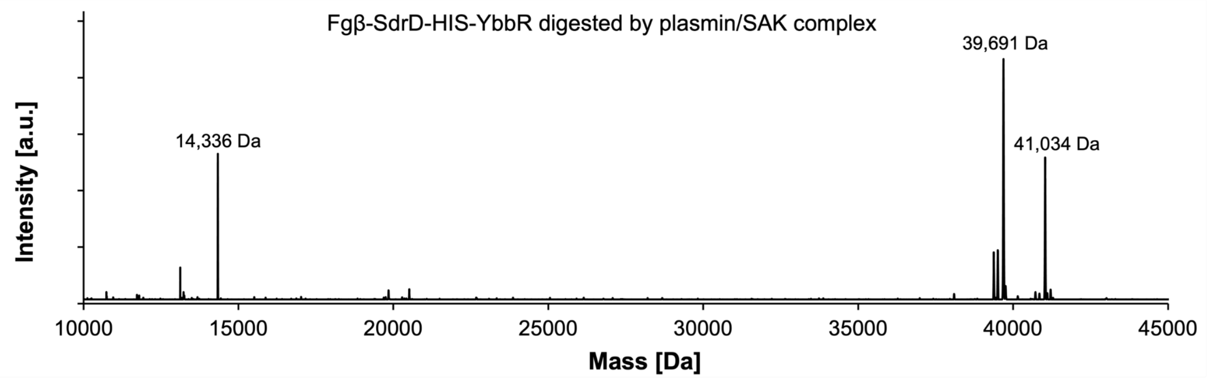
Fgβ-SdrD-HIS-YbbR digested by plasmin/SAK complex
39,691 Da
14,336 Da
41,034 Da
Intensity [a.u.]
Mass [Da]
10000
15000
20000
25000
30000
35000
40000
45000

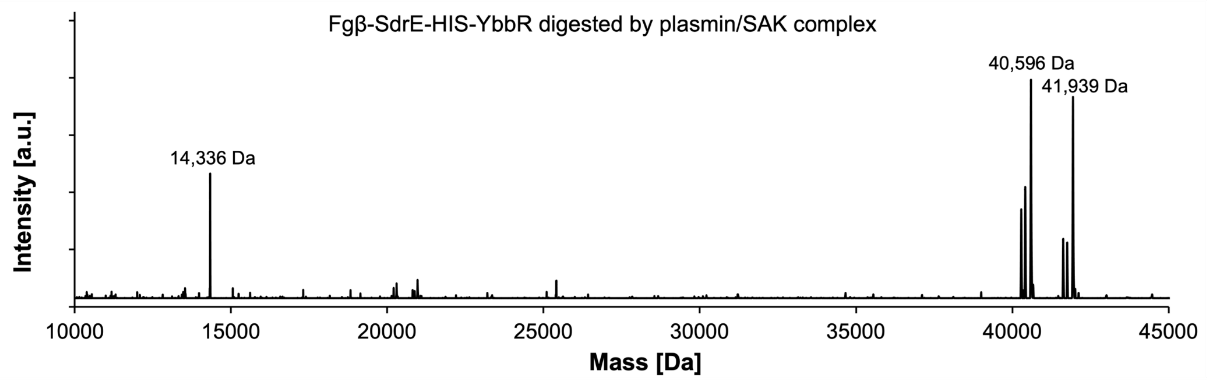
Fgβ-SdrE-HIS-YbbR digested by plasmin/SAK complex
40,596 Da
41,939 Da
14,336 Da
Intensity [a.u.]
Mass [Da]
10000
15000
20000
25000
30000
35000
40000
45000

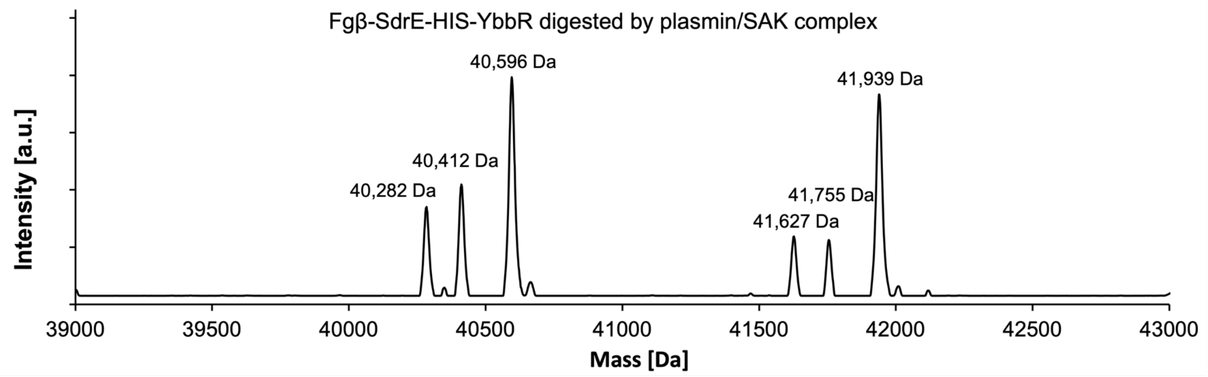
Fgβ-SdrE-HIS-YbbR digested by plasmin/SAK complex
40,596 Da
41,939 Da
40,412 Da
40,282 Da
41,755 Da
41,627 Da
Intensity [a.u.]
Mass [Da]
39000
39500
40000
40500
41000
41500
42000
42500
43000

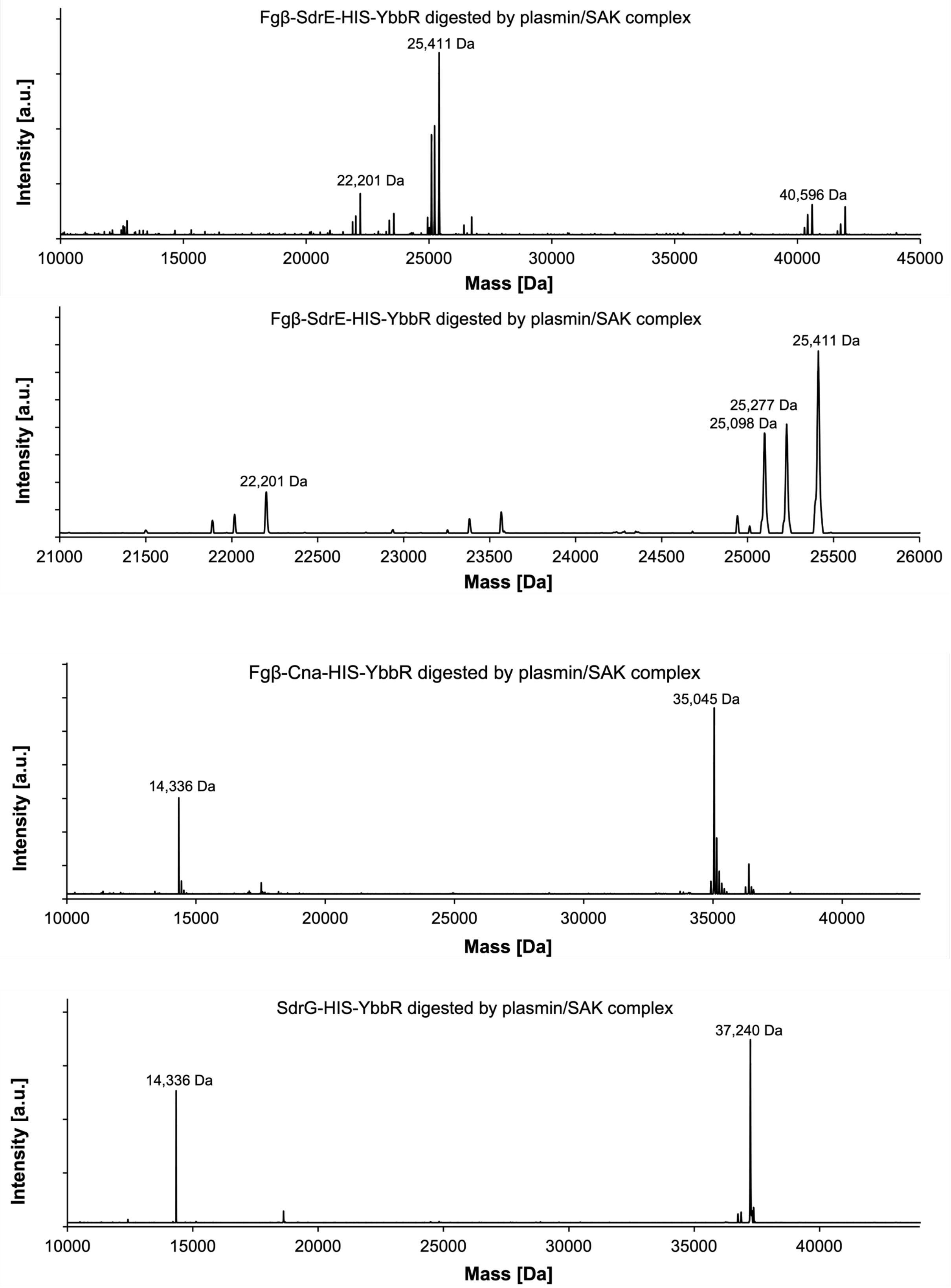

Figure S16. LC-MS spectra of plasmin/SAK digested adhesins.

```
SdrE_MRSA252        VASNNVNDLITVTKQMITEGIKDDGVIQAHDGEHIIYTSDFKIDNAVKAGDTMTVKYDKH      60
SdrE_ATCC25923      VASNNVNDLITVTKQMITEGIKDDGVIQAHDGEHIIYTSDFKIDNAVKAGDTMTVKYDKH      60
SdrE_TCH60          VASNNVNDLITVTKQMITEGIKDDGVIQAHDGEHIIYTSDFKIDNAVKAGDTMTVKYDKH      60
SdrE_ST228          VASNNVNDLITVTKQMITEGIKDDGVIQAHDGEHIIYTSDFKIDNAVKAGDTMTVKYDKH      60
SdrE_ISU926         VASNNVNDLIKVTKQTIKVGDGKDDVVAAHDGEEIEYDSEFTIDNKVKAGDTMTINYDKN      60
Sdre_Mu50           VASNNVNDLITVTKQTIKVGDGKDNVAAAHDGKDIEYDTEFTIDNKVKKGDTMTINYDKN      60
SdrE_N315           VASNNVNDLITVTKQTIKVGDGKDNVAAAHDGKDIEYDTEFTIDNKVKKGDTMTINYDKN      60
SdrE_COL            VASNNVNDLIKVTKQTIKVGDGKDNVAAAHDGKDIEYDTEFTIDNKVKKGDTMTINYDKN      60
SdrE_MW2            VASNNVNDLIKVTKQTIKVGDGKDNVAAAHDGKDIEYDTEFTIDNKVKKGDTMTINYDKN      60
SdrE_MSSA276        VASNNVNDLIKVTKQTIKVGDGKDNVAAAHDGKDIEYDTEFTIDNKVKKGDTMTINYDKN      60
                    **********.**** *. *  .*.*  ****:.* * ::*.*** ** *****::***:

SdrE_MRSA252        TIPSDITDDFTPVDITDPSGEVIAKGTFDLNTKTITYKFTDYVDRYENVNAKLELNSYID      120
SdrE_ATCC25923      TIPSDITDDFTPVDITDPSGEVIAKGTFDLNTKTITYKFTDYVDRYENVNAKLELNSYID      120
SdrE_TCH60          TIPSDITDDFTPVDITDPSGEVIAKGTFDLNTKTITYKFTDYVDRYENVNAKLELNSYID      120
SdrE_ST228          TIPSDITDDFTPVDITDPSGEVIAKGTFDLNTKTITYKFTDYVDRYENVNAKLELNSYID      120
SdrE_ISU926         VIPSDLTDKNDPIDITDPSGEVIAKGTFDKATKQITYTFTDYVDKYEDIKSRLTLYSYID      120
Sdre_Mu50           VIPSDLTDKNDPIDITDPSGEVIAKGTFDKATKQITYTFTDYVDKYEDIKARLTLYSYID      120
SdrE_N315           VIPSDLTDKNDPIDITDPSGEVIAKGTFDKATKQITYTFTDYVDKYEDIKARLTLYSYID      120
SdrE_COL            VIPSDLTDKNDPIDITDPSGEVIAKGTFDKATKQITYTFTDYVDKYEDIKSRLTLYSYID      120
SdrE_MW2            VIPSDLTDKNDPIDITDPSGEVIAKGTFDKATKQITYTFTDYVDKYEDIKSRLTLYSYID      120
SdrE_MSSA276        VIPSDLTDKNDPIDITDPSGEVIAKGTFDKATKQITYTFTDYVDKYEDIKSRLTLYSYID      120
                    .****:**.  *:****************  ** ***.******:**:::::* * ****

                    Plasmin/SAK
SdrE_MRSA252        KKEVPNETNLNLTFATADKETSKNVKVEYQKPIVKDESNIQSIFSHLDTTKHEVEQTIYV      180
SdrE_ATCC25923      KKEVPNETNLNLTFATADKETSKNVKVEYQKPIVKDESNIQSIFSHLDTTKHEVEQTIYV      180
SdrE_TCH60          KKEVPNETNLNLTFATADKETSKNVKVEYQKPIVKDESNIQSIFSHLDTTKHEVEQTIYV      180
SdrE_ST228          KKEVPNETNLNLTFATADKETSKNVKVEYQKPIVKDESNIQSIFSHLDTTKHEVEQTIYV      180
SdrE_ISU926         KKTVPNETSLNLTFATAGKETSQNVTVDYQDPMVHGDSNIQSIFTKLDEDKQNIEQQIYV      180
Sdre_Mu50           KQAVPNETSLNLTFATAGKETSQNVSVDYQDPMVHGDSNIQSIFTKLDENKQTIEQQIYV      180
SdrE_N315           KQAVPNETSLNLTFATAGKETSQNVSVDYQDPMVHGDSNIQSIFTKLDENKQTIEQQIYV      180
SdrE_COL            KKTVPNETSLNLTFATAGKETSQNVTVDYQDPMVHGDSNIQSIFTKLDEDKQTIEQQIYV      180
SdrE_MW2            KKTVPNETSLNLTFATAGKETSQNVTVDYQDPMVHGDSNIQSIFTKLDEDKQTIEQQIYV      180
SdrE_MSSA276        KKTVPNETSLNLTFATAGKETSQNVTVDYQDPMVHGDSNIQSIFTKLDEDKQTIEQQIYV      180
                    *: *****.********.****:**.*:**.*:*:.:*******::**  *: :** ***

SdrE_MRSA252        NPLKLNAKNTNVTIKSGGVADNGDYYTGDGSTIIDSNTEIKVYKVASGQQLPQSNKIYDY      240
SdrE_ATCC25923      NPLKLNAKNTNVTIKSGGVADNGDYYTGDGSTIIDSNTEIKVYKVASGQQLPQSNKIYDY      240
SdrE_TCH60          NPLKLNAKNTNVTIKSGGVADNGDYYTGDGSTIIDSNTEIKVYKVASGQQLPQSNKIYDY      240
SdrE_ST228          NPLKLNAKNTNVTIKSGGVADNGDYYTGDGSTIIDSNTEIKVYKVASGQQLPQSNKIYDY      240
SdrE_ISU926         NPLKKTATNTKVDIAGSQVDDYGNIKLGNGSTIIDQNTEIKVYKVNSNQQLPQSNRIYDF      240
Sdre_Mu50           NPLKKTATNTKVDIAGSQVDDYGNIKLGNGSTIIDQNTEIKVYKVNPNQQLPQSNRIYDF      240
SdrE_N315           NPLKKTATNTKVDIAGSQVDDYGNIKLGNGSTIIDQNTEIKVYKVNPNQQLPQSNRIYDF      240
SdrE_COL            NPLKKSATNTKVDIAGSQVDDYGNIKLGNGSTIIDQNTEIKVYKVNSDQQLPQSNRIYDF      240
SdrE_MW2            NPLKKSATNTKVDIAGSQVDDYGNIKLGNGSTIIDQNTEIKVYKVNSDQQLPQSNRIYDF      240
SdrE_MSSA276        NPLKKSATNTKVDIAGSQVDDYGNIKLGNGSTIIDQNTEIKVYKVNSDQQLPQSNRIYDF      240
                    ****  .*.**:* *  .. * * *:   *:******.*********   .*******:***:

SdrE_MRSA252        SQYEDVTNSVTINKNYGTNMANINFGDIDSAYIVKVVSKYTPGAEDDLAVQQGVRMTTTN      300
SdrE_ATCC25923      SQYEDVTNSVTINKNYGTNMANINFGDIDSAYIVKVVSKYTPGAEDDLAVQQGVRMTTTN      300
SdrE_TCH60          SQYEDVTNSVTINKNYGTNMANINFGDIDSAYIVKVVSKYTPGAEDDLAVQQGVRMTTTN      300
SdrE_ST228          SQYEDVTNSVTINKNYGTNMANINFGDIDSAYIVKVVSKYTPGAEDDLAVQQGVRMTTTN      300
SdrE_ISU926         SQYEDVTSQFDNKKSFSNNVATLDFGNIDSAYIIKVVSKYTPTSDDELDIAQGASMRTTD      300
Sdre_Mu50           SQYEDVTSQFDNKKSFSNNVATLDFGDINSAYIIKVVSKYTPTSDGELDIAQGTSMRTTD      300
SdrE_N315           SQYEDVTSQFDNKKSFSNNVATLDFGDINSAYIIKVVSKYTPTSDGELDIAQGTSMRTTD      300
SdrE_COL            SQYEDVTSQFDNKKSFSNNVATLDFGDINSAYIIKVVSKYTPTSDGELDIAQGTSMRTTD      300
SdrE_MW2            SQYEDVTSQFDNKKSFSNNVATLDFGDINSAYIIKVVSKYTPTSDGELDIAQGTSMRTTD      300
SdrE_MSSA276        SQYEDVTSQFDNKKSFSNNVATLDFGDINSAYIIKVVSKYTPTSDGELDIAQGTSMRTTD      300
                    *******...  :*.:..*:*.::**:*:****:******** ::.:* : **. * **:

SdrE_MRSA252        KYNYSSYAGYTNTILSTTDSGGGDGTVKPE      330
SdrE_ATCC25923      KYNYSSYAGYTNTILSTTDSGGGDGTVKPE      330
SdrE_TCH60          KYNYSSYAGYTNTILSTTDSGGGDGTVKPE      330
SdrE_ST228          KYNYSSYAGYTNTILSTTDSGGGDGTVKPE      330
SdrE_ISU926         KNNYYNYAGYSNFIVTSTDTGGGDGTVKPE      330
Sdre_Mu50           KYGYYNYAGYSNFIVTSNDTGGGDGTVKPE      330
SdrE_N315           KYGYYNYAGYSNFIVTSNDTGGGDGTVKPE      330
SdrE_COL            KYGYYNYAGYSNFIVTSNDTGGGDGTVKPE      330
SdrE_MW2            KYGYYNYAGYSNFIVTSNDSGGGDGTVKPE      330
SdrE_MSSA276        KYGYYNYAGYSNFIVTSNDSGGGDGTVKPE      330
                    * .* .****:* *:::.*:**********
```

Figure S17. Sequence alignment of SdrE N2-N3 domains of different *S. aureus* strains.

```
Cna_NCTC6131        -------ARDISSTNVTDLTVSP-SKIE------------------DGGKTTVKMTFDDK      34
ClfA_NCTC8325       ----VAADAPVAGTDITNQLTNV-----TV-----GIDSGTTVYPHQAGYVKLNYGFSV-      45
FnBPA_NCTC8325      ------------GTDVTSKVTVEIGSI--E-----GHNNTNKVEPHAGQRAVLKYKLKF-      40
FnBPB_NCTC8325      ------------GTDVTNKVEVEEGSEIVG-----HKQDTNVVNPHNAERVTLKYKWKF-      42
SdrC_NCTC8325       --------APQQGTNVNDKVHFSNIDIAIDKGHVNQTTGKTEFWATSSDVLKLKANYTI-      51
ClfB_N315           MGTPVVNAADAKGTNVNDKVTASNFKLEKT-----------TFDPNQSGNTFMAANFTV-      48
SdrE_Mu50           ----------VASNNVNDLITVTKQTIKVGDGK------DNVAAAHDGKDIEYDTEFTI-      43
SdrD_NCTC8325       ------------AKNVNDLITSNTTLTVV-DAD------KNNKIVPAQDYLSLKSQITV-      40
                                ..::..

Cna_NCTC6131        NGKIQNGDMIKVAWPTSGTVKIE-GYSKTV------PL--TVKGEQVGQAVITPDG--AT      83
ClfA_NCTC8325       PNSAVKGDTFKITVPKELNLNGVTSTAKVP------PIM-AG-DQVLANGVIDSDG-NVI      96
FnBPA_NCTC8325      ENGLHQGDYFDFTLSNNVNTHGVSTARKVP------EIK-NG-SVVMATGEVLEGG-KIR      91
FnBPB_NCTC8325      GEGIKAGDYFDFTLSDNVETHGISTLRKVP------EIK-STDGQVMATGEIIGER-KVR      94
SdrC_NCTC8325       DDSVKEGDTFTFKYGQYFRPGSVRLPSQTQ------NL-YNAQGNIIAKGIYDSTTNTTT      104
ClfB_N315           TDKVKSGDYFTAKLPDSLTGNGDVDYSNSNNTMPIADIK-STNGDVVAKATYDILTKTYT      107
SdrE_Mu50           DNKVKKGDTMTINYDKNVIPSDLTD------KNDPIDITD-PSGEVIAKGTFDKATKQIT      96
SdrD_NCTC8325       DDKVKSGDYFTIKYSDTVQVYGLNPE----DIKNIGDIKDPNNGETIATAKHDTANNLIT      96
                          ** :                           :     .  :. .

Cna_NCTC6131        ITFNDKVEKLSDVSGFAEFEVQGRNLTQTNTSDDKVATITSGNKSTNVTVHKSEAGTSSV      143
ClfA_NCTC8325       YTFTDYVNTKDDVKATLTMPAYIDPENVKKTGNV-TLATGIGSTTANKTVLVDYE-KYGK      154
FnBPA_NCTC8325      YTFTNDIEDKVDVTAELEINLFIDPKTVQTNGNQ-TITSTLNEEQTSKELDVKYKDGIGN      150
FnBPB_NCTC8325      YTFKEYVQEKKDLTAELSLNLFIDPTTVTQKGNQ-NVEVKLGETTVSKIFNIQYLGGVRD      153
SdrC_NCTC8325       YTFTNYVDQYTNVRGSFEQVAFAKRKNATTDKTAYKMEVTLGNDTYSEEIIVDYGNKKAQ      164
ClfB_N315           FVFTDYVNNKENINGQFSLPLFTDRAKAPKSGTY-DANINIADEMFNNKITYNYSSPIAG      166
SdrE_Mu50           YTFTDYVDKYEDIKARLTLYSYIDKQAVPNETSL-NLTFATAGKETSQNVSVDYQDPMVH      155
SdrD_NCTC8325       YTFTDYVDRFNSVQMGINYSIYMDADTIPVSKNDVEFNVTIGNTTTKTTANIQYPDYVVN      156
                     .*.: ::   .:                                 .     .

Cna_NCTC6131        FYYKTGDMLPEDTTH---------VR--WFLNINNEKSYVSKDITIKDQIQGGQQLDLST      192
ClfA_NCTC8325       FY---NLSIKGTIDQID--KTNNTYRQTIYVNP-SGDNVIAPVLT------GNLKP----      198
FnBPA_NCTC8325      Y----YANLNGSIETFN--KANNRFSHVAFIKP-NNGKTTSVTVT------GTLMK----      193
FnBPB_NCTC8325      NW---GVTANGRIDTLN--KVDGKFSHFAYMKP-NNQSLSSVTVT------GQVTK----      197
SdrC_NCTC8325       PL------ISSTNY-IN--NEDLSRNMTAYVNQ-PKNTYTKQTFV---------------      199
ClfB_N315           IDKPNGANISSQIIGVDTASGQNTYKQTVFVNP-KQRVLGNTWVYIK----GYQDK----      217
SdrE_Mu50           GDSNIQSIFTKL------DENKQTIEQQIYVNP-LKKTATNTKVDIA----GSQVDDYGN      204
SdrD_NCTC8325       EKNSIGSAFTETVSHVGNKENPGYYKQTIYVNP-SENSLTNAKLKVQ----AYHSS----      207
                                             :::           .

Cna_NCTC6131        LN------INVTGTHSNYYSGQSAITDFEKAFPGSKITVDNTKNTIDVTI----PQGYGS      242
ClfA_NCTC8325       -NTDSNALIDQQNTSIKVYKV-DNAADLSESYF---VN---PENFEDVTNSV--NITFPN      248
FnBPA_NCTC8325      -GSNQNG----NQPKVRIFEYLGNNEDIAKSVY---ANTTDTSKFKEVTSNMSGNLNLQN      245
FnBPB_NCTC8325      -GNKPGV----NNPTVKVYKHIGS-DDLAESVY---AKLDDVSKFEDVTDNMSL--DFDT      246
SdrC_NCTC8325       -TNLTGYKFNPNAKNFKIYEVTDQ-NQFVDSFT---P---DTSKLKDVTDQFDVIYS-ND      250
ClfB_N315           -IEESSGKVSATDTKLRIFEVNDT-SKLSDSYY---AD-PNDSNLKEVTDQFKNRIYYEH      271
SdrE_Mu50           IKLGNGSTIIDQNTEIKVYKVNPN-QQLPQSNR---I--YDFSQYEDVTSQFDNKKSF-S      257
SdrD_NCTC8325       -YPNNIGQINKDVTDIKIYQVPKG-YTLNKGYD---V---NTKELTDVTNQYLQKITYGD      259
                                . :.       : ..          .:  :**

Cna_NCTC6131        YNSFSINYKTKITNEQQKEFVNNSQAWYQEHGKEEV--------------------NGK      281
ClfA_NCTC8325       PNQYKVEFNTPDD-QITTPYIVVVNGHIDPNSKGDLALRSTLYGYNSNI------IWRSM      301
FnBPA_NCTC8325      NGSYSLNIENLD-----KTYVVHYDGEYLNG-TDEVDFRTQMVGHPEQLYKYYYDRGYTL      299
FnBPB_NCTC8325      NGGYSLNFNNLDQ---SKNYVIKYEGYYDSN-ASNLEFQTHLFG----YYNYYYT--SNL      296
SdrC_NCTC8325       NKTATVDLMKGQT-SSNKQYIIQQVAYPDNSSTDNGK--IDYTLDT-DK--------TKY      298
ClfB_N315           PNVASIKFGD-----ITKTYVVLVEGHYDNTGKNLKTQVIQENVDPVTNRD-----YSIF      321
SdrE_Mu50           NNVATLDFGD-----INSAYIIKVVSKYTPTSDGELDIAQGTSMRTTDKYGY----YNYA      308
SdrD_NCTC8325       NNSAVIDFGN-----ADSAYVVMVNTKFQYTNSESPTLVQMATLSSTGNK-------SVS      307
                         :.          . ::

Cna_NCTC6131        SFNHTVHNINANAGIEGTVKGELK--------------         305
ClfA_NCTC8325       SWDNEVAFNNGSGSGDGIDKPVVPEQPDEPGEIEPIPE         339
FnBPA_NCTC8325      TWDNGLVLYSNKANGNEKN-------------------         318
FnBPB_NCTC8325      TWKNGVAFYSNNAQGDGKDKLK----------------         318
SdrC_NCTC8325       SWSNSYSNVNGSSTANGDQKK-----------------         319
ClfB_N315           GWNNENVVRYGGGSADGDSAVELKLPRSR---------         350
SdrE_Mu50           GYSNFIVTSNDTGGGDGTVKPE----------------         330
SdrD_NCTC8325       T-GNALGFTNNQSGGAGQE-------------------         325
```

Figure S18. Alignment of the amino acid sequence of the MSCRAMMs binding region used in this study. The strains are given after the adhesins' names.
